# Maternal educational attainment in pregnancy and epigenome-wide DNA methylation changes in the offspring from birth until adolescence

**DOI:** 10.1038/s41380-023-02331-5

**Published:** 2023-12-05

**Authors:** Priyanka Choudhary, Giulietta S. Monasso, Ville Karhunen, Justiina Ronkainen, Giulia Mancano, Caitlin G. Howe, Zhongzheng Niu, Xuehuo Zeng, Weihua Guan, John Dou, Jason I. Feinberg, Charles Mordaunt, Giancarlo Pesce, Nour Baïz, Rossella Alfano, Dries S. Martens, Congrong Wang, Elena Isaevska, Elina Keikkala, Sanna Mustaniemi, Chris H. L. Thio, Eliza Fraszczyk, Elmar W. Tobi, Anne P. Starling, Marta Cosin-Tomas, Jose Urquiza, Stefan Röder, Thanh T. Hoang, Christian Page, Dereje D. Jima, John S. House, Rachel L. Maguire, Raffael Ott, Xenia Pawlow, Lea Sirignano, Lea Zillich, Anni Malmberg, Sebastian Rauschert, Phillip Melton, Tong Gong, Robert Karlsson, Ruby Fore, Wei Perng, Zachary M. Laubach, Darina Czamara, Gemma Sharp, Carrie V. Breton, Enrique Schisterman, Edwina Yeung, Sunni L. Mumford, M. Daniele Fallin, Janine M. LaSalle, Rebecca J. Schmidt, Kelly M. Bakulski, Isabella Annesi-Maesano, Barbara Heude, Tim S. Nawrot, Michelle Plusquin, Akram Ghantous, Zdenko Herceg, Lorenza Nisticò, Marina Vafeiadi, Manolis Kogevinas, Marja Vääräsmäki, Eero Kajantie, Harold Snieder, Eva Corpeleijn, Regine P. M. Steegers-Theunissen, Ivana V. Yang, Dana Dabelea, Serena Fossati, Ana C. Zenclussen, Gunda Herberth, Maria Magnus, Siri E. Håberg, Stephanie J. London, Monica Cheng Munthe-Kaas, Susan K. Murphy, Cathrine Hoyo, Anette-G Ziegler, Sandra Hummel, Stephanie H. Witt, Fabian Streit, Josef Frank, Katri Räikkönen, Jari Lahti, Rae-chi Huang, Catarina Almqvist, Marie-France Hivert, Vincent W. V. Jaddoe, Marjo-Riitta Järvelin, Marko Kantomaa, Janine F. Felix, Sylvain Sebert

**Affiliations:** 1 Research Unit of Population Health, Faculty of Medicine, University of Oulu, 90014 Oulu, Finland; 2 The Generation R Study Group, Erasmus MC, University Medical Center Rotterdam, Rotterdam, the Netherlands; 3 Department of Pediatrics, Erasmus MC, University Medical Center Rotterdam, Rotterdam, the Netherlands; 4 Research Unit of Mathematical Sciences, Faculty of Science, University of Oulu, Oulu, Finland; 5 Medical Research Council Integrative Epidemiology Unit, Bristol Medical School, University of Bristol, Bristol, UK; 6 Bristol Medical School Population Health Sciences, University of Bristol, Bristol, UK; 7 Department of Epidemiology, Geisel School of Medicine, Dartmouth College, Hanover, NH USA; 8 Department of Population and Public Health Sciences, USC Keck School of Medicine, University of Southern California, Los Angeles, CA USA; 9 Glotech, Inc., Rockville, MD 20850 USA; 10 Division of Biostatistics, School of Public Health, University of Minnesota, Minneapolis, MN 55455 USA; 11 Department of Epidemiology, School of Public Health, University of Michigan, Ann Arbor, MI USA; 12 Department of Mental Health, Bloomberg School of Public Health, Johns Hopkins University, Baltimore, MA USA; 13 Department of Medical Micriobiology and Immunology, University of California Davis, Davis, CA USA; 14 Epidemiology of Allergic and Respiratory Diseases (EPAR) team, Faculté de Médecine Saint-Antoine, Institute Pierre Louis d’Epidemiologie et Sante Publique (IPLESP), Sorbonne Université and INSERM, Paris, France; 15 Paris-Saclay University, Paris-South University, UVSQ, Center for Research in Epidemiology and Population Health (CESP), INSERM, Villejuif, France; 16 Institute Desbrest of Epidemiology and Public Health, University of Montpellier and INSERM, Montpellier, France; 17 Centre for Environmental Sciences, Hasselt University, Diepenbeek, Belgium; 18 Cancer Epidemiology Unit, Department of Medical Sciences, University of Turin and CPO-Piemonte, Torino, Italy; 19 Department of Obstetrics and Gynaecology, Research Unit of Clinical Medicine, Medical Research Center, Oulu University Hospital, University of Oulu, Oulu, Finland; 20 Finnish Institute for Health and Welfare, Population Health Unit, Public Health and Welfare, Helsinki and Oulu, Finland; 21 Department of Epidemiology, University Medical Center Groningen, University of Groningen, Groningen, The Netherlands; 22 Department of Obstetrics and Gynaecology, Division of Obstetrics and Prenatal Medicine, Erasmus MC, University Medical Center, 3000 CA Rotterdam, the Netherlands; 23 Department of Epidemiology, Colorado School of Public Health, University of Colorado Anschutz Medical Campus, Aurora, CO USA; 24 Lifecourse Epidemiology of Adiposity and Diabetes (LEAD) Center, University of Colorado Anschutz Medical Campus, Aurora, CO USA; 25 Department of Epidemiology, University of North Carolina at Chapel Hill, Chapel Hill, NC USA; 26 ISGlobal (Barcelona Institute for Global Health), Barcelona Biomedical Research Park (PRBB), Doctor Aiguader, 88, 08003 Barcelona, Spain; 27 Universitat Pompeu Fabra (UPF), Barcelona, Spain; 28 CIBER Epidemiología y Salud Pública (CIBERESP), Madrid, Spain; 29 Department for Environmental Immunology, Helmholtz Centre for Environmental Research, UFZ, Leipzig, Germany; 30 Epidemiology Branch, Division of Intramural Research, National Institute of Environmental Health Sciences, National Institutes of Health, Research Triangle Park, NC USA; 31 Centre for Fertility and Health, Norwegian Institute of Public Health, Oslo, Norway; 32 Oslo Centre for Biostatistics and Epidemiology, Section for Research Support, Oslo University Hospital, Oslo, Norway; 33 Center for Human Health and the Environment, North Carolina State University, Raleigh, NC 27606 USA; 34 Bioinformatics Research Center, North Carolina State University, Raleigh, NC 27606 USA; 35 Biostatistics and Computational Biology Branch, National Institute of Environmental Health Sciences, National Institutes of Health, Department of Health and Human Services, Research Triangle Park, Durham, NC 27709 USA; 36 Department of Biological Sciences, North Carolina State University, Raleigh, NC USA; 37 Department of Obstetrics and Gynecology, Duke University Medical Center, Durham, NC 27701 USA; 38 Institute of Diabetes Research, Helmholtz Munich, German Research Center for Environmental Health, Munich, Germany; 39 Forschergruppe Diabetes eV, Neuherberg, Germany; 40 Department of Genetic Epidemiology in Psychiatry, Central Institute of Mental Health, Medical Faculty Mannheim, Heidelberg University, Mannheim, Germany; 41 Department of Psychology and Logopedics, Faculty of Medicine, University of Helsinki, Helsinki, Finland; 42 Telethon Kids Institute, Perth, WA Australia; 43 Menzies Institute of Medical Research, University of Tasmania, Hobart, TAS Australia; 44 University of Western Australia, School of Population and Global Health, Perth, WA Australia; 45 Department of Medical Epidemiology and Biostatistics, Karolinska Institutet, Stockholm, Sweden; 46 Division of Chronic Disease Research Across the Lifecourse (CoRAL), Department of Population Medicine, Harvard Medical School, Harvard Pilgrim Health Care Institute, Boston, MA USA; 47 Department of Epidemiology and the Lifecourse Epidemiology of Adiposity and Diabetes (LEAD) Center, University of Colorado Anschutz Medical Campus, Aurora, CO USA; 48 Department of Ecology and Evolutionary Biology, University of Colorado Boulder, Boulder, CO USA; 49 Department Genes and Environment, Max Planck Institute for Psychiatry, Kraepelinstrasse 2+10, 80804 Munich, Germany; 50 School of Psychology, Faculty of Health and Life Sciences, University of Exeter, Exeter, UK; 51 Department of Biostatistics, Epidemiology and Informatics, Perelman School of Medicine, University of Pennsylvania, Philadelphia, PA USA; 52 Epidemiology Branch, Division of Population Health Research, Division of Intramural Research, Eunice Kennedy Shriver National Institute of Child Health and Human Development, Bethesda, MD 20817 USA; 53 Department of Public Health Sciences, School of Medicine, University of California Davis (UC Davis), Davis, CA USA; 54 Université de Paris Cité, Inserm, INRAE, Centre of Research in Epidemiology and StatisticS (CRESS), F-75004 Paris, France; 55 Epigenomics and Mechanisms Branch, International Agency for Research on Cancer, Lyon, France; 56 Centre for Behavioural Sciences and Mental Health, Istituto Superiore di Sanità, Viale Regina Elena, Rome, Italy; 57 Department of Social Medicine, School of Medicine, University of Crete, Heraklion, Crete, Greece; 58 Barcelona Institute for Global Health (ISGlobal), Barcelona, Spain; 59 Centro de Investigación Biomédicaen Red de Epidemiología y Salud Pública (CIBERESP), Madrid, Spain; 60 Clinical Medicine Research Unit, Medical Research Center, Oulu University Hospital, University of Oulu, Oulu, Finland; 61 Children’s Hospital, University of Helsinki and Helsinki University Hospital, Helsinki, Finland; 62 Division of Biomedical Informatics and Personalized Medicine, Department of Medicine, University of Colorado School of Medicine, Aurora, CO USA; 63 Center for Genes, Environment and Health, National Jewish Health, Denver, CO USA; 64 Department of Pediatrics, School of Medicine, University of Colorado Anschutz Medical Campus, Aurora, CO USA; 65 Department of Pediatrics, Oncology and Hematology, Oslo University Hospital, Oslo, Norway; 66 Norwegian Institute of Public Health, Oslo, Norway; 67 Technical University Munich, School of Medicine, Forschergruppe Diabetes at Klinikum rechts der Isar, Munich, Germany; 68 Center for Innovative Psychiatric and Psychotherapeutic Research, Biobank, Central Institute of Mental Health, Medical Faculty Mannheim, Heidelberg University, Mannheim, Germany; 69 Edith Cowan University, School of Medicine and Health Sciences, Joondalup, WA Australia; 70 Pediatric Allergy and Pulmonology Unit at Astrid Lindgren Children’s Hospital, Karolinska University Hospital, Stockholm, Sweden; 71 Diabetes Unit, Massachusetts General Hospital, Boston, MA USA; 72 Department of Epidemiology and Biostatistics, MRC–PHE Centre for Environment & Health, School of Public Health, Imperial College London, London, UK; 73 Department of Life Sciences, College of Health and Life Sciences, Brunel University London, London, UK

**Keywords:** Genetics, Predictive markers

## Abstract

Maternal educational attainment (MEA) shapes offspring health through multiple potential pathways. Differential DNA methylation may provide a mechanistic understanding of these long-term associations. We aimed to quantify the associations of MEA with offspring DNA methylation levels at birth, in childhood and in adolescence. Using 37 studies from high-income countries, we performed meta-analysis of epigenome-wide association studies (EWAS) to quantify the associations of completed years of MEA at the time of pregnancy with offspring DNA methylation levels at birth (*n* = 9 881), in childhood (*n* = 2 017), and adolescence (*n* = 2 740), adjusting for relevant covariates. MEA was found to be associated with DNA methylation at 473 cytosine-phosphate-guanine sites at birth, one in childhood, and four in adolescence. We observed enrichment for findings from previous EWAS on maternal folate, vitamin-B_12_ concentrations, maternal smoking, and pre-pregnancy BMI. The associations were directionally consistent with MEA being inversely associated with behaviours including smoking and BMI. Our findings form a bridge between socio-economic factors and biology and highlight potential pathways underlying effects of maternal education. The results broaden our understanding of bio-social associations linked to differential DNA methylation in multiple early stages of life. The data generated also offers an important resource to help a more precise understanding of the social determinants of health.

## Introduction

Maternal educational attainment (MEA) is a multidimensional construct that influences child health and wellbeing via myriad social and biological pathways [1]. Among the core components of socio-economic position (SEP) *i.e*. employment, income, and education, MEA shows the strongest association with child neuro-cognitive development. It determines access to important resources, such as financial security, family circumstances, and material resources, that affect child birthweight, growth and development and cardio-metabolic health in later life [2].

MEA has been shown to influence other relevant intrauterine exposures such as nutrition, maternal smoking, body mass index etc. that are related to child health outcomes. Part of the downstream impact of intrauterine exposures on offspring health has been found to be through altered DNA methylation. Despite widespread recognition of social factors in health, prospective evidence for underlying mechanisms of this ‘*biological embedding*’ from an early time point is limited, and causal mechanisms are unknown. Recent research has revealed epigenetic variation associated with SEP and discovered that, when compared to other markers of SEP [3] education (either one’s own or one’s mother’s) has the largest influence. Similarly, only maternal education was related with four cytosine-phosphate-guanine (CpG) sites at birth and twenty in adolescence, according to a longitudinal analysis of 974 participants from the ALSPAC birth cohort (United Kingdom) [4]. With respect to own education, Linner et al. in a study including 10 767 participants from 27 cohorts within Social Science Genetics Association Consortium (SSGAC) identified nine CpGs related to educational attainment in adults aged 26.6–79.1 years, overlapping with findings from previous studies on adult smoking and maternal smoking during pregnancy [5].

**Fig. 1:**
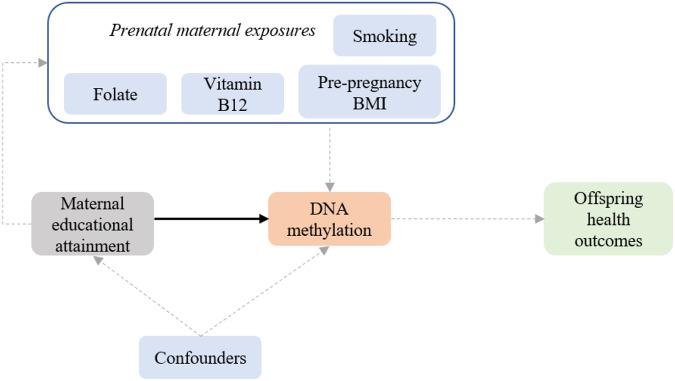
Conceptual framework showing association analysed in this study between maternal education attainment (MEA) in pregnancy and DNA methylation denoted by black arrow. Gray dotted arrows denote plausible measures that may be linked with MEA and DNA methylation.

A low level of maternal education is not a sufficient cause of offspring health per se, but it may mediate a vulnerability increasing the risk to be exposed to other prenatal exposures with direct effects on DNA methylation (Fig. 1). We aimed to quantify the associations of MEA with DNA methylation levels at birth, in childhood and in adolescence. Here we present meta-analyses of multiple EWASs in 37 studies from high income countries, with sample size of up-to 9881 individuals. We explored (i) if findings are enriched with those from EWASs of intrauterine exposures with clear impacts on offspring methylation, thereby indicating that MEA may serve as a proxy for better health behaviours; and (ii) association of implicated sites with gene expression in cells and tissues.

## Methods

### Participating cohorts

The study included 37 studies from high income countries in Europe, the USA and Australia within the Pregnancy And Childhood Epigenetics (PACE) Consortium [6]. The total sample across the three time points included 14,638 individuals comprising 96.3% European, 1.8% Hispanic, and 1.7% African ethnicity. Ethnicity was self-reported unless stated otherwise in the cohort specific methods (Supplementary File). DNA methylation was measured in offspring at three time points: birth (27 studies, *n* = 9 881), childhood (6 studies, *n* = 2 017), and adolescence (4 studies, *n* = 2 740). Participants had complete information on MEA, DNA methylation in cord blood or peripheral blood, and the covariates described below (complete case analysis). We excluded all twins and in case of non-twin siblings, one sibling was excluded by selecting based on completeness of data or, if equal, randomly.

Written informed consent was obtained for all participants, and studies were approved by the local ethics boards in accordance with the principles of the Declaration of Helsinki. Supplementary methods provide cohort-specific detailed information, and their ethics approval statements (Supplementary File).

### Maternal education measures

MEA at the time of pregnancy was defined in accordance with the International Standard Classification of Education (ISCED) 1997 classification (UNESCO) [7] and was harmonized across the cohorts. MEA was categorized into seven categories (coded 0 to 6) of educational attainment, which was then translated into years of schooling equivalents (0 to 22 years of schooling) as: Level 0 = 1 year, Level 1 = 7 years, Level 2 = 10 years, Level 3 = 13 years, Level 4 = 15 years, Level 5 = 19 years and Level 6 = 22 years of schooling (Supplementary Table 1).

### DNA methylation measurement

All cohorts extracted DNA from cord blood and/or peripheral blood samples. Samples were processed with the Infinium HumanMethylation450 or EPIC BeadChip assays [8]. Quality control and normalization were performed independently by the individual cohorts (Supplementary File). Untransformed beta-values were used as the outcome measure (DNA methylation beta-values 0-1). Methylation value outliers were excluded using the Tukey method: values < (25^th^ percentile- 3IQR) and values > (75^th^ percentile +3IQR) were removed [9]. CpGs located on the sex chromosomes were also removed.

### Covariates

The analysis included three models. In Model 1, associations were adjusted for sex, technical batch (cohort-specific variable) and estimated cell type proportions at birth, and additionally for child age in childhood and adolescence. The cell type proportions included CD8+ T-cells, CD4+ T-cells, natural killer cells, B cells, monocytes, granulocytes, and nucleated red blood cells at birth, estimated by using a cord blood-specific reference [10] and using the ‘Houseman method’ [11] using the Reinius reference set in peripheral blood [12]. Model 2 was additionally adjusted for maternal age, pre-pregnancy BMI, smoking (sustained smoking *vs* no smoking or stopping in early pregnancy), and gestational age at birth, to account for maternal prenatal factors. Model 3 additionally included offspring BMI and smoking (yes *vs* no). Models 1 and 2 were run at all three time points (birth, childhood, and adolescence) and model 3 in childhood and adolescence only, to account for offspring-specific covariates.

### Statistical analysis

#### Cohort-specific epigenome-wide association analyses

The flow chart of the study design is given in Supplementary File Fig. 1, and analyses were described in a pre-specified analysis plan (Supplementary File). Cohorts used a common script to perform independent epigenome-wide linear regression analyses with robust standard errors in R.

#### Meta-analysis

To minimize human error, researchers from two centres independently performed quality control of the cohort-level results and fixed-effects inverse-variance weighted meta-analyses and verified the results. Single cohort CpGs and 44,960 cross-reactive CpGs were removed [13, 14]. The final results included 429 959 (birth), 429 233 (childhood), and 427 349 (adolescence) CpGs. Multiple testing burden was accounted for using the method of Benjamini and Hochberg [15] and setting FDR to 5%. We also assessed CpGs associations with a more stringent Bonferroni correction (*P* < 1 × 10^−7^). The nearest gene for all CpGs were annotated based on the Illumina annotation file. We assessed inter-study heterogeneity by the *I*^*2*^ statistic, and constructed forest plots to visualize the results for CpGs with *I*^*2*^ > 50%.

#### Sensitivity analyses

To investigate the robustness of our findings, several sensitivity analyses were performed for model 1 results. First, we ran a leave-one-study-out analysis for the CpGs with *P*_*FDR*_ < 0.05 of each of the three age groups. Second, we re-ran the maternal meta-analyses for birth cohorts restricted to cohorts with participants of European ancestry only, which was the largest ancestry group (*n* = 9 501). Data in childhood and adolescence were only available for European ancestries. We examined overlap in the associated CpGs (*P*_*FDR*_ < 0.05) of the three meta-analyses at birth, childhood, and adolescence to explore temporal persistence of differential methylation.

#### Enrichment analyses

We examined whether CpGs with *I*^*2*^ < 50% were enriched for CpGs previously identified at FDR-significance in the meta-analyses of EWASs of maternal folate concentrations [16], vitamin B_12_ concentrations, smoking [17], and pre-pregnancy BMI [18] using a hypergeometric test.

#### Functional analyses

To assess potential mechanisms linking MEA to offspring DNA methylation, we explored associations with gene expression, by comparing the associated CpGs at birth (at *P*_*FDR*_ < 0.05) from model 1 with a catalogue containing 63 831 child-specific blood autosomal *cis*-expression quantitative trait methylation sites (*cis*-eQTMs, 1 Mb window) [19]. The GTEx gene-expression level of the identified nearest genes to the CpG sites were further assessed with the help of the webtool ‘Functional mapping and annotation of genetic associations’ (FUMA) [20]. We also explored whether the CpGs (*P*_*FDR*_ < 0.05) were enriched in DNase I hypersensitive sites, commonly associated with regulatory regions, using eFORGE v2.0. with its default settings [21].

## Results

### Descriptive statistics

Descriptive statistics for the 37 datasets are shown in Table 1. The meta-analysis sample included 49.2% females. The mean number of years of MEA at the time of pregnancy ranged from 12.3 to 19 years. Cohort-specific distributions of MEA are shown in Supplementary Table 1. Mean maternal age ranged from 27.4 to 33.8 years. Maternal smoking during pregnancy prevalence ranged from 2% to 48%. Mean maternal pre-pregnancy BMI ranged from 22.3 to 28.0 kg/m^2^ and mean gestational age at birth from 38.5 to 40.2 weeks.

**Table 1:** Population characteristics of all the participating cohort studies at birth, childhood, and adolescents.

Cohort acronyms^1^	Max. N^3^	Males, N (%)	Maternal Educational attainment during pregnancy (yrs), mean (SD)	Maternal age during pregnancy (yrs), mean (SD)	Maternal pre-pregnancy BMI (kg/m^2^), mean (SD)	Maternal smoking during pregnancy^4^, N (%)^3^	Gestational age at birth (wks), mean (SD)	Child age at the time of DNA measurement (yrs), mean (SD)	Child BMI age at the time of DNA measurement (kg/m^2^), mean (SD)	Country
ALSPAC_birth	782	409 (52.2)	14.9 (3.0)	29.7 (4.4)	22.8 (3.7)	81 (10.4)	39.6 (1.5)	na	na	UK
ALSPAC_childhood	846	443 (52.5)	14.9 (3.0)	29.8 (4.3)	22.8 (3.7)	86 (10.2)	39.6 (1.5)	7.5 (0.2)	16.2 (1.9)	UK
ALSPAC_adolescent	843	443 (52.5)	15.0 (3.1)	29.7 (4.4)	22.8 (3.7)	87 (10.4)	39.6 (1.5)	15.5 (0.3)	22.8 (3.5)	UK
CHS	247	147 (59.5)	14.2 (3.4)	na	na	19 (7.7)	na	na	na	USA
EAGeR	379	187 (50.0)	16.8 (2.6)	28.3 (4.4)	25.2 (5.6)	9 (2.4)	38.9 (1.5)	na	na	USA
EARLI	155	76 (49.7)	17.6 (3.5)	33.4 (4.6)	28.0 (7.3)	na	39.4 (1.3)	na	na	USA
EDEN_birth	159	95 (59.7)	14.7 (4.2)	30.2 (4.9)	23.5 (4.6)	26 (16.9)	39.5 (1.3)	na	na	France
EDEN_childhood	153	92 (59.7)	14.7 (4.2)	30.2 (4.9)	23.5 (4.6)	26 (16.9)	39.5 (1.3)	5.7 (0.1)	15.3 (1.5)	France
ENVIRONAGE	366	187 (51.1)	17.3 (3.7)	30.1 (4.3)	24.1 (4.4)	40 (10.9)	39.1 (1.6)	na	na	Belgium
EXPOSOMICS - ENVIRONAGE	190	90 (47.4)	16.2 (4.2)	29.4 (4.4)	24.0 (4.3)	25 (13.2)	39.1 (1.7)	na	na	Belgium
EXPOSOMICS - PICCOLIPIU	97	45 (46.4)	16.3 (3.9)	33.3 (4.5)	22.6 (3.9)	21 (21.7)	39.6 (1.6)	na	na	Italy
EXPOSOMICS - RHEA	92	41 (44.6)	13.9 (4.4)	29.9 (4.8)	23.1 (5.5)	18 (19.6)	38.5 (1.3)	na	na	Greece
FinnGedi	496	243 (48.9)	15.3 (3.0)	32.0 (5.2)	26.8 (5.6)	0	39.9 (1.3)	na	na	Finland
GECKO	242	113 (47.0)	13.9 (4.3)	30.8 (4.2)	24.4 (4.5)	117 (48.0)	40.2 (1.2)	na	na	Netherlands
GenR_birth	1361	685 (51.8)	17.7 (4.5)	31.7 (4.2)	22.3 (3.8)	184 (14.4)	40.1 (1.5)	na	na	Netherlands
GenR_childhood	483	245 (48.2)	18 (4.3)	32.2 (3.9)	23.2 (3.7)	44 (11.2)	40.2 (1.5)	6.0 (0.4)	15.9 (1.2)	Netherlands
Haven	138	73 (53.6)	15.3 (3.7)	31.5 (4.5)	24.8 (4.3)	15 (10.9)	39.8 (1.9)	na	na	Netherlands
Healthy start cohort – Non Hispanic White	259	137 (52.9)	18.2 (3.9)	29.6 (5.2)	24.42 (5.50)	22 (8.0)	39.6 (1.2)	na	na	USA
Healthy start cohort – Hispanic^2^	132	67 (50.7)	13.3 (3.6)	24.6 (5.7)	28.61 (8.31)	11 (8.0)	39.4 (1.2)	na	na	
Healthy start cohort - African American^2^	84	45 (53.6)	13.3 (3.5)	23.3 (5.9)	26.81 (7.55)	14 (17.0)	39.2 (1.4)	na	na	
INMA_birth	377	196 (52.2)	14.3 (4.0)	30.3 (4.1)	23.8 (4.4)	55 (14.5)	39.8 (1.4)	na	na	Spain
INMA_childhood	197	104 (52.8)	14.4 (4.0)	30.5 (4.2)	24.4 (5.0)	23 (11.6)	39.9 (1.3)	4.4 (0.2)	16.1 (1.5)	Spain
LINA	472	226 (48.0)	15.6 (3.0)	30.6 (4.5)	na	22 (4.7)	39.8 (1.5)	na	na	Germany
MARBLES	276	145 (58.9)	17.3 (3.2)	34.3 (4.7)	27.9 (7.3)	na	39.1 (1.2)	na	na	Spain
MOBA 1	984	458 (46.7)	17.4 (2.5)	29.9 (4.4)	24.1 (4.2)	147 (14.9)	39.5 (1.6)	na	na	Norway
MOBA 2	632	274 (43.4)	17.3 (2.6)	29.9 (4.5)	24.3 (4.5)	75 (11.9)	39.5 (1.6)	na	na	Norway
MOBA 3	216	105 (49.6)	17.3 (2.8)	29.5 (4.5)	24.0 (4.0)	na	39.7 (1.5)	na	na	Norway
NEST-African^2^	164	79 (48.2)	14.1 (2.3)	27.4 (6.2)	31.4 (10.4)	35 (21.3)	38.4(2.1)	na	na	USA
NEST-Caucasian	163	81 (49.7)	16.8 (2.9)	30.7 (5.8)	25.2 (7.2)	31 (19.0)	38.7 (2.1)	na	na	
NFBC1986	490	304 (53.2)	12.9 (2.9)	27.9 (5.3)	22.3 (3.3)	48 (9.8)	40.2 (1.1)	16.0 (0.4)	21.5 (3.5)	Finland
POGO	49	23 (46.9)	15.3 (4.3)	33.8 (5.0)	26.8 (7.3)	9 (2.0)	39.5 (1.2)	7.3 (2.4)	16.3 (2.4)	Germany
POSEIDON	295	154 (52.2)	16.3 (3.7)	31.4 (4.8)	24.8 (5.4)	32 (10.9)	39.2 (1.3)	na	na	Germany
PREDO	780	413 (52.9)	15.2 (2.8)	33.4 (5.6)	27.4 (6.5)	31 (3.9)	39.8 (1.6)	na	na	Finland
Raine	995	494 (49.6)	12.3 (3.5)	28.5 (5.9)	22.5 (4.5)	197 (19.8)	39.3 (2.1)	17.1 (0.3)	23.3 (4.5)	Australia
STOPPA	412	208 (51.0)	15.9 (2.9)	31.8 (4.7)	24.2 (4.0)	0	36.9 (2.6)	12.5 (1.4)	18.7 (2.8)	Sweden
Project Viva_birth	344	169 (49.3)	19.0 (2.2)	33.1 (4.5)	24.3 (4.9)	31 (9.0)	39.8 (1.5)	na	na	USA
Project Viva_childhood	289	140 (48.4)	18.4 (2.1)	33.3 (4.5)	24.4 (4.7)	27 (9.3)	39.7 (1.5)	7.8 (0.7)	16.6 (2.4)	USA

^1^Cohort full names: The Avon Longitudinal Study of Parents and Children (ALSPAC) (specifically subset with DNA methylation profiles in the Accessible Resource for Integrated Epigenomic Studies, ARIES), The Children’s Health Study (CHS); Effect of Aspirin in Gestation and Reproduction (EAGeR); Early Autism Risk Longitudinal Investigation cohort (EARLI); Markers of Autism Risk Learning Early Signs (MARBLES); Etude des Déterminants pré et post natals du développement et de la santé de l′Enfant (EDEN); ENVIRonmental influence ON early AGEing (ENVIRONAGE); Finnish Gestational Diabetes (FinnGeDi); Groningen Expert Center for Kids with Obesity (GECKO); Generation R Study (GenR); Heart anomalies and the role of genetic and nutritional factors (HAVEN), INfancia y Medio Ambiente (INMA) – Sabadell; Lifestyle and environmental factors and their Influence on Newborns Allergy risk (LINA); The Norwegian Mother, Father and Child Cohort Study (MoBa); Newborn Epigenetics STudy (NEST); Northern Finland Birth cohort 1986 (NFBC1986), Postpartum Outcomes in mothers with Gestational diabetes and their Offspring (POGO)**;** Pre-, Peri-, and Postnatal Stress: Epigenetic impact on Depression (POSEIDON); The Western Australian Pregnancy Cohort study (Raine), The Swedish Twin study On Prediction and Prevention of Asthma (STOPPA)

^2^Cohorts with non-European ancestry.

*na* not available, *SD* Standard Deviation, *BMI* Body Mass Index, *EA* Education attainment.

^3^Sample size of the studies with DNA methylation data.

^4^Smoking was defined as any maternal smoking during pregnancy.

### Meta-analyses of epigenome-wide association studies

**Fig. 2:**
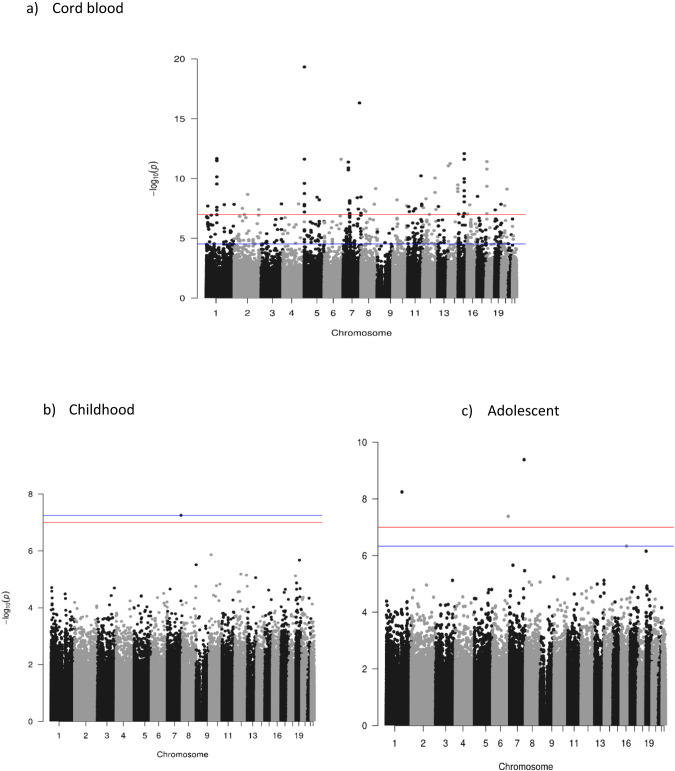
Manhattan plots of the maternal education attainment EWAS model 1 in the offspring at three time points. The x axis is the chromosomal position, and the y axis is the *P*-value on a -log_10_ scale. The blue line corresponds to the first CpG site for which *P*_*FDR*_ < 0.05 and red line indicates suggestive significance *P* = 1 × 10^−7^. The Manhattan plot of the fully adjusted models are presented in Supplementary Fig. 2.

Genomic inflation factors (λ_gc_) for the models are shown in Supplementary Table 2. Figure 2 shows the Manhattan plots of model 1 at the three time points and Table 2 shows the top 20 significant hits (*P*_*FDR*_ < 0.05) at birth and all hits for childhood and in adolescence. QQ plots of all the meta-analysis Manhattan plots for models 2 and 3 are reported in Supplementary File, Figs. 2 and 3. In model 1, MEA was associated with DNA methylation at 473 CpGs at birth, one CpG in childhood and four CpGs in adolescence at *P*_*FDR*_ < 0.05 (Fig. 2, Table 2, and Supplementary Table 2 and 3). Using a more stringent Bonferroni-corrected *p*-value cut-off of *P* < 1 × 10^−7^, 182 CpGs at birth were associated, as well as all CpGs in childhood and in adolescence. cg25949550 (*CNTNAP2)* was the only CpG associated with MEA at all three time points. For each year increase in MEA, DNA methylation was higher by 0.05% (SE = 0.006, *P* ≤ 3.5 x 10^−8^, *I*^*2*^ = 41.5) at birth, 0.06% (SE = 0.006, *P* ≤ 7.6x10^−8^, *I*^*2*^ = 1.1) in childhood, and 0.08% (SE = 0.006, *P* ≤ 4.1 × 10^−10^, *I*^*2*^ = 37.7) in adolescence.

**Table 2:** Epigenome-wide associations of maternal educational attainment in the offspring from model 1 of top 20 CpG’s at the birth and all the CpGs at childhood and adolescence.

CpG	Chr	BETA	SE	*P*	*N*	I^2^	Nearest gene
**Birth**							
cg05575921	5	0.0010	0.0001	4.70E-20	8903	56.1	*AHRR*
cg25949550	7	0.0005	6.0E-05	4.81E-17	8920	41.5	*CNTNAP2*
cg12101586	15	−0.0013	0.0002	8.21E-13	8922	51.8	*CYP1A1*
cg06338710	1	0.0009	0.0001	2.14E-12	8147	54.7	*GFI1*
cg11902777	5	0.0004	5.3E-05	2.39E-12	8903	0	*AHRR*
cg15971980	6	−0.0009	0.0001	2.43E-12	8926	0	
cg05549655	15	−0.0009	0.0001	2.46E-12	8926	41.9	*CYP1A1*
cg09935388	1	0.0021	0.0003	3.20E-12	8910	62.6	*GFI1*
cg27093273	18	−0.0013	0.0002	3.82E-12	8549	0	*TMEM200C*
cg12803068	7	−0.0017	0.0003	4.17E-12	8680	48.0	*MYO1G*
cg19474546	14	−0.0012	0.0002	5.44E-12	8657	54.0	
cg05282518	14	−0.0013	0.0002	8.50E-12	8548	40.1	*OR4K2*
cg22132788	7	−0.0007	0.0001	1.28E-11	7483	50.1	*MYO1G*
cg26438105	18	−0.0013	0.0002	1.63E-11	8549	0	*TMEM200C*
cg04180046	7	−0.0012	0.0002	1.93E-11	8926	47.7	*MYO1G*
cg10287786	11	0.0012	0.0002	6.07E-11	8928	30.2	*DSCAML1*
cg12876356	1	0.0018	0.0003	7.27E-11	8876	53.9	*GFI1*
cg06892868	12	−0.0009	0.0001	9.03E-11	8927	42.5	*MGC14436*
cg18092474	15	−0.0014	0.0002	1.03E-10	8926	36.4	*CYP1A1*
cg22549041	15	−0.0013	0.0002	2.02E-10	8909	55.5	*CYP1A1*
**Childhood**							
cg25949550	7	0.0006	0.0001	5.60E-08	2015	1.1	*CNTNAP2*
**Adolescence**							
cg25949550	7	0.0008	0.0001	4.13E-10	2484	0	*CNTNAP2*
cg13246497	1	−0.0014	0.0002	5.70E-09	2485	0	
cg25376310	6	0.0010	0.0002	4.16E-08	2487	37.7	*ZDHHC14*
cg00253658	16	−0.0019	0.0004	4.64E-07	2479	0	

DNA methylation beta values can be interpreted as unit change in methylation level for one year increase in MEA. *CpG* Cytosine Phosphate Guanine, *MEA* maternal educational attainment, *SE* Standard Error.

In the fully adjusted model 2 and 3 (Supplementary Table 4, 5 and Supplementary File, Fig. 2), MEA was associated with DNA methylation at two CpGs at birth, two in childhood and three in adolescence at *P*_*FDR*_ < 0.05. These overlapped with CpGs found in model 1. Using a Bonferroni-corrected *p*-value cut-off of *P* < 1 × 10^−7^, DNA methylation at one CpG remained associated at birth, two in childhood and three in adolescence. Twenty-four CpGs had *I*^*2*^ > 50 at birth.

### Sensitivity meta-analyses

The leave-one-out analyses on the 24 CpGs with *I*^*2*^ > 50 at birth showed for some of these (e.g., cg01952185, cg05383657), the meta-analysis results were influenced by the Generation R Study. However, removing this study resulted in larger absolute effect sizes, therefore any potential influence of Generation R would be towards the null (Supplementary File, Figures 4,5). Findings were consistent with our results at birth when only studies of European ethnicity were assessed (*r* = 0.97) (Supplementary Table 6).

### Enrichment analysis

At birth, we observed enrichment (*P* < 1 × 10^-5^) for findings from previous EWASs of other prenatal exposures, namely maternal folate, vitamin B_12_ concentrations, smoking and pre-pregnancy BMI (*P*_*enrichment*_< range = 1.9 x 10^−04^ to 2.4 x 10^−138^) (Table 3). The directions of the effects were concordant for all the overlapping CpGs for maternal folate and vitamin B_12_ concentrations and were in the expected opposite direction for all the overlapping CpGs between MEA and maternal smoking (except for cg23989336, which was in the same direction) and pre-pregnancy BMI. For childhood and adolescence there was enrichment only for maternal smoking during pregnancy (*P*_*enrichment*_ < 0.02 and 0.001).

**Table 3:** Enrichment for maternal educational attainment related CpG’s in the offspring at birth with DNA methylation signatures of maternal prenatal exposures.

		Birth		Childhood		Adolescents	
Maternal prenatal exposures	*N*	Overlap *n*	Overlap *P*-value	Overlap n	Overlap *P*-value	Overlap *n*	Overlap *P*-value
Maternal folate	443	74	2.41E–138	0	–	0	–
Maternal Vit B12	109	22	1.11E–43	0	–	0	–
Maternal smoking	6073	85	1.06E–66	1	0.01	2	0.001
Maternal BMI during pregnancy	104	3	1.97E–04	0	–	0	–

### Functional analyses

Using the CpGs suggestively associated with MEA at birth (at *P* < 1 × 10^−5^) from model 1, we found 89 unique CpG-gene expression pairs (*cis*-eQTMs) (*P* < 1 × 10^−5^) in an eQTM atlas based on blood samples collected in childhood (6–11 years). These *cis-*eQTMs involved 74 unique CpGs and 68 unique transcript clusters, which can be interpreted as putative genes (Supplementary Table 7). Increased DNA methylation was associated with decreased expression in 43 of these eQTMs, with increased expression in 46. Seventeen CpGs were associated with expression of *HOTAIRM1* and six CpGs with expression of *FRG1BP*. We further assessed the tissue expression related to the genes of the identified 68 unique transcript clusters using GTEx gene-expression level in FUMA. The genes were found to be expressed across multiple tissues; however, multiple clusters of genes were observed in the brain and heart tissues (Supplementary File, Fig. 6). Using eFORGE (at *P* < 1 × 10^−5^) we found enrichment of DNAase I hypersensitive sites and of specific transcription factor motifs in adolescent blood (Supplementary File, Fig. 7).

## Discussion

Our well-powered meta-analysis combining results from 37 studies from high income countries showed that MEA is associated with DNA methylation in the offspring at birth, in childhood, and in adolescence. Robust associations with MEA were found for 473 CpG sites at birth, one in childhood, and four in adolescence. At all ages, there was enrichment for findings from previous EWAS on maternal folate concentrations, vitamin B_12_ concentrations, smoking, and pre-pregnancy BMI.

### Meta-analysis

DNA methylation at cg25949550 was consistently positively associated with MEA across all models and time points. A 1-year increase in MEA was associated with an increase of 0.05–0.08% in blood DNA methylation at cg25949550. This CpG is located at intron 1 of *CNTNAP2*, and overlaps with binding sites of transcription repressors SIN3A, CTBP2, CTCF and REST. *CNTNAP2* genetic variations have been implicated in multiple neurodevelopmental disorders including schizophrenia, epilepsy, autism spectrum disorder, attention-deficit/hyperactivity disorder, and mental retardation [22]. Notably, in our study the associations of cg25949550 with MEA in pregnancy after adjusting for sustained maternal smoking during pregnancy disappeared in birth and childhood studies but remained in adolescents. DNA methylation at cg25949550 has been repeatedly found to be strongly associated with maternal smoking during pregnancy as well as with personal smoking in adults [23]. Among the MEA related CpG sites at birth, four CpGs, cg05575921 (*AHRR*), cg12803068 (*MYO1G*), cg22132788 (*MYO1G*) and cg21161138 (*AHRR*) overlapped with the findings from a previous large EWAS on own educational attainment by Linner et al. among adults. All these CpGs are strongly related to personal smoking and cg05575921 is one of the top CpGs related to smoking, was the strongest associated CpG site in both studies (*P* < 1 × 10^−17^). Linner et al. also found that all nine CpGs associated with the participant’s own educational attainment overlapped with those from the EWAS of maternal smoking, which is concordant with our study. Similarly, Van Dongen et al. identified that educational attainment CpGs overlapped with smoking signatures in a meta-analysis of four cohorts [24].

Consistent with previous studies assessing associations of socio-economic status with DNA methylation [4, 25], MEA associated CpGs at birth persisted only minimally in childhood and adolescence. Persistence of differential DNA methylation in offspring may not be a pre-requisite for long-term impacts of MEA on offspring health, as transient differential DNA methylation *in utero* can cause lasting functional changes predisposing offspring to later adverse outcomes [26–28]. We observed attenuation in the associations (models 2 and model 3) after adjusting for prenatal covariates such as maternal BMI, smoking, age, and gestational age. This was expected and emphasizes that the *in-utero* environment represents the combined effect of multiple prenatal factors. We are aware that there are other covariates that we were unable to adjust for in this study and which may affect the identified associations. However, we believe the covariates used were representative of several important aspects of the social dynamics of health.

### Enrichment analysis

In the enrichment analysis, we observed that 85 of 473 CpGs overlapped with CpGs identified in relation to maternal smoking during pregnancy and had the expected opposite direction of effect for all the CpGs (except for cg23989336). Maternal smoking has repeatedly been found to be negatively associated with educational attainment: mothers with lower education are more likely to continue smoking in pregnancy compared to mothers with higher education [29]. A systematic review of 63 studies using Mendelian randomization identified robust evidence that higher educational attainment decreases smoking [30]. Gilman et al. evaluated a potential causal effect of educational attainment on smoking and observed that adjusting for a wide range of social factors had little impact on the association between the two [31]. It is therefore likely that smoking is rather a consequence, acting as mediator between educational attainment and health outcomes. Similarly, we observed overlap of sites associated with MEA with other prenatal exposures involved in *in-utero* programming such as maternal folate and vitamin B12 concentrations (with concordant directions of effect), and maternal pre-pregnancy BMI. The overlap of CpGs between MEA and these prenatal exposures may indicate a shared social molecular architecture between them leading to common biosocial pathways that may influence health outcomes, as often observed in observational studies.

### Functional analyses

We found *cis*-eQTM involving the *HOTAIRM1* and *FRG1BP* genes. Genetic variations in *HOTAIRM1* are known to be involved in the neuronal differentiation and associated with waist-to-hip ratio phenotype. CpGs annotated to this gene were differentially methylated in newborns in relation to sustained maternal smoking during pregnancy and to own smoking in adults [23]. The *HOTAIRM1*gene also epigenetically controls the expression of the proneural transcription factor NEUROGENIN 2 that is critical for brain development [32]. *FRG1BP* (previously known as *C20orf80*, *FRG1B*) has been found to be associated with body weight, body height at birth and ocular sarcoidosis phenotypes [33]. Furthermore, we found enrichment of DNAase I hypersensitive sites and of specific transcription factor motifs in adolescents at RAR, ESRRA, V LXR and CTCF (Supplementary File, Fig. 7) regulating genes which play roles in cell differentiation, proliferation, have neuroprotective actions and regulate cholesterol metabolism, inflammation, autoimmunity, and cancer [34, 35]. Overall, these findings from our gene expression and tissue specific enrichment may indicate a role of MEA in important biological processes and pathways of the offspring, aligning with their multifaceted role observed in epidemiological studies.

Due to the multidimensionality of MEA, it has remained a challenge for researchers to disentangle the interrelationships with its close correlates including income, employment, and socio-economic status. These measures reflect different types of resources that may differentially impact a child’s biological development. Furthermore, our measure of educational attainment is unable to capture differences in educational quality, type, or other institutional or systemic factors that might independently influence biological health. It also focuses on individual-level aspects of education, leaving out the social context in which the education and health processes are embedded [36]. This raises several questions regarding the biological processes underlying these associations and our study should be seen as a steppingstone in this regard. Our findings likely represent a myriad of pathways related to MEA including adverse intrauterine (such as nutrition or toxicants), as well as childhood and adolescent exposures; thus, it is plausible that MEA is an upstream risk factor for proximal health behaviours. More research is warranted to understand the causality, to examine these associations in more ethnically diverse cohorts, and to study these associations in larger samples at later ages to gain in-depth insight into life-course trajectories.

### Strengths and limitations

The main strength of this study is that it uses a large sample size and three critical time points of human development from birth up to adolescence. We harmonized MEA to promote comparability of results across all cohorts. The summary statistics from our study should be a useful resource for future studies to further examine the interplay of various social factors and their associations with numerous biological pathways. MEA captures various biosocial dimensions of health as highlighted by our enrichment analyses, and our examination of potentially related factors, such as maternal smoking, provides a platform for future studies to disentangle potential causal relationships.

Our findings should also be interpreted in the light of certain limitations. The participants in our study were relatively well-educated and from high income countries and thus, our findings may not be generalizable to disadvantaged populations that are more vulnerable to adverse health outcomes. This study included mostly individuals of European ancestry and a small sample from African and Hispanic backgrounds due to lack of data availability; hence, the findings are not generalizable to ancestries beyond Europeans. We assessed MEA at the time of pregnancy and did not investigate education attained later in the childhood and adolescent cohorts. It is important to emphasize that we did not aim to draw direct causal conclusions, or to distinguish how much of these associations were confounded by other factors such as paternal education to understand the importance of maternal factors in the context of the family on DNA methylation [37]. Importantly, we observed overlap of methylation sites between maternal smoking and education, and the adjustment for sustained maternal smoking attenuated the associations at birth. It is likely that among individuals who continue to smoke throughout pregnancy, those of lower educational status might be over-represented.

We found that MEA at the time of pregnancy was associated with offspring DNA methylation at birth, in childhood, and in adolescence. The findings from the gene expression and enrichment analyses identified differential DNA methylation of genes involved in important biological processes. This may mean that socio-economic factors such as maternal education leave a “biological residue” which in turn may influence development, health, and wellbeing. Given the known association between higher maternal educational attainment and unhealthy maternal conditions (for ex. increased BMI, history of smoking, low folate levels, low Vitamin B12) [38] that have been linked to differences in DNA methylation patterns, investing in education access, especially in low-resource settings, holds potential to reduce health inequalities and improve the well-being across generations. This is consistent with the hypothesis that public health benefits are gained by improving educational attainment and addressing the social determinants of health [36, 39]. The summary statistics from this study provide an important resource for future studies to further investigate the intricate biosocial pathways involved in in-utero programming and establish a more comprehensive understanding of intergenerational health.

### Disclaimer

Where authors are identified as personnel of the International Agency for Research on Cancer/ World Health Organization, the authors alone are responsible for the views expressed in this article and they do not necessarily represent the decisions, policy, or views of the International Agency for Research on Cancer/ World Health Organization.

### Supplementary information

Supplementary file

Supplementary tables 1–7

## Data Availability

Meta-analysis results files will be deposited in the EWAS Catalogue data repository (http://ewascatalog.org) upon publication.
