# Supplementary material for: Maternal educational attainment in pregnancy and epigenome-wide DNA methylation changes in the offspring from birth until adolescence
Source: Mol Psychiatry. 2023 Dec 5;29(2):348–58. doi: 10.1038/s41380-023-02331-5 (PMC11116099; doi:10.1038/s41380-023-02331-5)
Supplement: Supplementary file 1 — Supplementary file [file 41380_2023_2331_MOESM1_ESM.docx]

**Table of Contents**

[**Figure 1**. Summary of study design 3](#_Toc137633029)

[**Figure 2**. Manhattan plot of the fully adjusted maternal educational attainment (MEA) EWAS model in the offspring in cord blood (model 2), childhood (model 3), and adolescence (model 3). The x axis is the chromosomal position, and the y axis is the P-value on a -log10 scale. The blue line corresponds to the first CpG site for which *P_FDR_* < 0.05 and red line marks the threshold for epigenome-wide significance *P*=1x10^-7^. The blue line is plotted at the first CpG with PFDR<0.05, this line can be higher than the red if there is the same number of CpGs with PFDR<0.05 and P<1x10^-7^ 4](#_Toc137633030)

[**Figure 3**. QQ plot and lambda values of the meta-analyses of maternal educational attainment (MEA) in cord blood, in childhood and in adolescence in all the models 6](#_Toc137633031)

[**Figure 4.** Leave-one-out analysis (systematically leaving one cohort at a time out of the meta-analysis) for the main model, for 24 CpGs with I^2^>50%. The red bars represent the effect estimate and 95% confidence interval of the meta-analysis when leaving out the indicated cohort. 7](#_Toc137633032)

[**Figure 5.** Leave-one-out analysis (systematically leaving one CpG at a time out of the meta-analysis) for the main model, for 24 CpGs with I^2^>50%. 11](#_Toc137633033)

[**Figure 6**. Heatmap of the expression analysis using GTEx expression 17](#_Toc137633034)

[**Figure 7**. Tissue specific enrichment from eForge analysis in the adolescent analysis 18](#_Toc137633035)

[**Cohort-specific methods** 19](#_Toc137633036)

[Avon Longitudinal Study of Parents and Children (ALSPAC) 19](#_Toc137633037)

[Children’s Health Study (CHS) 20](#_Toc137633038)

[Effect of Aspirin in Gestation and Reproduction (EAGeR) 21](#_Toc137633039)

[Early Autism Risk Longitudinal Investigation cohort (EARLI) 22](#_Toc137633040)

[Markers of Autism Risk Learning Early Signs (MARBLES) 23](#_Toc137633041)

[EDEN 24](#_Toc137633042)

[ENVIRonmental influence ON early AGEing (ENVIRONAGE) 25](#_Toc137633043)

[EXPOsOMICS (ENVIRONAGE + RHEA + PICCOLIPIU) 26](#_Toc137633044)

[Finnish Gestational Diabetes (FinnGeDi) study 29](#_Toc137633045)

[Groningen Expert Center for Kids with Obesity (GECKO) 30](#_Toc137633046)

[Generation R Study (GenR) 31](#_Toc137633047)

[HAVEN 32](#_Toc137633048)

[The Healthy Start Study 33](#_Toc137633049)

[INfancia y Medio Ambiente (INMA) - Sabadell 34](#_Toc137633050)

[Lifestyle and environmental factors and their Influence on Newborns Allergy risk (LINA) 36](#_Toc137633051)

[The Norwegian Mother, Father and Child Cohort Study (MoBa) 36](#_Toc137633052)

[Newborn Epigenetics STudy (NEST) 38](#_Toc137633053)

[The Northern Finland Birth Cohort (NFBC1986) 39](#_Toc137633054)

[Postpartum Outcomes in mothers with Gestational diabetes and their Offspring (POGO 40](#_Toc137633055)

[Pre-, Peri-, and Postnatal Stress: Epigenetic impact on Depression (POSEIDON) 42](#_Toc137633056)

[The Prediction and Prevention of Preeclampsia and Intrauterine Growth Restriction (PREDO) 43](#_Toc137633057)

[The Raine Study 44](#_Toc137633058)

[The Swedish Twin study On Prediction and Prevention of Asthma (STOPPA) 45](#_Toc137633059)

[Project Viva 46](#_Toc137633060)

[**Cohort-specific acknowledgements** 47](#_Toc137633061)

[**Cohort-specific funding statements** 49](#_Toc137633062)

[**References** 53](#_Toc137633063)

[**Analytical plan** 57](#_Toc137633064)


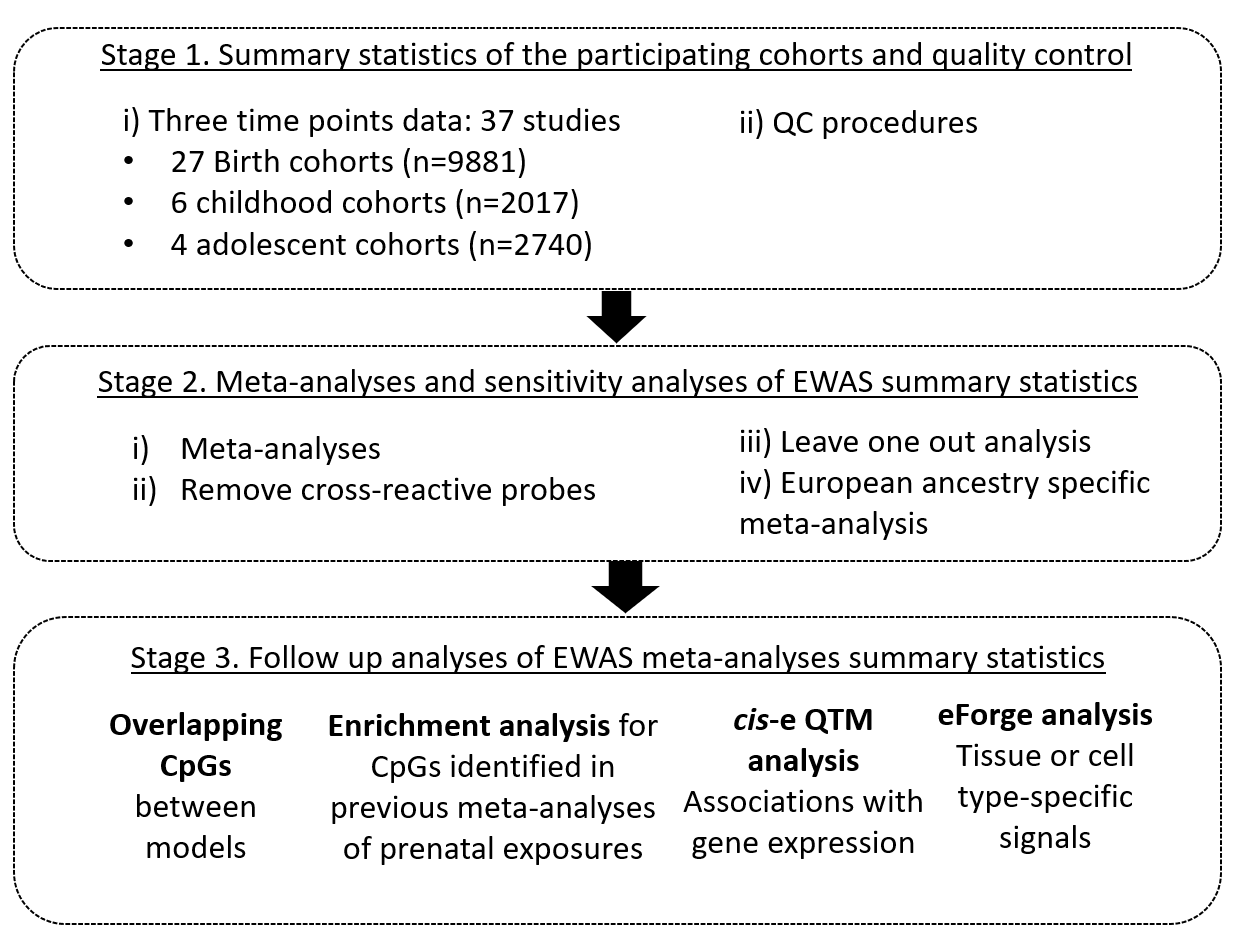


# **Figure 1**. Summary of study design

1. Cord blood


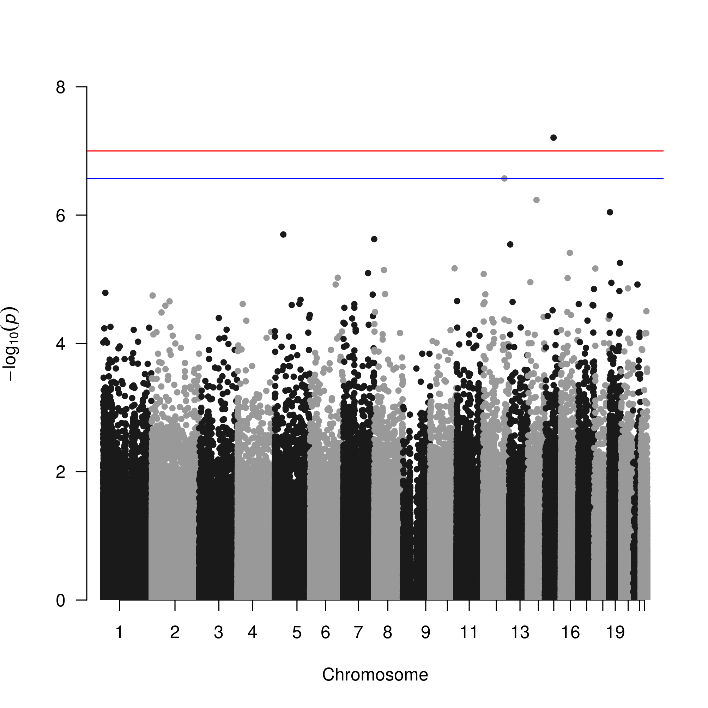


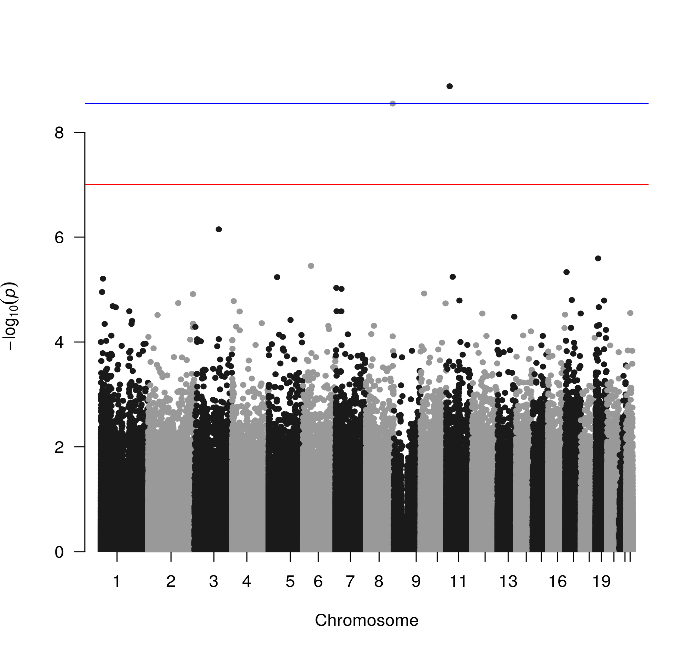

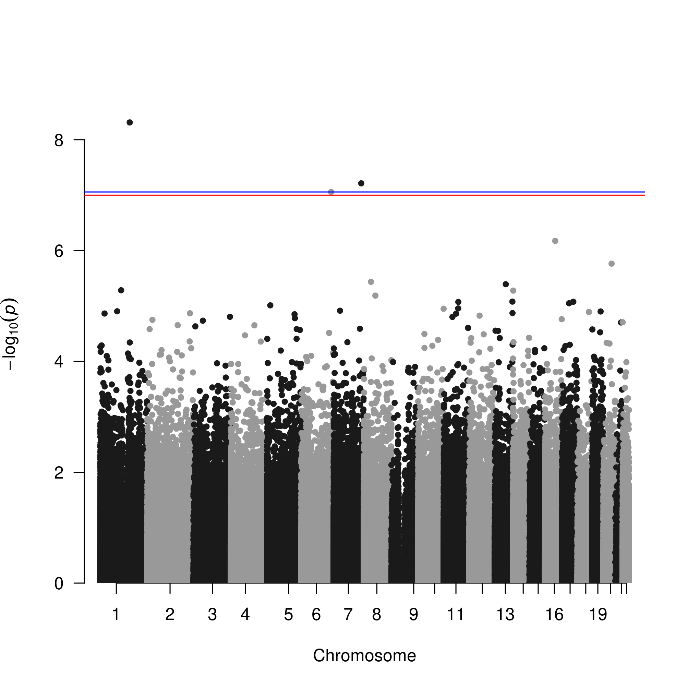


b) Childhood

c) Adolescent

# **Figure 2**. Manhattan plot of the fully adjusted maternal educational attainment (MEA) EWAS model in the offspring in cord blood (model 2), childhood (model 3), and adolescence (model 3). The x axis is the chromosomal position, and the y axis is the P-value on a -log10 scale. The blue line corresponds to the first CpG site for which *P_FDR_* < 0.05 and red line marks the threshold for epigenome-wide significance *P*=1x10^-7^. The blue line is plotted at the first CpG with PFDR<0.05, this line can be higher than the red if there is the same number of CpGs with PFDR<0.05 and P<1x10^-7^


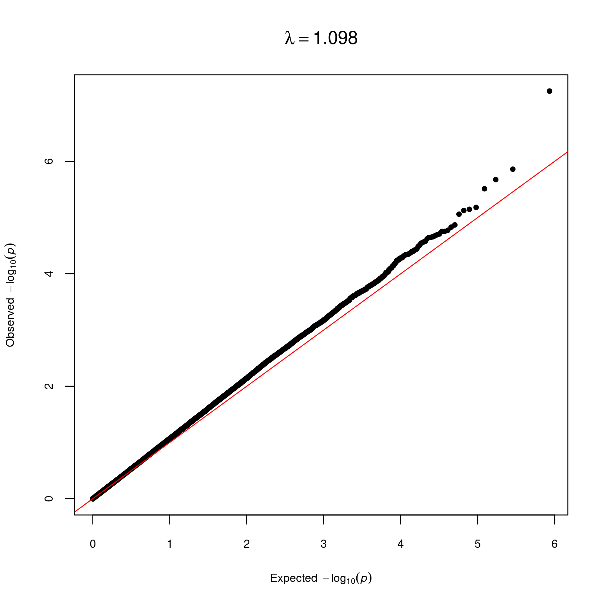

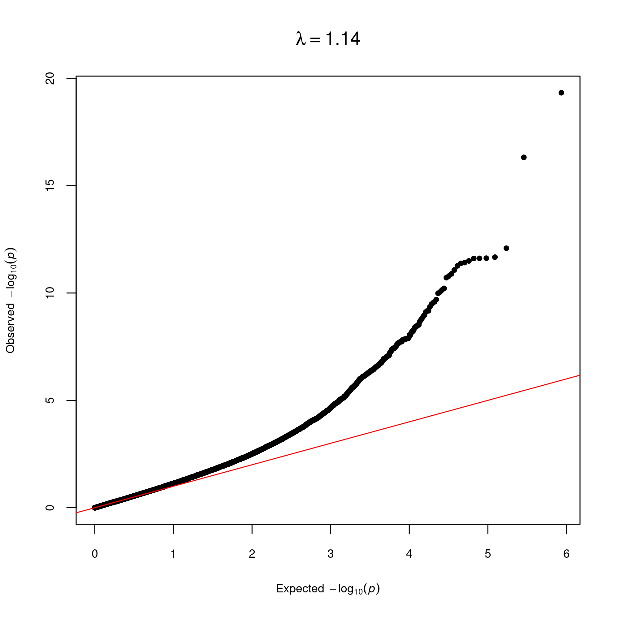


Model 1: Childhood

Model 1: Cord blood

Model 1: Adolescence


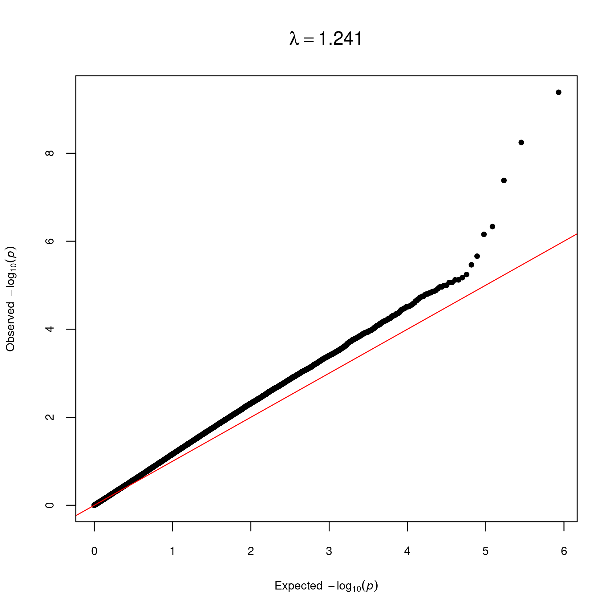


Model 2: Childhood

Model 2: Cord blood


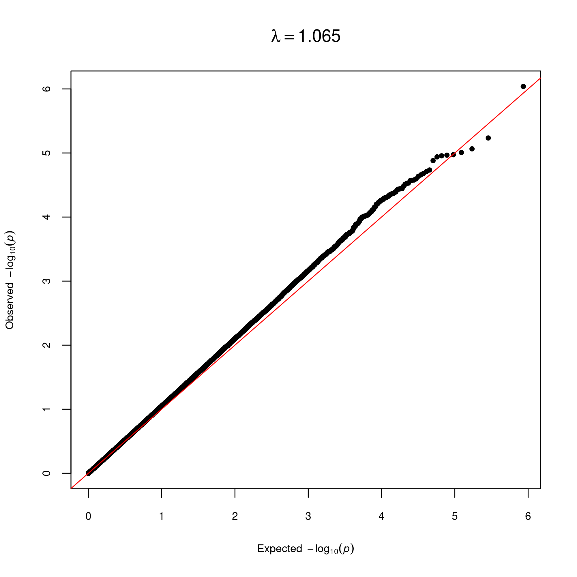

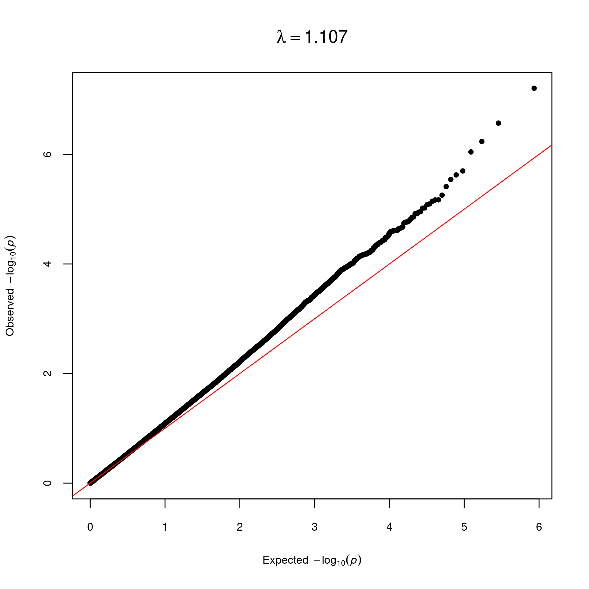


Model 2: Adolescence


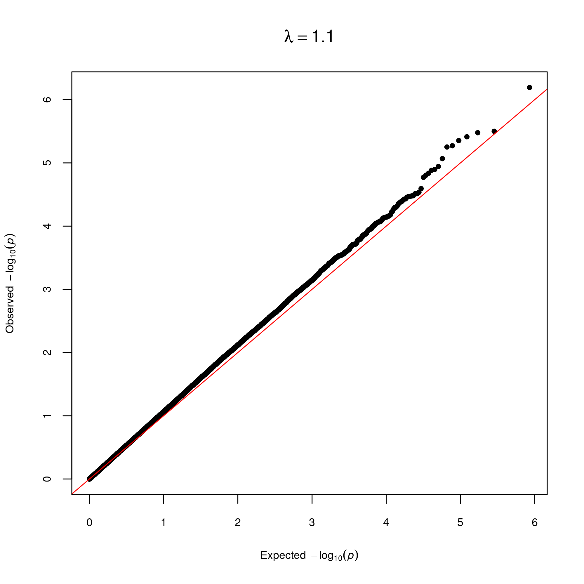


Model 3: Childhood

Model 3: Adolescence


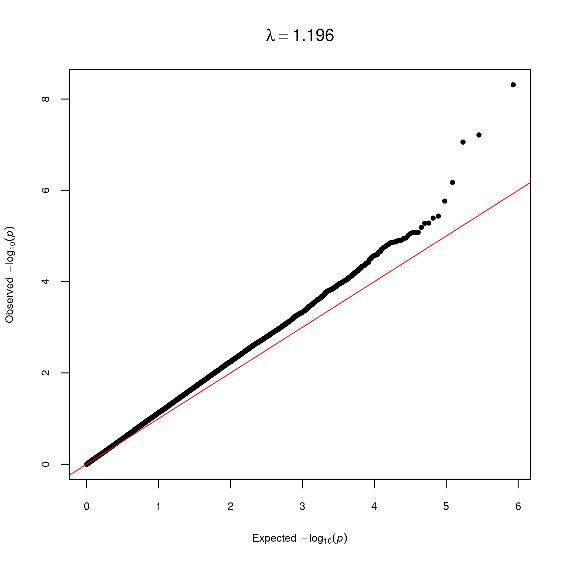

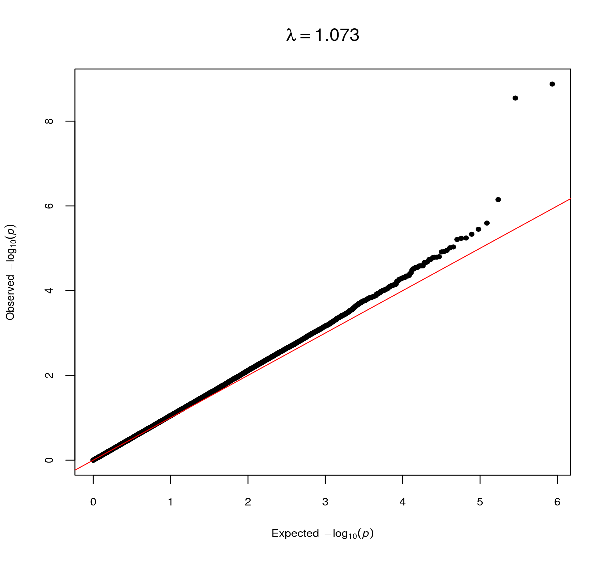


#

# **Figure 3**. QQ plot and lambda values of the meta-analyses of maternal educational attainment (MEA) in cord blood, in childhood and in adolescence in all the models

# **Figure 4.** Leave-one-out analysis (systematically leaving one cohort at a time out of the meta-analysis) for the main model, for 24 CpGs with I^2^>50%. The red bars represent the effect estimate and 95% confidence interval of the meta-analysis when leaving out the indicated cohort.


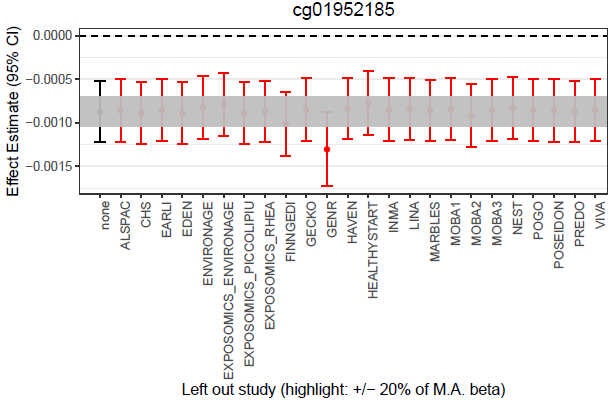

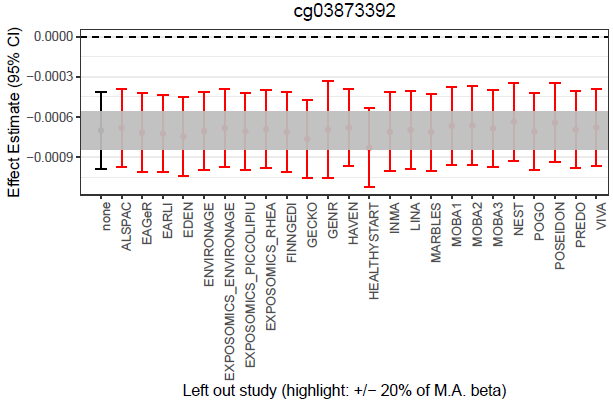


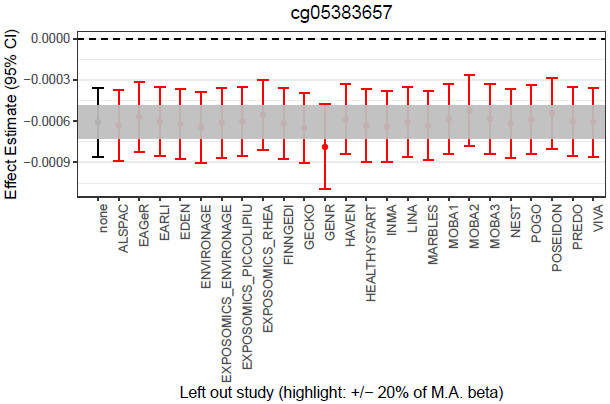

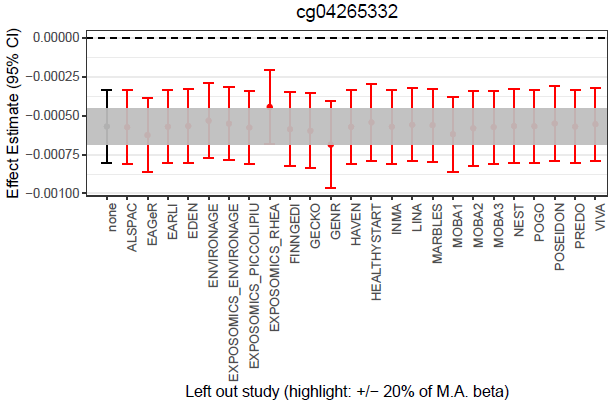


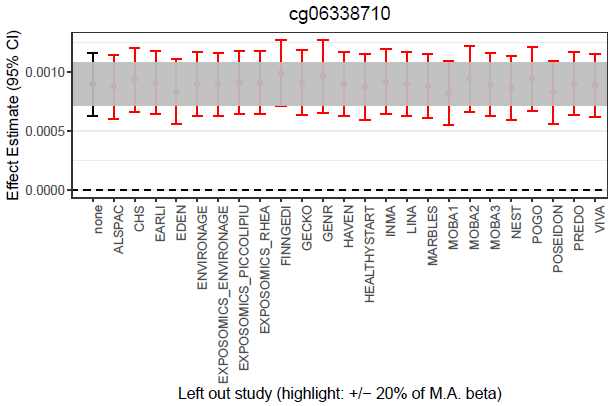

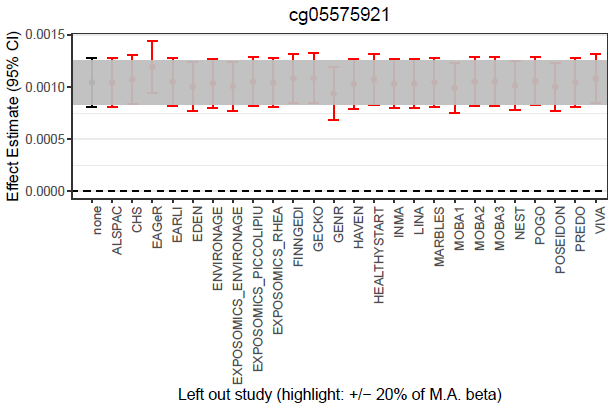


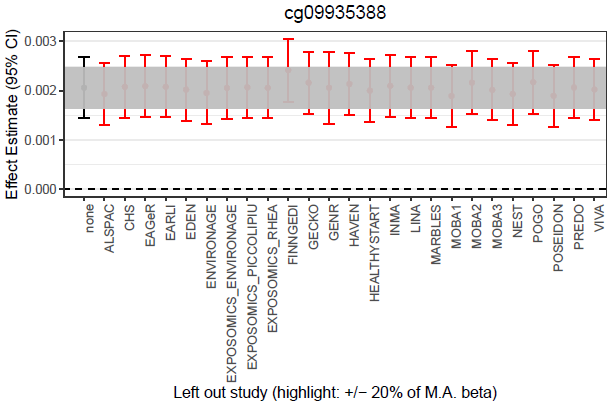

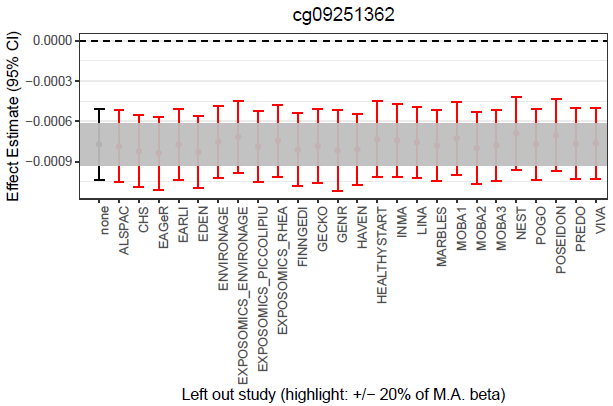


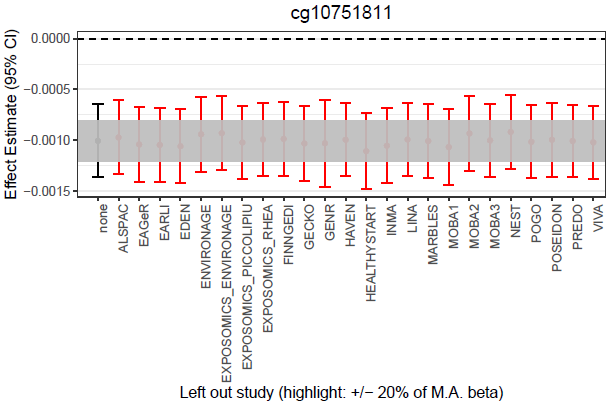

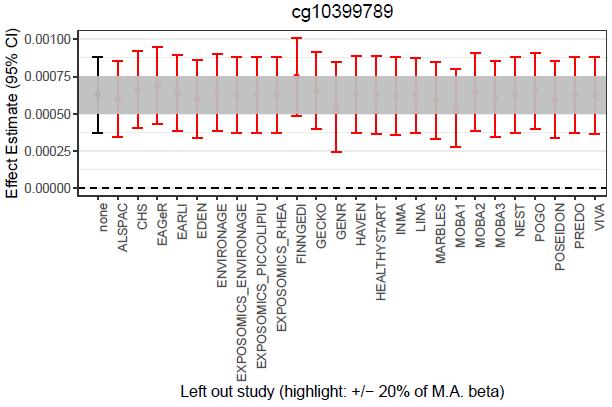


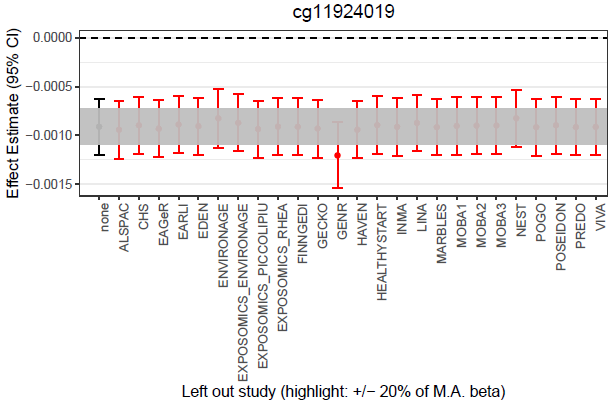

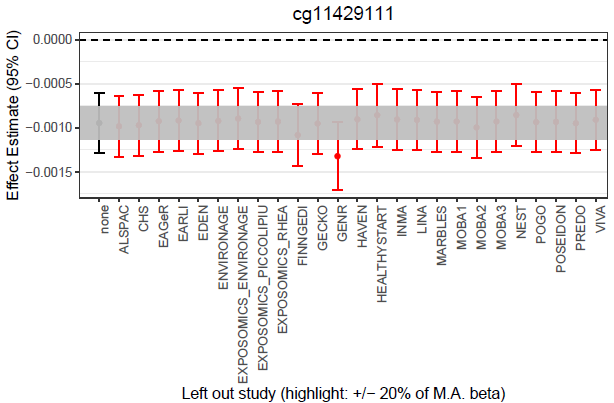


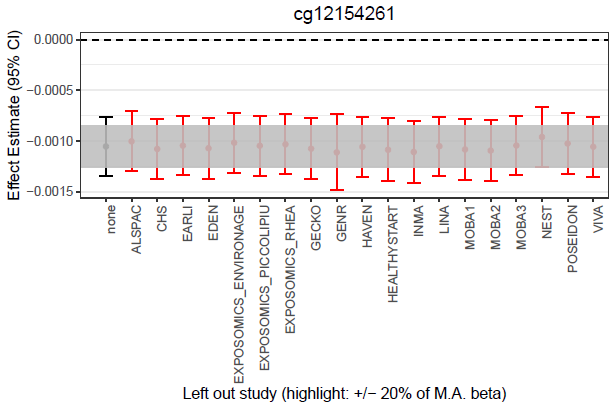

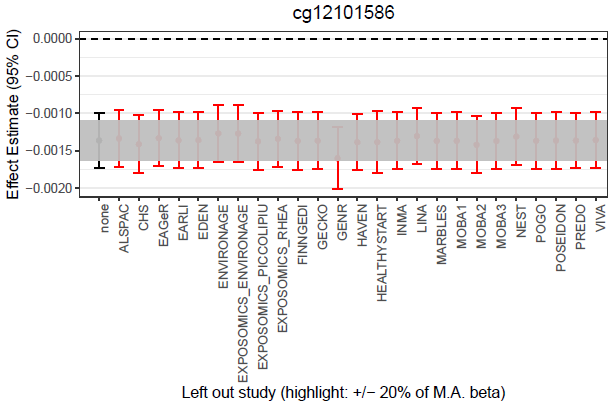


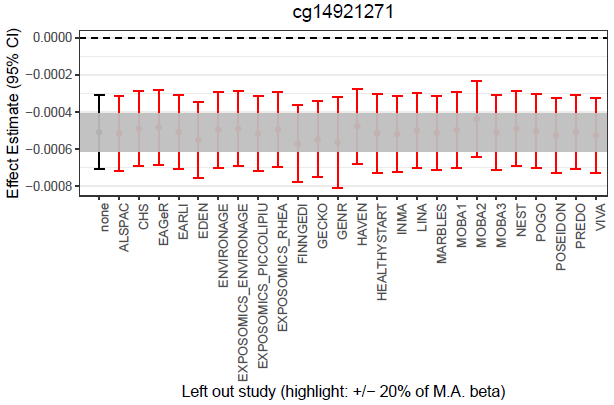

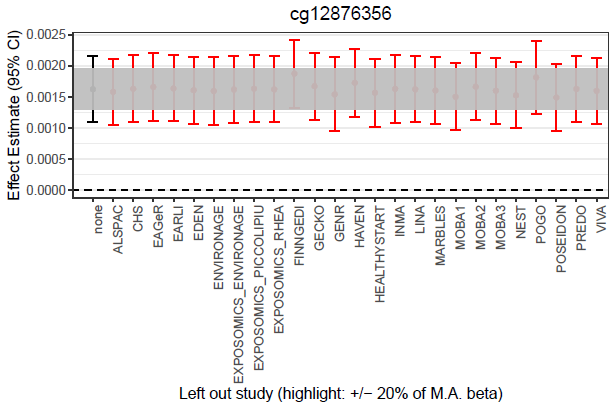


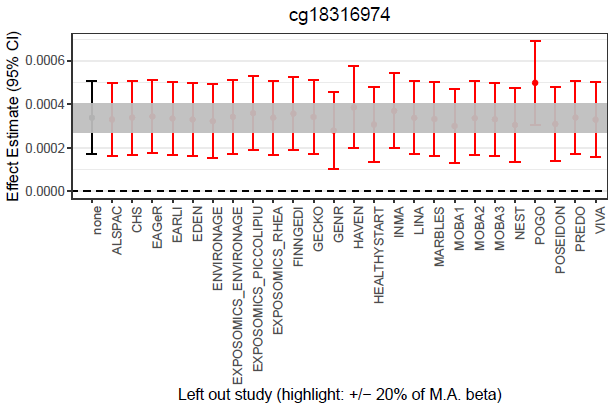


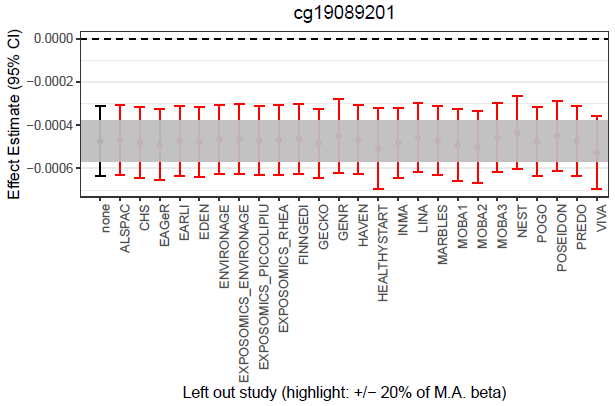


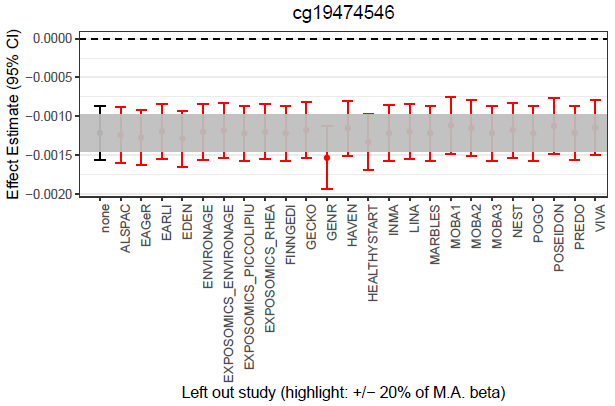

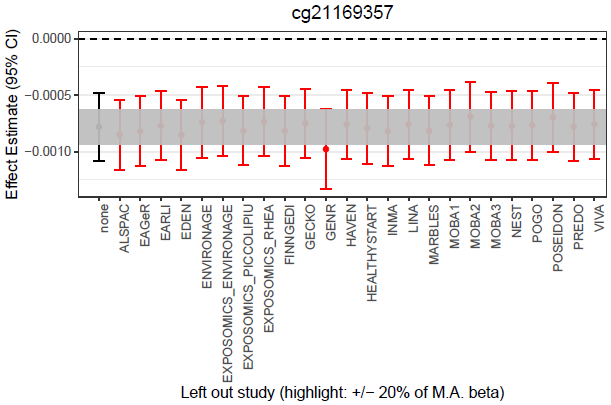


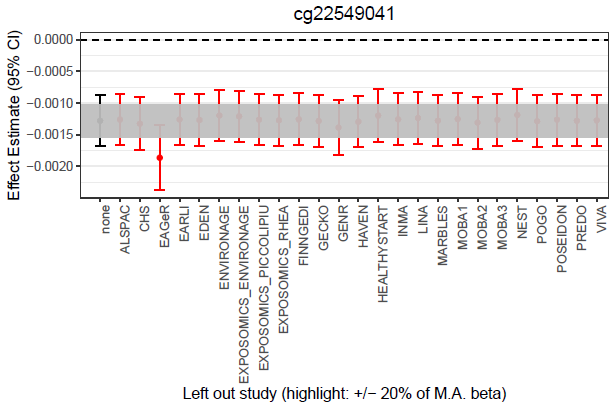

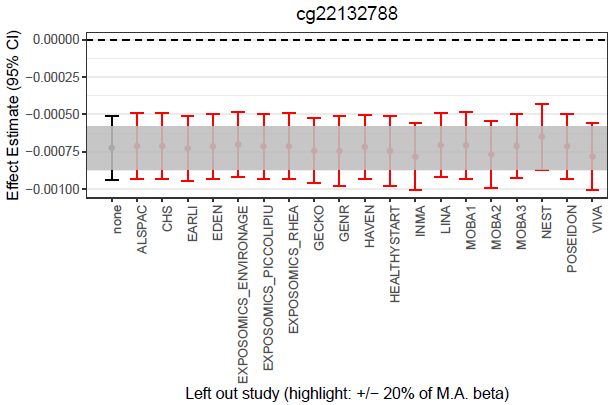


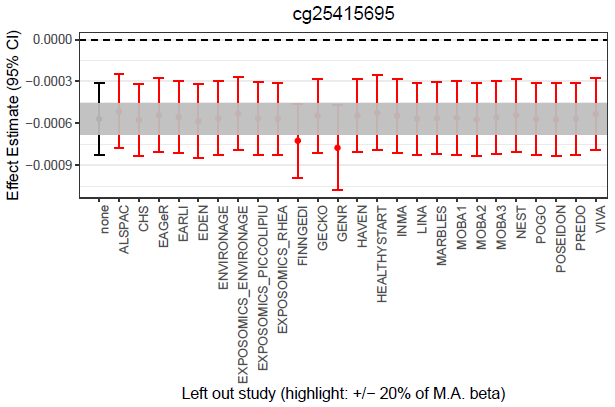

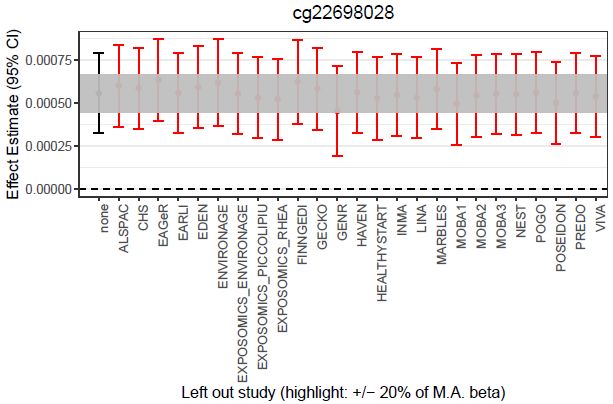


# **Figure 5.** Leave-one-out analysis (systematically leaving one CpG at a time out of the meta-analysis) for the main model, for 24 CpGs with I^2^>50%.


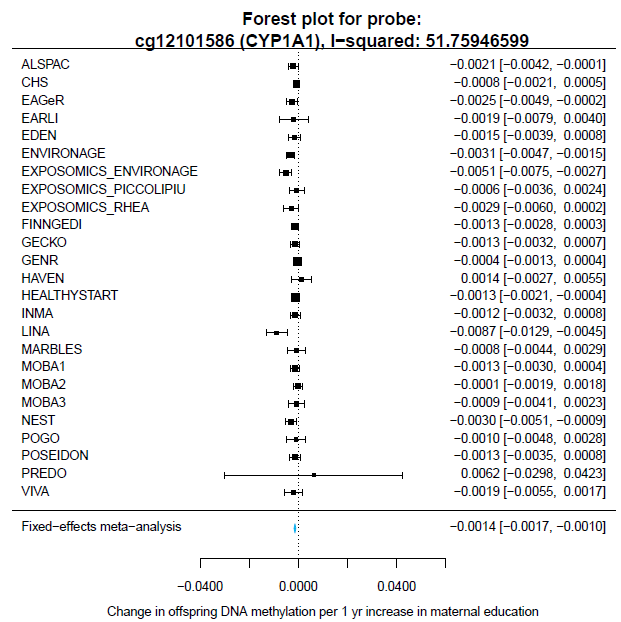


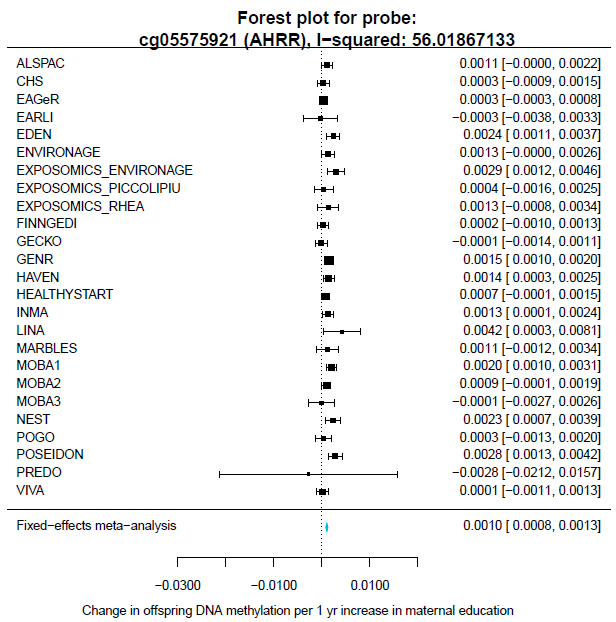


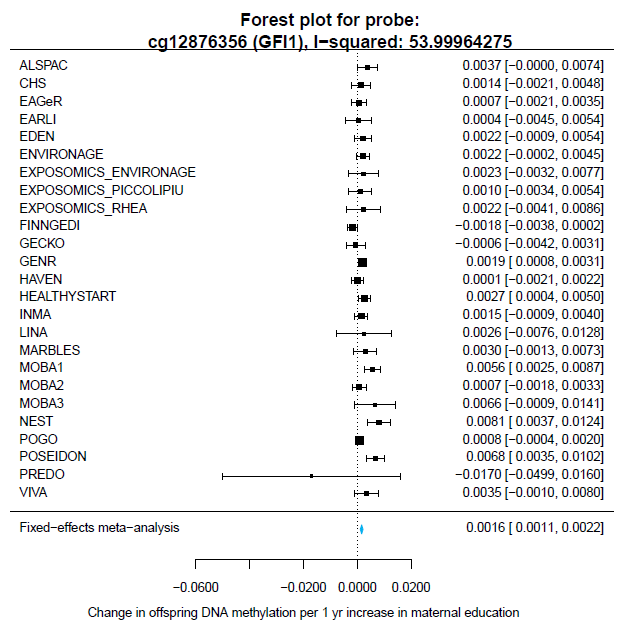

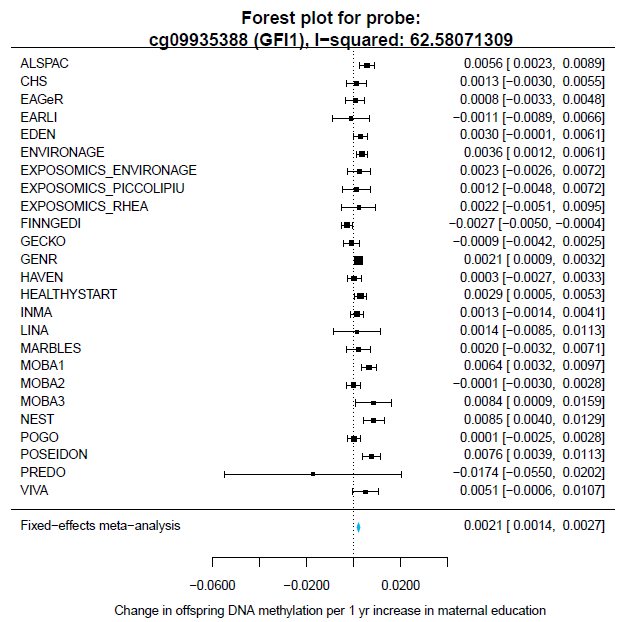


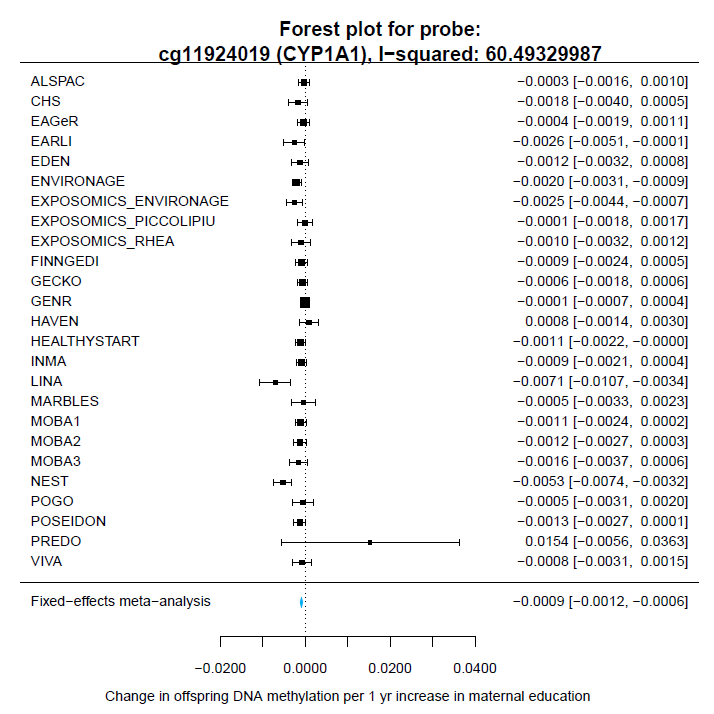

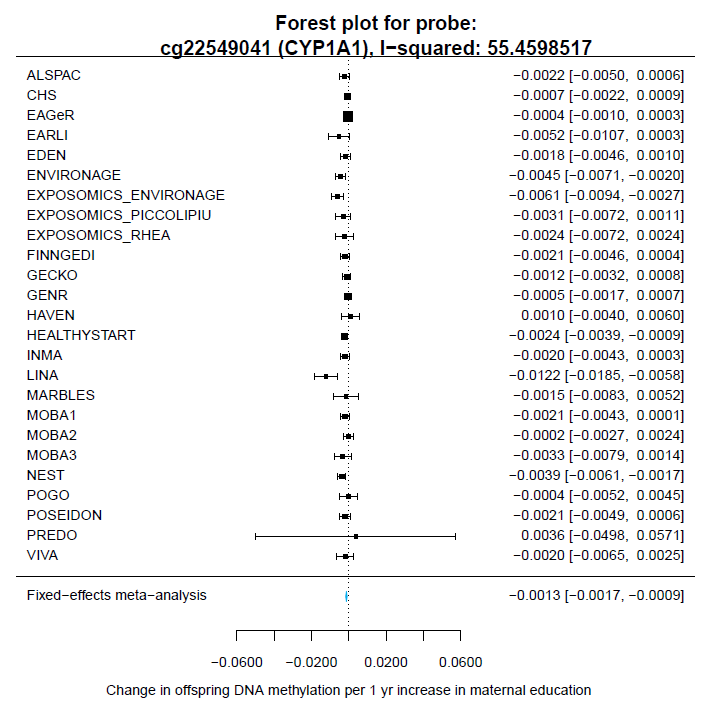


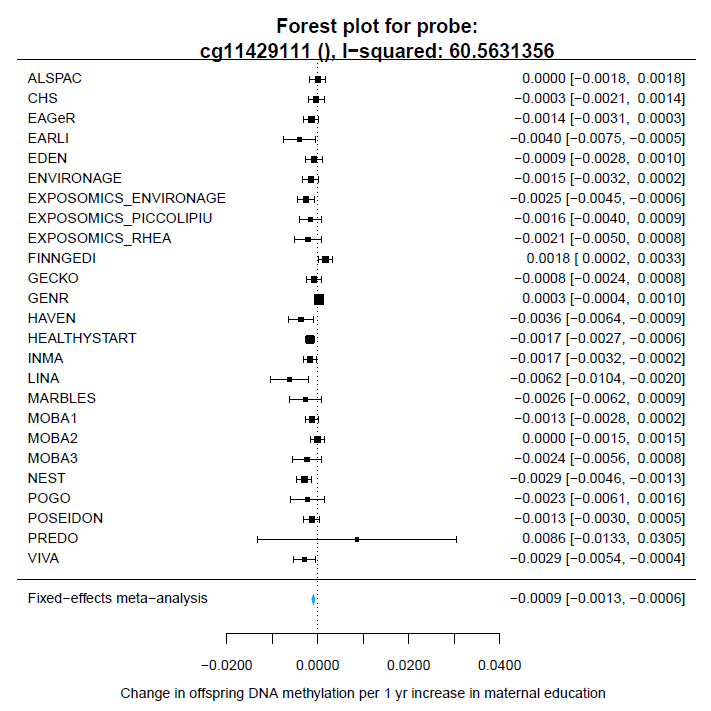


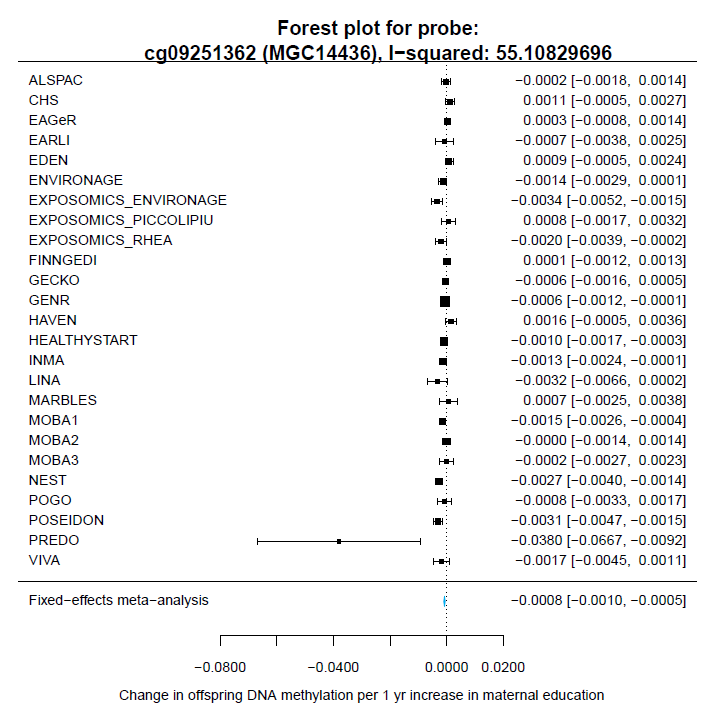


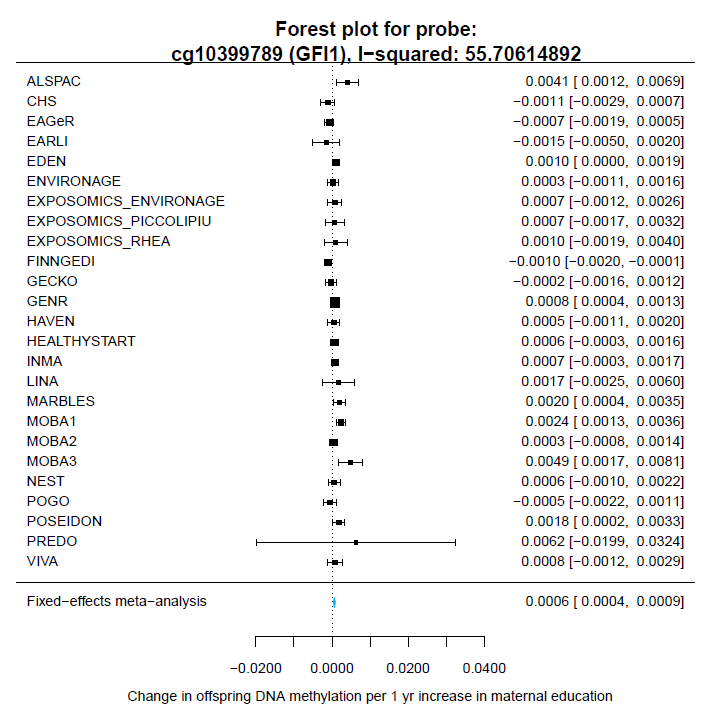

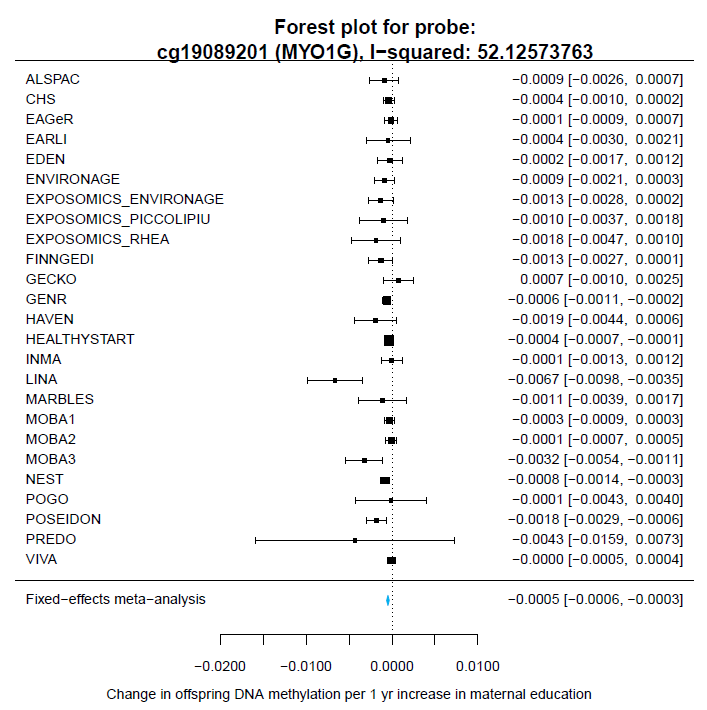


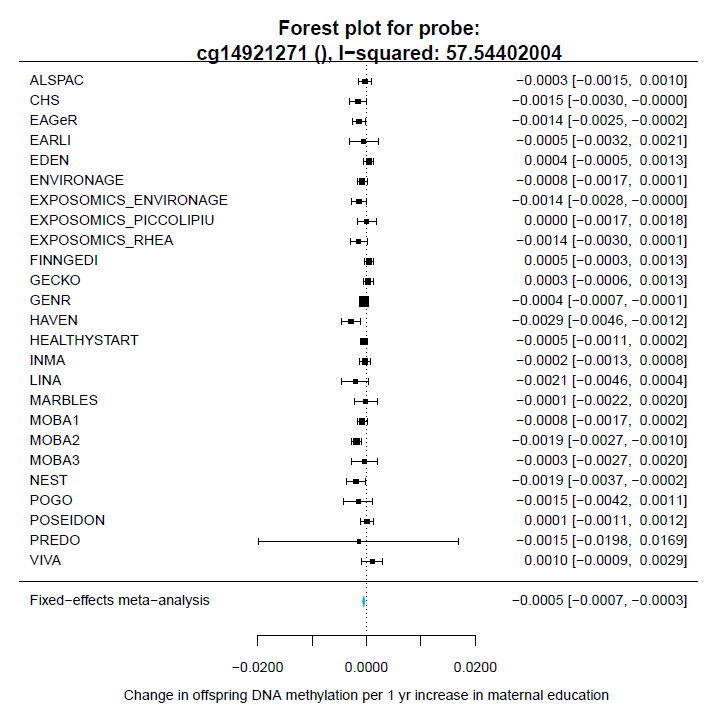

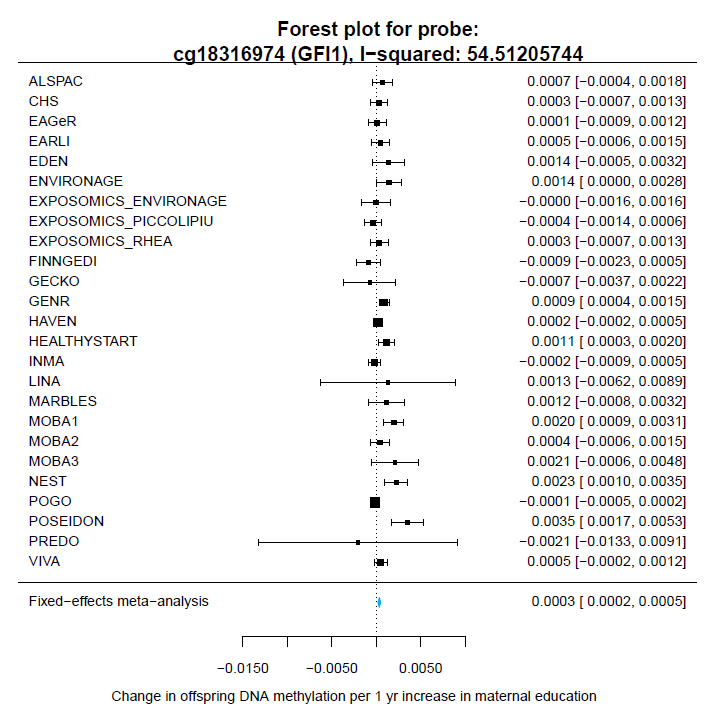


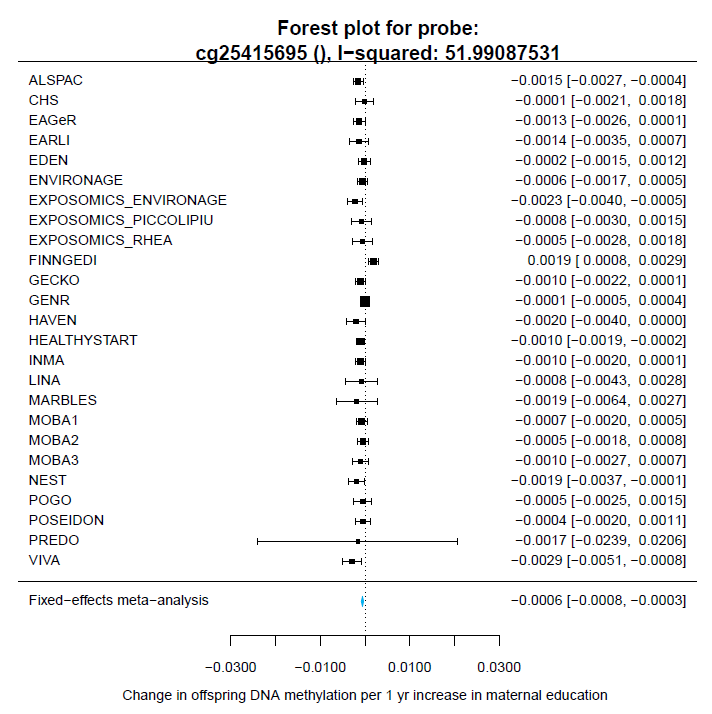

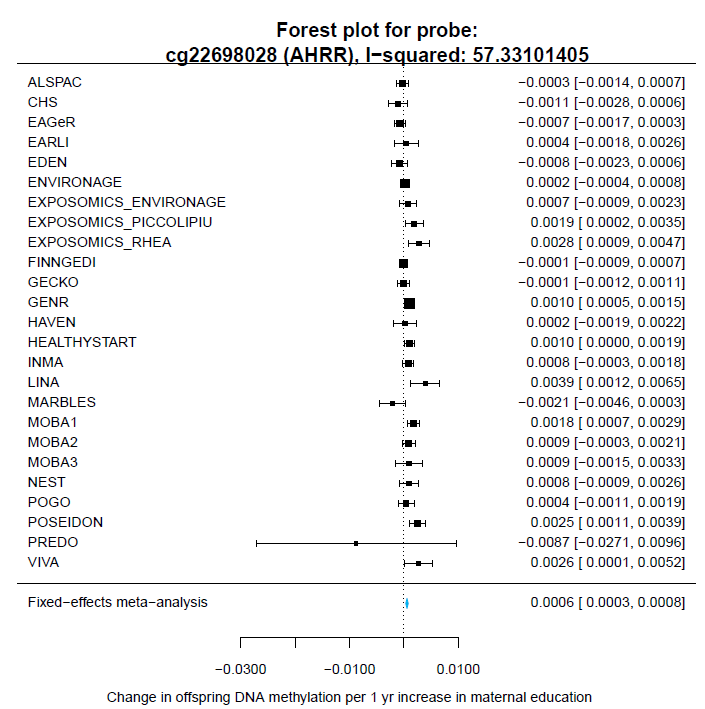


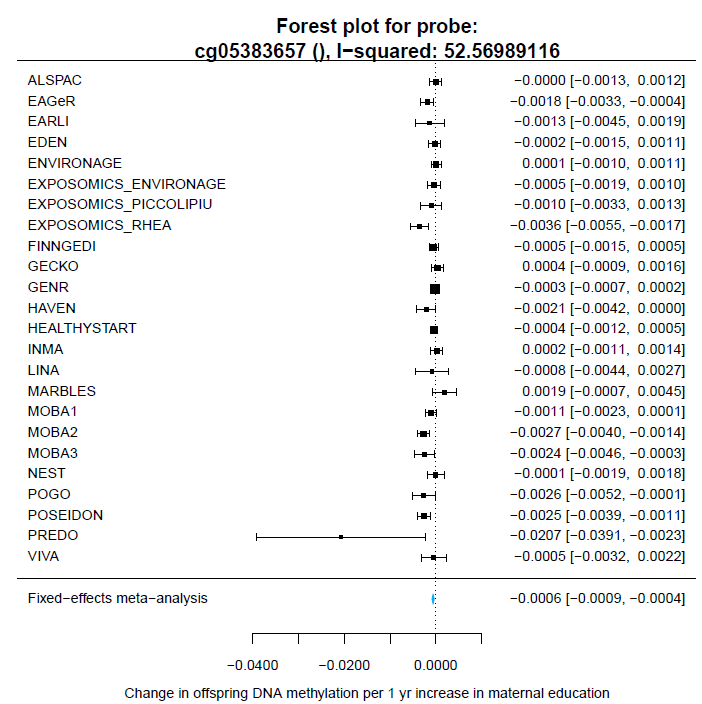

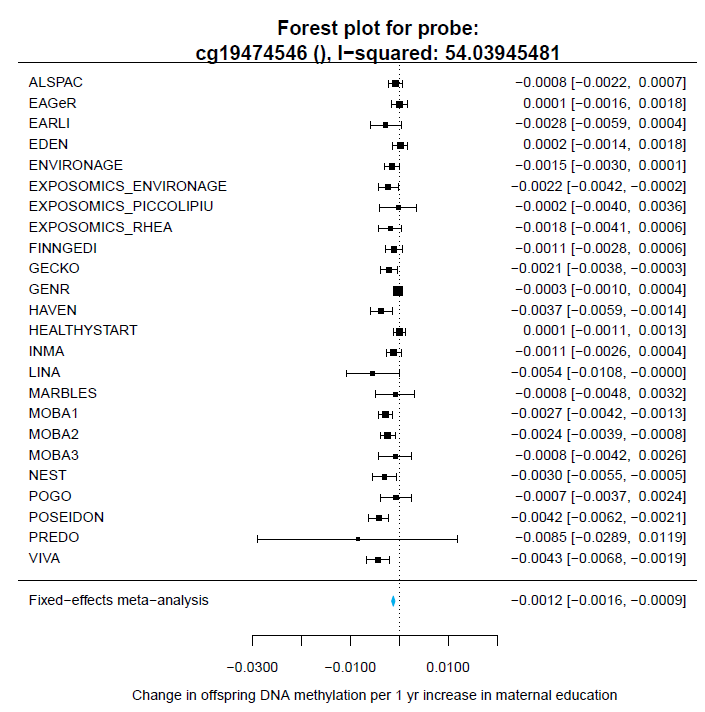


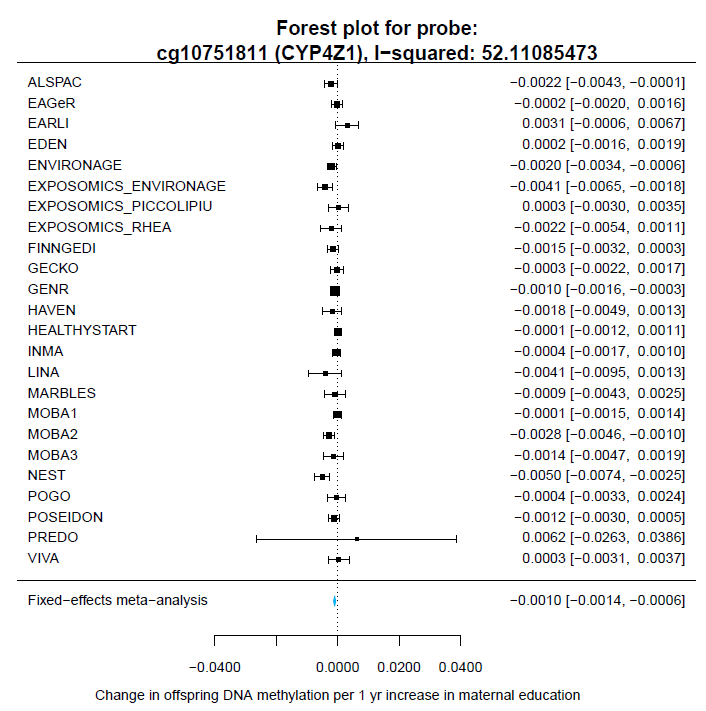

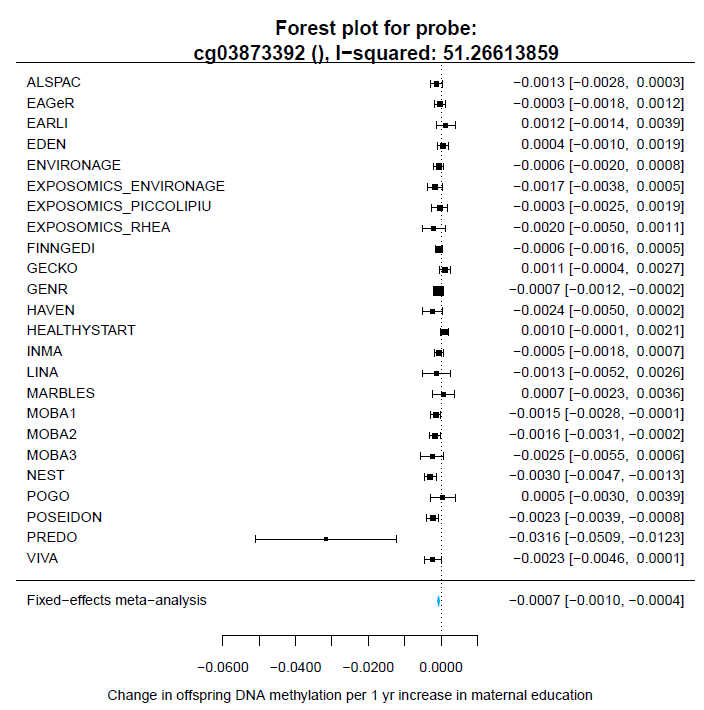


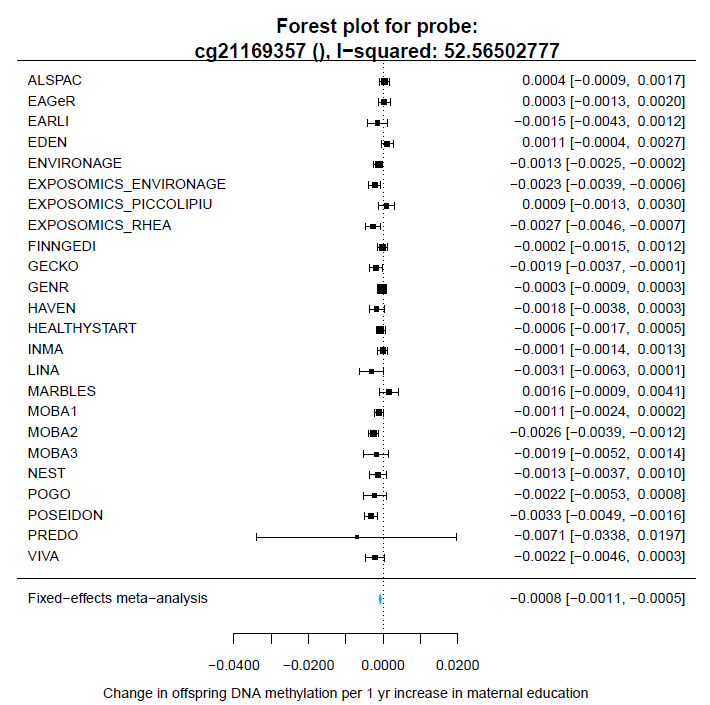

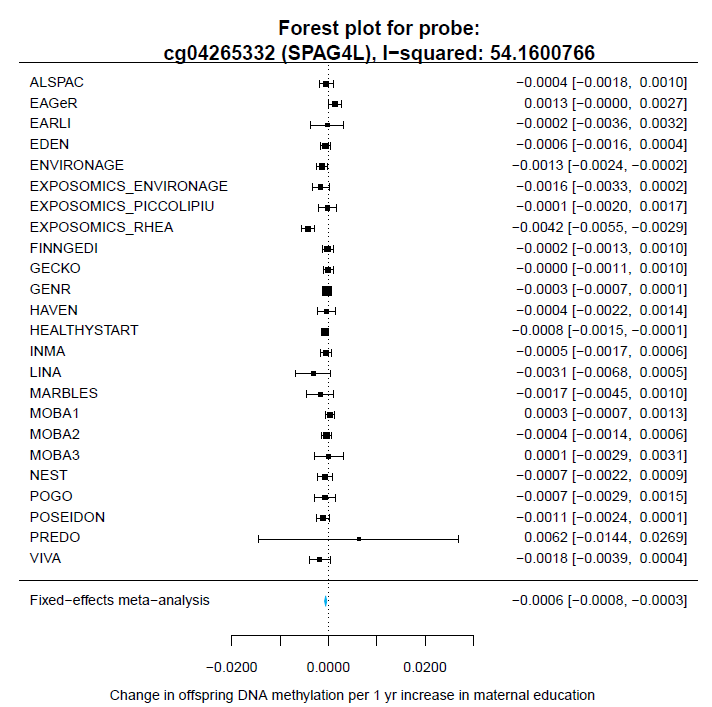


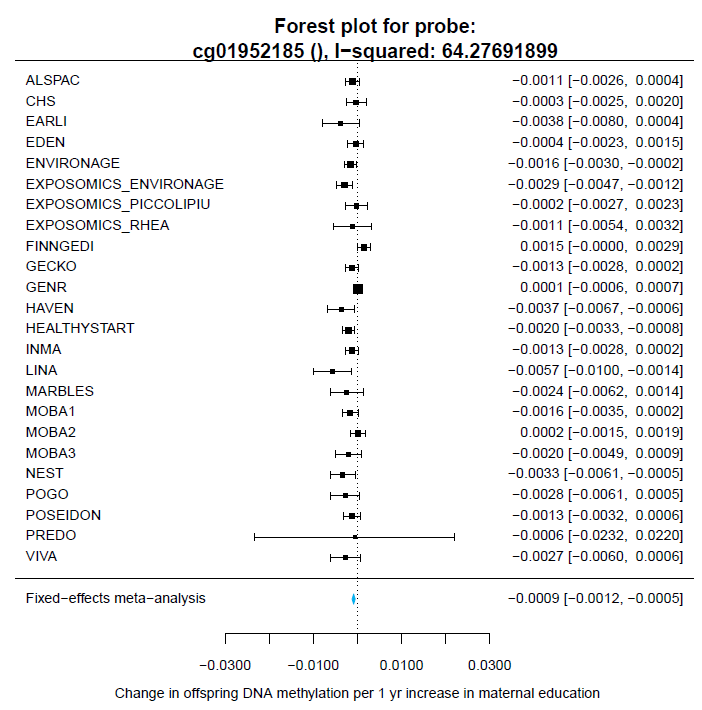

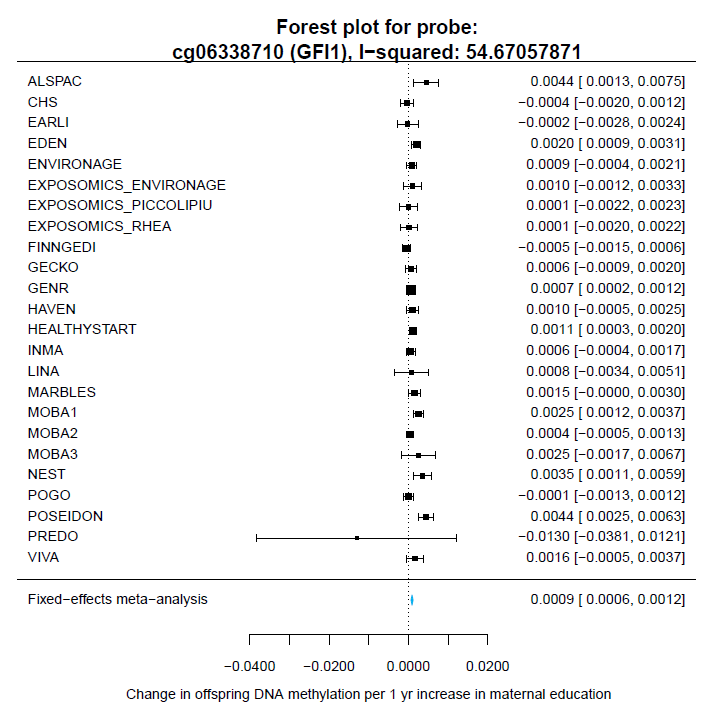


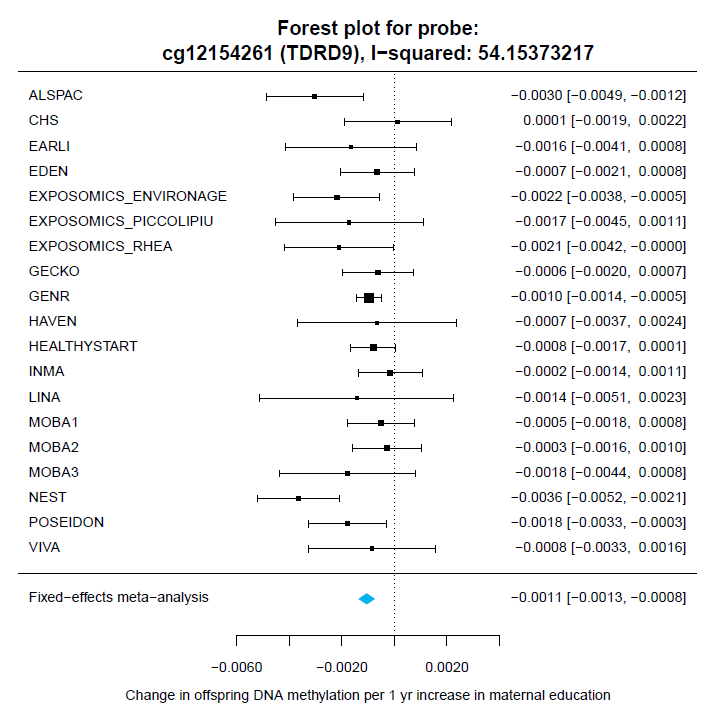

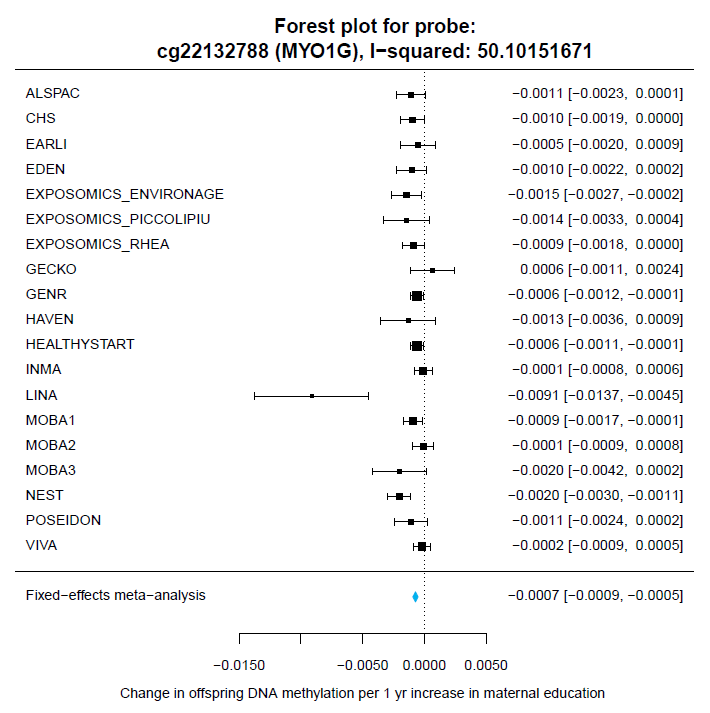


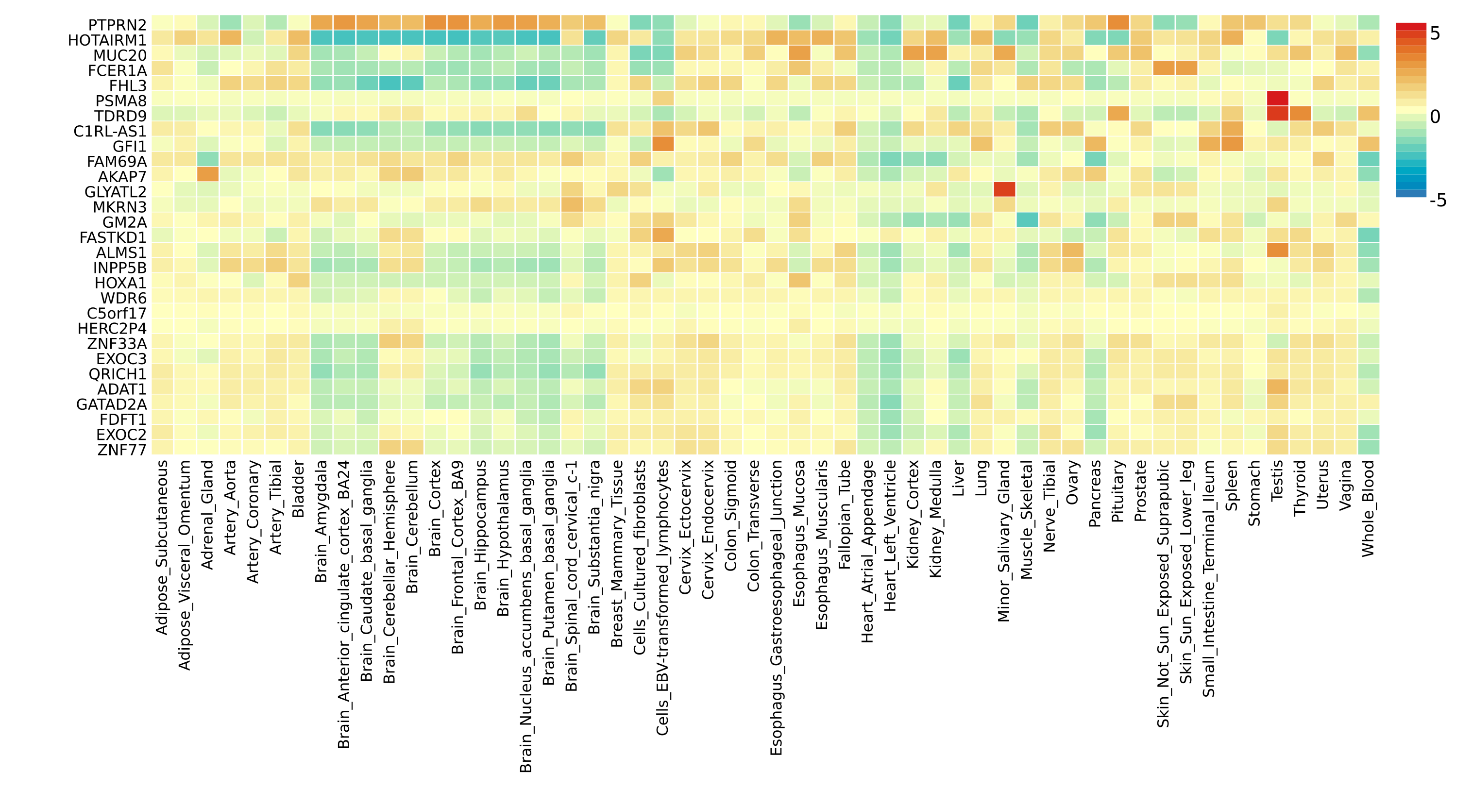


Tissue types

Genes

# **Figure 6**. Heatmap of the expression analysis using GTEx expression

Note: The heatmap created with the webtool FUMA, displays the normalized expression for the genes in proximity with the 29 unique transcript clusters identified in the *cis*-eQTM. The color spectrum represents the normalized relative expression across tissues per gene (Reads Per Kilo base per Million, RPKM). Multiple clusters were observed as more highly expressed in brain tissues compared to the other tissues. The clusters are located between “Brain_Amygdala” and “Brain_Substantia _nigra” tissues.


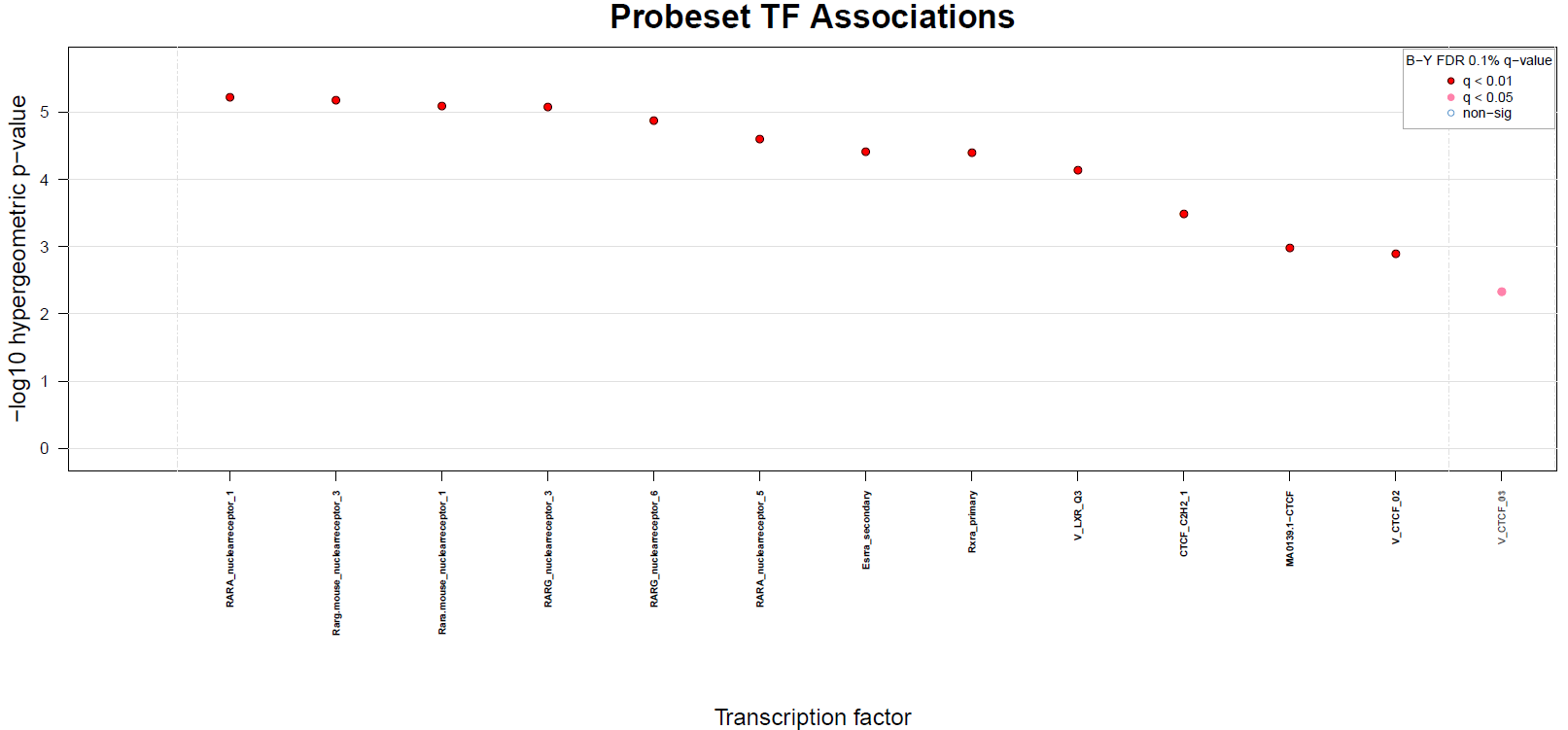


# **Figure 7**. Tissue specific enrichment from eForge analysis in the adolescent analysis

# **Cohort-specific methods**

## Avon Longitudinal Study of Parents and Children (ALSPAC)

ALSPAC is a prospective cohort study based in the Southwest of England, UK. Pregnant women resident in Avon, UK with expected dates of delivery 1st April 1991 to 31st December 1992 were invited to take part in the study. Pregnant women resident in Avon, UK with expected dates of delivery between 1st April 1991 and 31st December 1992 were invited to take part in the study. 20,248 pregnancies have been identified as being eligible and the initial number of pregnancies enrolled was 14,541. Of the initial pregnancies, there was a total of 14,676 foetuses, resulting in 14,062 live births and 13,988 children who were alive at 1 year of age. When the oldest children were approximately 7 years of age, an attempt was made to bolster the initial sample with eligible cases who had failed to join the study originally. As a result, when considering variables collected from the age of seven onwards (and potentially abstracted from obstetric notes) there are data available for more than the 14,541 pregnancies mentioned above: The number of new pregnancies not in the initial sample (known as Phase I enrolment) that are currently represented in the released data and reflecting enrolment status at the age of 24 is 906, resulting in an additional 913 children being enrolled (456, 262 and 195 recruited during Phases II, III and IV respectively). The phases of enrolment are described in more detail in the cohort profile paper and its update (see footnote 5 below). The total sample size for analyses using any data collected after the age of seven is therefore 15,447 pregnancies, resulting in 15,658 foetuses. Of these 14,901 children were alive at 1 year of age.

Of the original 14,541 initial pregnancies, 338 were from a woman who had already enrolled with a previous pregnancy, meaning 14,203 unique mothers were initially enrolled in the study. As a result of the additional phases of recruitment, a further 630 women who did not enrol originally have provided data since their child was 7 years of age. This provides a total of 14,833 unique women (G0 mothers) enrolled in ALSPAC as of September 2021. Detailed information has been collected on these women, their partners and their offspring at regular intervals to the present date. Please note that the study website contains details of all the data that is available through a fully searchable data dictionary and variable search tool: <http://www.bristol.ac.uk/alspac/researchers/our-data/>.^1,2^

Written informed consent has been obtained for all ALSPAC participants. Ethical approval for the study was obtained from the ALSPAC Ethics and Law Committee and the Local Research Ethics Committees. Consent for biological samples has been collected in accordance with the Human Tissue Act (2004). Informed consent for the use of data collected via questionnaires and clinics was obtained from the participants following the recommendations of the ALSPAC Ethics and Law Committee at the time.

As part of the Accessible Resources for Integrated Epigenomic Studies (ARIES, <http://www.ariesepigenomics.org.uk/>) project, DNA methylation was generated for 1,018 mother-offspring pairs from the ALSPAC cohort, using the Infinium HumanMethylation450 BeadChip array (Illumina Inc., San Diego, United States). ARIES participants were selected based on availability of DNA samples at two time points for the mother (antenatal and at follow-up when the offspring were adolescents) and at three time points for the offspring (newborn, childhood (age 7), and adolescence (age 15/17)). The current study used child cord blood at birth and whole blood at age 7 and age 15/17.

*Maternal Education*

Maternal age at delivery was derived from the mother’s date of birth. Maternal socio-economic status (SES) was defined based on the UK highest qualification achieved by the mothers, with the maximum level of education being “Degree”, and consists of 5 categories: CSE/none, Vocational, O-level, A-level and university degree. The variable was re-coded to US years-of-schooling equivalent in accordance with the ISCED 1997 classification (1997). In order to best reflect the length participants spent in education, participants with Vocation and O-level were combined and to the original ALSPAC categories were assigned the following total years of education: CSE/none= 10yrs; Vocational and O-level= 13 yrs; A-level: 15 yrs; Degree= 20 yrs.

*Methylation measurements*

Methods for methylation measurements in ALSPAC have been described previously ^3^. Briefly, cord and whole blood were collected according to standard procedures. DNA methylation assays and data pre-processing were performed at the University of Bristol as part of the ARIES project. DNA was extracted using standard protocol and was bisulfite-converted using the Zymo EZ DNA MethylationTM kit (Zymo, Irvine, CA). DNA methylation was then measured using the Infinium HM450 BeadChip assay (Illumina Inc, San Diego, CA), according to the standard protocol. Arrays were scanned using an Illumina iScan. An initial review of data quality was assessed using GenomeStudio (version 2011.1). A semi-random approach (sampling criteria were in place to ensure that all time points were represented on each array) was used to distribute ARIES samples across slides to minimize the possibility of potential confounding by batch. Data were normalised using the meffil R package (4) using the functional normalisation approach. In this study, methylation outliers were removed using the IQR*3 (Tukey) method. The final DNA methylation dataset contained information on 471,248 CpGs.

*Covariates*

Gestational age was calculated (in days) based on the date of the mother’s last menstrual period (LMP) when the mother was certain of this, but for uncertain LMPs and conflicts with clinical assessment the ultrasound assessment was used. Where maternal report and ultrasound assessment conflicted, an experienced obstetrician reviewed clinical records and made a best estimate. Gestational age in weeks was used in the analyses.

Child BMI for age 7 and age 15/17 were derived by the height (measured to the last complete mm using the Harpenden Stadiometer) and weight (measured using the Tanita Body Fat Analyser) measured during the study clinic.

Child smoking at age 15/17 was derived by three distinct smoking variables: ever smoked, smoked weekly, number of cigarettes smoked in lifetime. Adolescents were classified as smokers if they smoked weekly or didn't smoke weekly but have smoked 50+ cigarettes in entire life. They were classified as non-smoker if they never tried smoking or they didn't smoke weekly and smoked less than 50 cig/life.

Age of the child at attendance was calculated from the date of the visit and the child’s date of birth.

Maternal age at delivery was derived from the mother’s date of birth.

Maternal body mass index (BMI) was calculated from self-reported height and pre-pregnancy weight, which were collected by questionnaire during the first trimester of pregnancy.

Maternal smoking during pregnancy was determined by questionnaire at the time of recruitment and defined as smoking in every trimester of the pregnancy.

Newborn sex was obtained from obstetric records.

*Cell type correction*

Estimation of blood cell type proportions was obtained from methylation data applying the Houseman method, and the Bakulski and Reinius’ reference panel, respectively, for cord and child blood.

*Batch correction*

We attempted to control for technical batch effects by generating surrogate variables using the SVA R package^4^ and including these directly in our models.

*Exclusion criteria*

Participants with non-white European ancestry were excluded from all analyses.

## Children’s Health Study (CHS)

Design and study population

The Children’s Health Study (CHS) is a population-based prospective cohort study from age 5 onwards in Southern California, which has been described in detail elsewhere. A total of 5341 children were recruited, all of whom were born between 1995 and 1997 and were followed until age 18. Based on the availability of newborn bloodspots archived by the state of California, 273 children were selected for a sub-study in which epigenome-wide DNA methylation was assessed in newborn bloodspots using the Infinium HumanMethylation450 BeadChip (HM450).

Ethics statement

The study protocol was approved by the University of Southern California Institutional Review Board and informed, written consent and assent were provided by the parents and children respectively.^5^

Maternal education

Maternal education at the time of the pregnancy was obtained from the participants’ birth records. The CHS maternal education variable was originally coded as the highest grade completed by the mother, including pre-elementary, 1st grade to 12th grade, 1 year to 4 year college, and 5 or more years college. These categories were recoded in accordance with the ISCED 1997 classification equivalent to US years of schooling in the study.

Methylation measurements

Laboratory personnel performing DNA methylation analysis were blinded to study subject information. DNA was extracted from whole blood cells using the QiaAmp DNA blood kit (Qiagen Inc, Valencia, CA) and stored at -80 degrees Celcius. 700-1000ng of genomic DNA from each sample was treated with bisulfite using the EZ-96 DNA Methylation Kit™ (Zymo Research, Irvine, CA, USA), according to the manufacturer’s recommended protocol and eluted in 18 ul. The results were compiled for each locus and were reported as beta (β) values.^6^ Quality control of analyzed samples was performed using standardized criteria. Illumina Infinium 450K data were processed in the minfi package (version 1.16.0) in R,^7^ after which a normal-exponential background correction with dye bias correction was applied to the raw intensities at the array level to reduce background noise.^8^ We then normalized each sample’s methylation values to have the same quantiles to address sample to sample variability.^9^ CpG loci on the HM450 array were removed from analyses if they contained SNPs, deletions, repeats, or if they have more than 10% missing values, leaving a total of 394, 415 probes for the current analysis. Beta values were considered outliers and were removed if they fell below Quartile 1-(3×IQR) or above Quartile 3+(3×IQR).

Covariates

Regression models were adjusted for newborn sex, plate number (batch), and cord blood cell subpopulations among the overall population and ethnicity-specific stratum. In addition, for the overall population analysis only, genetic ancestry (European, African, and Asian) determined by ancestry informative SNPs was further adjusted for. Information on infant sex was obtained from the participants’ birth records.

Cell type correction

Seven cord blood cell sub-populations were estimated using the combined cord blood cell reference with the EstimateCellCounts2 function, as described by Salas et al.sa Estimated cord blood cell subpopulations (CD8+ T-lymphocytes, CD4+ T-lymphocytes, natural killer cells, B-lymphocytes, monocytes, granulocytes and nucleated red blood cells) were subsequently included as linear predictors in regression models.

Batch correction

Plate number (batch) was adjusted in the regression models.

Exclusion criteria

We excluded 26 participants who had missing values for maternal education or covariates, including infant sex, plate, cord blood cell types, and genetic ancestry. In Hispanic ethnicity-specific analyses, 27 participants of Asian and other racial/ethnic groups were excluded, leaving 142 Hispanic white and 78 non-Hispanic white in each stratum.

## Effect of Aspirin in Gestation and Reproduction (EAGeR)

*Design and study population*

The Effects of Aspirin in Gestation and Reproduction (EAGeR) trial (2007-2011; NCT00467363) randomized women prior to conception who previously experienced 1-2 prior pregnancy losses to 81 mg low dose aspirin and 400 μg folic acid, or placebo plus 400 μg folic acid.^10^ Cord blood was collected from 428 deliveries at the Utah site.^11^ We previously found that randomization to low dose aspirin did not significantly alter DNA methylation levels at birth.

*Ethics statement*

The University of Utah IRB approved the study (Salt Lake City, Utah IRB #1002521) and all participants provided written informed consent prior to enrolment.

*Maternal education*

Women were asked at baseline, “What is the highest grade or level of school you have completed or the highest degree you have received?” Responses include: 1) Never attended/kindergarten only, 2) Not High school graduate, specify grade, 3) high school, 4) GED or equivalent, 5) Some college, no degree, 6) Associates degree (occupational, technical, vocational program), 7) Associates degree (academic program), 8) Bachelor’s Degree, 9) Master’s Degree, 10) Professional School Degree, and 11) Doctoral Degree. Categories were coded as specified in the ISCED 1997 equivalent of US years of schooling.

*Methylation measurements*

Cord blood DNA underwent bisulphite conversion with standardized kits (e.g. Zymo EZ DNA MethylationTM kit, Zymo, Irvine, CA), followed by measurements of DNA methylation using the Infinium MethylationEPIC BeadChip microarray. Methylation data were processed using the minfi package in R. Quantile normalization was used to normalize beta values between two types of probes. Sex mismatch was examined using principal components analysis. A detection P-value > 0.01 was used to identify methylation measures which failed detection. β values were replaced as missing if they failed detection or had bead counts < 3. Betas were trimmed using the 3IQR trimming method. We removed samples and CpG sites with low passing rate (<97%) based on detection p-value and bead counts. After probe removal, 801,761 CpG probes remained. Additional removal of probes with >10% missing resulted in 793,678 probes.

*Covariates*

Women reported information on smoking, which was dichotomized as any versus never due to few numbers. Race/ethnicity was also self-reported. Maternal age and gestational age were abstracted from medical records. Maternal BMI (kg/m^2^) was calculated based on clinically measured weight and height at baseline prior to pregnancy.

*Cell type correction*

“FlowSorted.CordBlood.Combined.EPIC’’ Bioconductor package was implemented for cell type correction, including the following cell types: CD8T, CD4T, NK, Bcell, Mono, Gran, nRBC.^12^

*Batch correction*

Batch was corrected inclusion of sample plate indicators into the models.

*Exclusion criteria*

The current analysis included 379 newborns with DNA methylation data available, after excluding 30 with insufficient DNA, 5 sex mismatch, 1 twin, 1 failing detection cutoff, and 12 non-White participants.

## Early Autism Risk Longitudinal Investigation cohort (EARLI)

*Study design and cord blood sample*

The Early Autism Risk Longitudinal Investigation (EARLI) is an enriched risk prospective pregnancy cohort to study autism etiology. The EARLI study was reviewed and approved by Human Subjects Institutional Review Boards (IRBs) from each of the four study sites (Johns Hopkins University, Drexel University, University of California Davis, and Kaiser Permanente Northern California). Participants in the study provided written informed consent. This longitudinal study recruited mothers of confirmed ASD children who were early in a subsequent pregnancy or were trying to become pregnant. There were 232 mothers with a subsequent sibling born through this study. All children were born between November 2009 and March 2012. Demographics, maternal behaviors, food frequency, medical history were all collected via questionnaire. Biosamples and were collected during pregnancy, at birth, and during development. At birth, samples including cord blood and placenta were collected and archived at 213 births.^13^

*Maternal education measures*

Mothers were asked early in pregnancy to report their highest level of attained education. Education level was standardized by meta-analysis procedures. In this sample, 3 (1.9%) mothers had primary education, 2 (1.3%) had lower secondary, 12 (7.7%) had secondary education, 51 (33%) had post-secondary non-tertiary education, 47 (30.3%) had first stage of tertiary education, and 40 (25.8%) had second stage of tertiary education (masters or PhD graduate degree). In education years using ISCED 1997 classification, this distribution had a mean of 17.6 (sd 3.5, IQR 7).

*DNA methylation measures*

Cord blood DNA was extracted using the DNA Midi kit (Qiagen, Valencia, CA) and samples were bisulfite treated and cleaned using the EZ DNA methylation gold kit (Zymo Research, Irvine, CA). DNA was plated randomly and assayed on the Infinium HumanMethylation450 BeadChip (Illumina, San Diego, CA) at the Johns Hopkins SNP Center, a shared lab and informatics operation with the Center for Inherited Disease Research (Johns Hopkins University). Methylation control gradients and between-plate repeated tissue controls were used.

We used the minfi library (version 1.18.2) in R (version 3.3) to process raw Illumina image files into noob background corrected methylation values.^7,8^ Probes with failed detection P-value (>0.01) in >10% of samples were removed (n=661). Samples with discordant methylation predicted sex and observed sex were removed (n=3) as were samples that appeared as outliers on the first principal component of methylation data across the genome prior to normalization (n=2). 3IQR trimming of outlier beta values was done according to meta-analysis specifications.

There were 170 cord blood measures that passed DNA methylation quality control with 455,698 probes. A total of 155 participants had complete data for covariates.

*Covariates*

Self-reported smoking was low in this cohort. We excluded 6 mothers who self-reported any smoking during pregnancy. In the cross-ancestry analysis, ancestry was adjusted for using the first two principal components of the genetic data. In the European genetic ancestry only analysis, the sample was subset to 97 mothers of European genetic ancestry. Gestational age, maternal age at delivery and maternal pre-pregnancy BMI were collected from medical records. There were 79 (50.3%) male children. Mothers had an average age of 33.4 years (sd 4.6, IQR 5.0). Gestational age had an average of 39.4 weeks (sd 1.3, IQR 1.1). Maternal pre-pregnancy BMI had an average of 28.0 (sd 7.3, IQR 7.9). There were two laboratory batches, represented by two hybridization dates, with 104 samples (67.1%) in the first batch. Blood cell types were estimated using the Houseman method with a combined cord blood reference set.^12^

## Markers of Autism Risk Learning Early Signs (MARBLES)

*Study design and placental sample*

Markers of Autism Risk Learning Early Signs (MARBLES) is an enriched risk prospective pregnancy cohort to study autism etiology. The MARBLES protocol was reviewed and approved by the Human Subjects Institutional Review Board (IRB) from University of California Davis. This ongoing longitudinal study recruited mothers of children with confirmed autism spectrum disorder who were in a subsequent pregnancy or were trying to become pregnant.^14^ Participants in the study provided written informed consent. At the time of this analyses, there were 389 enrolled mothers that gave birth to 425 subsequent siblings between December 1, 2006 and July 1, 2016. Demographics, maternal behaviors, food frequency, medical history were all collected via questionnaire. Biospecimens were collected during pregnancy, at birth, and during development. At the delivery hospital, cord blood samples were collected and immediately processed and frozen. Cord blood samples were stored at -80 degrees Celsius in the UC Davis biorepository.

*Maternal education measures*

Mothers were asked early in pregnancy to report their highest level of attained education. Education level was standardized by meta-analysis procedures. In this sample, 10 (2.8%) had lower secondary, 15 (6.1%) had secondary education (GED), 102 (41.5%) had post-secondary non-tertiary education (some college, no degree), 74 (30.1%) had first stage of tertiary education (Bachelor’s degree), and 48 (19.5%) had second stage of tertiary education (masters, professional, or PhD graduate degree) In education years using ISCED 1997 classification, this distribution had a mean of 17.3 (sd 3.2, IQR 4).

*DNA methylation measures*

Cord whole blood samples were transferred to the Johns Hopkins Biological Repository (JHBR) laboratory where cord blood DNA was extracted from a subsample of 279 participants with 36-month diagnostic assessment data available using the DNA Midi kit (Qiagen, Valencia, CA). Samples were bisulfite treated and cleaned using the EZ DNA methylation gold kit (Zymo Research, Irvine, CA). DNA was plated randomly and assayed on the Infinium HumanMethylationEPIC BeadChip (Illumina, San Diego, CA) at the Johns Hopkins SNP Center, a shared lab and informatics operation with the Center for Inherited Disease Research (Johns Hopkins University). DNA methylation control gradients and between-plate repeated tissue controls were used.

Samples that has mismatched predicted sex were dropped (n=3). There were 276 cord blood samples that passed DNA methylation quality control. We further excluded samples from non-singleton births (n=11). Probes were dropped (n=4,633) if they had detection-p (p>0.01) failure in greater than 5% of samples. Cross reactive probes (n=42,967) were also dropped.^15^ We used the minfi library (version 1.30.0) in R (version 3.6) to process raw Illumina image files into noob background corrected methylation values. 3IQR trimming of outlier beta values was done according to meta-analysis specifications.^7,8^ A total of 246 cord blood samples had complete data for covariates, with 818,259 probes.

*Covariates*

Self-reported smoking was low in this cohort. We excluded 10 mothers who smoked during pregnancy. Race/ethnicity was categorized using self-report and 102 (41.5%) mothers were Non-Hispanic White and 79 (32.1%) mothers were Hispanic. There were 145 (58.9%) male children. Gestational age, maternal age at delivery and maternal pre-pregnancy BMI were collected from medical records. Mean maternal pre-pregnancy BMI was 27.9 (sd 7.3, IQR 8.4); mean maternal age was 34.3 years (sd 4.7, IQR 7.2); and mean gestational age was 39.1 weeks (sd 1.2, IQR 1.1). We included plate as a batch variable, with samples spread across three plates with sample counts: 82 (33.3%), 83 (33.7%), and 81 (32.9%). Blood cell types were estimated using the Houseman method with a combined reference set.^12^

## EDEN

*Design and study population*

The EDEN (Etude des Déterminants pré et post natals du développement et de la santé de l′Enfant) study is a prospective Birth Cohort Study (https://eden.vjf.inserm.fr/), which has been described in detail elsewhere.^16^ Pregnant women seen for a prenatal visit at the departments of Obstetrics and Gynecology of the University Hospital of Nancy and Poitiers before their twenty-fourth week of gestation were invited to participate. Enrolment started in February 2003 in Poitiers and September 2003 in Nancy; it lasted 27 months in each centre. Among eligible women, 53% (n=2002) accepted to participate.

*Ethic statement*

All the participants included in this study provided written informed consent. The study has been approved by the ethical committees Comité Consultatif pour la Protection des Personnes dans la Recherche Biomédicale, Le Kremlin-Bicêtre University hospital, and Commission Nationale de l’Informatique et des Libertés.

*Collection of samples*

Immediately after delivery, cord blood samples were collected by research midwives from 1367 consenting cohort participants. To prevent any contamination with maternal blood, the cord was doubly clamped immediately after birth (vaginal delivery) or after extraction of the fetus through the uterine incision (elective cesarean section); repeatedly rinsed and venous cord blood serum was sampled between the 2 clamps. Whole blood samples were collected at the age of 5 years in 836 participants whose parents gave informed consent. Cord and whole blood samples were centrifuged within 24 hours of collection. The serum was separated and samples were stored at −80°C.

*Normalization and QC of methylation data*

DNA was extracted using the QIAamp blood kit (Qiagen or equivalent protocols), followed by precipitation-based concentration using GlycoBlue (Ambion). DNA concentration was determined by Nanodrop measurement and Picogreen quantification. 500 ng of DNA was bisulphite-converted using the EZ 96-DNA methylation kit (Zymo Research), following the manufacturer’s standard protocol. After verification of the bisulphite conversion step using Sanger Sequencing, genome-wide DNA methylation was measured using the Illumina Infinium HumanMethylation450 BeadChip. After normalization of the concentration, the samples were randomized to avoid batch effects, and all paired samples were hybridized on the same chip. Standard male and female DNA samples were included in this step as control samples.

DNA methylation data were pre-processed in R with the Bioconductor package Minfi,^7^ using the original IDAT files extracted from the HiScanSQ scanner. Samples that did not provide significant methylation signals in more than 10% of probes (detection P=0.01) were excluded from further analysis. Samples were also excluded in cases of low staining efficiency, low single base extension efficiency, low stripping efficiency of DNA from probes after single base extension, poor hybridization performance, poor bisulphite conversion and high negative control probe staining. Further, we used the 65 SNP probes to check for concordances between paired DNA samples from the sample individual and assessed the methylation distribution of the X-chromosome to verify gender. Paired samples with Pearson correlation coefficients <0.9 were regarded as sample mix-ups and were excluded from the study. Probes on sex chromosomes, probes that mapped on multi-loci, the 65 random SNPs assay and probes that contained SNPs at the target CpG sites with a minor allele frequency >10% were excluded during probe filtering.^17^ The allele frequencies of a list of SNPs were obtained from 1000 Genomes, release 20110521 for the CEU population. Finally, to correct Type I and Type II bias, the “DASEN” method was implemented to perform signal correction and normalization.^18^ After quality control, 439,306 autosomal probes remained in EDEN.

*Education years*

The highest school qualification obtained by the mother at the time of pregnancy was obtained through a standardized questionnaire. The conversion from highest educational qualification reported to the corresponding ISCED 1997 level and to the theoretical education years at the end of the studies has been done as it is shown in the supplementary file 4.

*Covariates*

Maternal age: measured at delivery; Maternal smoking during pregnancy: Maternal smoking during pregnancy was assessed by questionnaires in mid-pregnancy (20-24 weeks gestational age) and again immediately after delivery. Smoking during pregnancy was classified as “1” if they smoked both at 1st and 3rd trimester, or “0” otherwise. Gestational age: Gestational age was obtained from both the Medical Birth Registry and a questionnaire administered at enrolment based on ultrasound estimations and was used as a continuous variable in the analyses. Pre-pregnancy BMI: height was measured in clinic during mid-pregnancy, while pre-pregnancy weight was self-reported. Child sex: as reported in the obstetrical examination at birth. Child age: Age at the time of the blood sampling (for whole blood analyses). Child BMI: children’s weight and height were parental-reported measurements done by their paediatrician. Ancestry: EDEN methylation data were collected only on Caucasian children born to French-speaking mothers in the cities of Poitier and Nancy (France), and because of very homogenous ethnic background, no correction for ancestry was conducted. Cell type correction: Cell proportions were estimated using estimateCellCounts() in the minfi R package.^19^ Seven cell types (nRBC, CD8T, CD4T, NK, Bcell, Mono, Gran) were imputed for newborn cord blood methylation using Salas reference set (Gervin et al. 2019). For the whole blood samples collected at the age of 5 years, 6 cell types were imputed: CD8T, CD4T, NK, Bcell, Mono, Gran. Batch correction: Adjustment for batch effects was done by including sample plate as a covariate.

*Exclusion criteria*

n=5 participants without whole blood methylation information at the age of 5 were excluded from the whole blood methylation analyses. All EDEN participants were uniparous.

## ENVIRonmental influence ON early AGEing (ENVIRONAGE)

*Design and study population*

ENVIRONAGE (ENVIRonmental influence ON early AGEing) is an ongoing population-based prospective birth cohort study that aims at exploring the human ageing and its interaction with the environment.^20^ The cohort includes more than 2000 mother-infant pairs that have been recruited at the delivery at the East-Limburg Hospital in Genk (Belgium) from February 2010 onward. Inclusion criteria were: delivery without planned caesarean section and ability to fill out a Dutch language questionnaire.

*Ethics statement*

Ethical approval was obtained by the Ethical Committee of Hasselt University and the East-Limburg Hospital, and written informed consent was given by the parents.

*Maternal education*

Maternal education achievement was self-reported by the mothers at birth and was collapsed into the following categories: (i) no diploma (corresponding to 0 ISCED1997 level and 1 US year-of-schooling equivalent), (ii) certificate of primary education (corresponding to 1 ISCED1997 level and 7 US years-of-schooling equivalent), (iii) certificate of the first stage of secondary education (corresponding to 2 ISCED1997 level and 10 US year-of-schooling equivalent), (iv) diploma of secondary education including Technical, and General Secondary Education and second year of the third stage of Vocational Secondary Education (corresponding to 3 ISCED1997 levels and 13 US years-of-schooling equivalent), (v) college, university degree or higher diploma (corresponding to 5-6 ISCED1997 levels and 20 US years-of-schooling equivalent). Our data did not allow differentiate between levels 5 and 6 of ISCED classification and thus as per the analytical plan guidance, everyone with college, university degree or higher diploma was imputed at level 5 and indicated as 20 years of schooling. We cannot exclude that some of the mothers who declared to have diploma of secondary education additionally achieved a post-secondary non-tertiary certificate (eg. Certificate of third year Vocational Secondary Education and one-year programmes secondary-after-secondary) which should be classified as ISCED1997 level 4. However, attendance post-secondary courses is almost negligible in Belgium (approximately 1% according to data of 2017 from the Belgian Statistic Agency) and therefore no ISCED1997 level 4 was considered.

*Methylation measurements*

In a subset of 377 children from the ENVIRONAGE cohort cord blood DNA was extracted from buffy coats according to standard protocol. DNA concentration was measured using the Quant-IT assay from Thermo Fisher. Gel-electrophoresis was performed to assess the integrity of DNA samples. Methylation data was produced at GenomeScan in Netherland. Bisulphite conversion using 100-500 ng genomic DNA inputs was performed using the EZ DNA Methylation Gold kit (Zymo Research, Irvine, CA, USA). The converted samples (4µl) were amplified and hybridized on the Illumina HumanMethylation 850K BeadChip arrays and scanned using an Illumina iScan. Data quality was assessed using the R script MethylAid using analysis default settings and 1 sample was removed because of low quality (sample call rate <99%). DNA methylation data were preprocessed using the minfi package in R.^7^ Briefly, 4 samples were removed because of wrongly predicted sex using shinyMethyl (Fortin et al. 2014), the data were normalized using functional normalization, measurements with detection p-values >10e-16^21^ were set to missing leading to removal of 7961 probes with a call rate <95%. No additional sample had call rate <98%. For each CpG site methylation levels were expressed as beta values calculated as the ratios of intensities arising from methylated probes over those arising from the sum of methylated and unmethylated probes. Batch effect was corrected using the Combat algorithm.^22^ Data were trimmed removing the outliers as defined by observations exceeding three interquartile ranges below the first quartile or above the fourth quartile. No additional filtering was applied for the present analysis, leaving 857,898 CpGs available measured for 372 samples for the present analysis.

*Covariates*

At delivery the following information on parents and children were collected using questionnaires completed by the mothers: maternal age (years); maternal smoking status during pregnancy (any smoking in pregnancy/no smoking in pregnancy); maternal height and pre-pregnancy weight (which were used to calculate pre-pregnancy maternal BMI as the ratio of pre-pregnancy weight divided by the square of height). Sex of the child and gestational age (based on ultrasound) were derived from obstetric data.

*Cell type correction*

Cell type composition was estimated using filtered and combined reference dataset available via Bioconductor as “FlowSorted.CordBloodCombined.450 k”.^12^

*Batch correction*

Batch effect was corrected using the Combat algorithm.^22^ All the models were additionally adjusted for bead array row (used as numeric covariate).

*Exclusion criteria*

Out of the 372 samples with EPIC methylation, for the main analysis we excluded n=27 participants: 0 twins, 3 sibling pair, participants with non European ethnicity (n=2), participants that participated in the European project EXPOsOMICS (Vineis et al. 2017) due to overlap between the EXPOsOMICS analyses in this project (n=21), participants with missing information on covariates (n=1).

## EXPOsOMICS (ENVIRONAGE + RHEA + PICCOLIPIU)

*Design and study population*

Three population-based birth cohorts, ENVIRonmental influence ON AGEing in early life (ENVIRONAGE), Rhea, and Piccolipiù, were combined to conduct DNA methylation analyses with the European project EXPOsOMICS.^23^ Biospecimen from 400 children of the three cohorts (N=100 for ENVIRONAGE, N=101 for Rhea and N=99 for the Turin center of the Piccolipiù cohort) were semi-randomized on the Infinium HumanMethylation450 BeadChip array (Illumina Inc, San Diego, CA), such that the latter would incorporate proportional representations of the three cohorts and ensure that batch effects do not completely confound with biological covariates of interest.

**ENVIRONAGE:** ENVIRONAGE (ENVIRonmental influence ON early AGEing) is an ongoing population-based prospective birth cohort study that aims at exploring the human ageing and its interaction with the environment.^20^ The cohort includes more than 2000 mother-infant pairs that have been recruited at the delivery at the East-Limburg Hospital in Genk (Belgium) from February 2010 onward. Inclusion criteria were: delivery without planned caesarean section and ability to fill out a Dutch language questionnaire.

**Piccolipiù:** Piccolipiù is a multicentric Italian birth cohort set up to investigate the effects of environmental exposures, parental conditions and social factors acting during pre-natal and early post-natal life on infant and child health and development.^24^ Between 2011 and 2015, 3,358 mother-child pairs were recruited in 5 Italian centers (Turin, Trieste, Florence, Viareggio and Rome). Inclusion criteria were: delivery in one of the selected units participating at the study, maternal age older than 18 years, having a singleton pregnancy, residence in the catchment area of the maternity center, ability to fill out the informed consent and the questionnaire in Italian, having a telephone number to be reached at. A set of 99 children from the Turin centre of Piccolipiù cohort was included in the EXPOsOMICS studies.

**Rhea:**The Rhea study is a prospective mother-child cohort examining environmental exposures in the pregnancy and in early childhood in relation to child development and mother’s outcomes as well as their interaction with genetic make-up.^25^ Between 2007 and 2008, 1,458 mother-child pairs from Heraklion (Greece) were enrolled in the study during the pregnancy visits or at the delivery. Inclusion criteria were: residence in the study area, maternal age older than 16 years, having performed first antenatal visit in hospitals or private clinics in Heraklion district, and having no communication handicap.

*Ethics statement*

**ENVIRONAGE:** Ethical approval was obtained by the Ethical Committee of Hasselt University and the East-Limburg Hospital, and written informed consent was given by the parents.

**Piccolipiù:** Ethical approvals have been obtained from the Ethics committees of the Local Health Unit Roma E (management centre), of the Istituto Superiore di Sanità (National Institute of Public Health) and of each local centre. Parents provided written informed consent.

**Rhea:** Ethics Committee of the University Hospital at Heraklion approved the study protocols and written informed consent was obtained from the mothers.

Ethical approval was also obtained by the IARC Ethics Committee (IEC13-16).

*Maternal education*

**ENVIRONAGE:** Maternal education achievement was self-reported by the mothers at birth and was collapsed into the following categories: (i) no diploma (corresponding to 0 ISCED1997 level and 1 US year-of-schooling equivalent), (ii) certificate of primary education (corresponding to 1 ISCED1997 level and 7 US years-of-schooling equivalent), (iii) certificate of the first stage of secondary education (corresponding to 2 ISCED1997 level and 10 US year-of-schooling equivalent), (iv) diploma of secondary education including Technical, and General Secondary Education and second year of the third stage of Vocational Secondary Education (corresponding to 3 ISCED1997 levels and 13 US years-of-schooling equivalent), (v) college, university degree or higher diploma (corresponding to 5-6 ISCED1997 levels and 20 US years-of-schooling equivalent). Our data did not allow differentiate between levels 5 and 6 of ISCED classification and thus as per the analytical plan guidance, everyone with college, university degree or higher diploma was imputed at level 5 and indicated as 20 years of schooling. We cannot exclude that some of the mothers who declared to have diploma of secondary education additionally achieved a post-secondary non-tertiary certificate (eg. Certificate of third year Vocational Secondary Education and one-year programmes secondary-after-secondary) which should be classified as ISCED1997 level 4. However, attendance post-secondary courses is almost negligible in Belgium (approximately 1% according to data of 2017 from the Belgian Statistic Agency) and therefore no ISCED1997 level 4 was considered.

**Piccolipiù:** Maternal education achievement was self-reported by the mothers at delivery and was collapsed into the following categories: (i) no degree (corresponding to 0 ISCED1997 level and 1 US year-of-schooling equivalent), (ii) primary school degree (corresponding to 1 ISCED1997 level and 7 US year-of-schooling equivalent), (iii) lower secondary school degree (corresponding to 2 ISCED1997 level and 10 US year-of-schooling equivalent), (iv) upper secondary school degree (corresponding to 3 ISCED1997 level and 13 US year-of-schooling equivalent), (v) university degree or higher (corresponding to 5-6 ISCED1997 level and 20 US year-of-schooling equivalent). Our data did not allow differentiate between levels 5 and 6 of ISCED classification and thus as per the analytical plan guidance, everyone with university degree or higher was imputed at level 5 and indicated as 20 years of schooling. We cannot exclude that some of the mothers who declared to have an upper secondary school degree additionally achieved a post-secondary non-academic degree (eg. second level vocational course organized by Italian Regions or Higher Technical Education and Training course) which should be classified as ISCED1997 level 4. However, attendance to non-academic post-secondary courses is almost negligible in Italy (1% according to data of 2005 from the Ministry of Education, University and Research) and therefore no ISCED1997 level 4 was considered.

**Rhea:** Maternal education achievement was self-reported by the mothers at requirement (12-14th week of gestation) and was collapsed into the following categories: (i) never gone to primary school or attended primary school without completing it (corresponding to 0 ISCED1997 level and 1 US year-of-schooling equivalent), (ii) completed primary school (corresponding to 1 ISCED1997 level and 7 US years-of-schooling equivalent), (iii) completed junior high school (corresponding to 2 ISCED1997 level and 10 US years-of-schooling equivalent), (iv) completed senior high school (corresponding to 3 ISCED1997 level and 13 US years-of-schooling equivalent), (v) completed a higher education (KATEE/ SELETE) (corresponding to 4 ISCED1997 level and 20 US years-of-schooling equivalent), (vi) completed university or technological educational institute (corresponding to 5-6 ISCED1997 level and 19 US years-of-schooling equivalent).

*Methylation measurements*

For all the three cohorts, cord blood samples collected and frozen at birth at -80°C were shipped on dry ice at the International Agency for Research on Cancer of Lyon in France France, where DNA was extracted (QIAamp 96 DNA Blood Kit, Qiagen 51161), quantified (Quant-iT PicoGreen dsDNA Assay Kit, Molecular Probes P7589), and bisulfite converted (600 ng of DNA using EZ-96 DNA Methylation kit, Zymo Research D5004). DNA methylation was measured at 485,577 CpGs using the Infinium HumanMethylation450 BeadChip (Illumina Inc., San Diego, USA). The arrays were designed such that batch effects (e.g. sample position and intra- and inter-variability in arrays and chips) would not completely confound with biological covariates of interest. This design allows the retention of biological variation even after correction for technical variation. Raw intensity (.idat) files were handled in R using the minfi package^7^ to calculate the methylation level at each CpG as the beta-value (β=intensity of the methylated allele (M)/(intensity of the unmethylated allele (U) + intensity of the methylated allele (M) + 100)), and the data were exported for quality control and processing. Additionally, samples with potential gender mismatches and having > 1% of CpG sites with a detection p-value > 0.05 were removed. The remaining dataset (including 470,963 CpGs for 197 EVIRONAGE, 98 Piccolipiù and 93 Rhea samples) was normalized using the funnorm normalization of the minfi package.^7,19^ Only for Piccolipiù and Rhea cohorts, batch effect was corrected using the Combat algorithm.^22^ Additionally, data were trimmed removing the outliers using Turkey method.

*Covariates*

**ENVIRONAGE:** At delivery the following information on parents and children were collected using questionnaires completed by the mothers: maternal age (years); maternal smoking status during pregnancy (any smoking in pregnancy/no smoking in pregnancy); maternal height and pre-pregnancy weight (that were used to calculate pre-pregnancy maternal BMI as the ratio of pre-pregnancy weight divided by the square of height). Sex of the child and gestational age (based on ultrasound) were derived from obstetric data.

**Piccolipiù:** At birth information on parents and children were collected using questionnaires. Questionnaire data were used to derive: maternal BMI, calculated from self-reported pre-pregnancy weight and height; sex of the child; maternal age (years); maternal smoking status during pregnancy (any smoking in pregnancy/no smoking in pregnancy). Gestational age (weeks) was estimated based on last menstrual period or on maternity records.

**Rhea:** At the 12th week of gestation information on parents and children were collected using questionnaires. Questionnaire data were used to derive: maternal BMI, calculated using parental height and pre pregnancy weight reported by the mother; sex of the child; maternal age (years); maternal smoking status during pregnancy (any smoking in pregnancy/no smoking in pregnancy); maternal smoking status during the pregnancy (any smoking at 12th and 30th week of the pregnancy/no smoking at 12th and 30th week of the pregnancy.

*Cell type correction*

For all the three cohorts, cell type composition was estimated using filtered and combined reference dataset available via Bioconductor as “FlowSorted.CordBloodCombined.450 k”.^12^

*Batch correction*

For ENVIRONAGE batch effect was accounted by adjusting all the models for technical variables: bead array row and bisulfite conversion batch. For Piccolipiù and Rhea cohorts, batch effect was corrected using the Combat algorithm^22^ and all the models were additionally adjusted for bead array row.

*Exclusion criteria*

Subsample of ENVIR*ON*AGE, Piccolipiù and Rhea of EXPOsOMIC project includes all singleton and no siblings. For ENVIR*ON*AGE main analysis, out of the 197 samples with 450K methylation data available, we excluded 7 participants with non-European ethnicity (n=1) and participants with missing information on education (n=6). One sample missing information on covariates was excluded from main analysis out of the 98 with 450K methylation data available in Piccolipiù. One sample missing information on education was excluded from main analysis out of the 93 with 450K methylation data available in Rhea.

## Finnish Gestational Diabetes (FinnGeDi) study

*Design and study population*

This subcohort of 299 women with gestational diabetes mellitus (GDM) and 238 controls is a part of the Finnish Gestational Diabetes (FinnGeDi) case-control study including 2212 women aged 19–45 years.^26^ Women with GDM were recruited as they came to give birth, and the next consenting woman without GDM was recruited as a control. The participants came from seven delivery hospitals in Finland (Oulu, Helsinki, Jyväskylä, Pori, Kajaani, Seinäjoki and Lappeenranta) between 2/2009 and 12/2012. GDM was diagnosed by 2-hour 75 g oral glucose tolerance test (OGTT) at 5-37 weeks of gestation (24 (7) weeks, mean (SD)) where the cut-off concentrations for venous plasma glucose were ≥ 5.3 mmol/l at baseline (fasting glucose), ≥ 10.0 mmol/l at 1 h or ≥ 8.6 mmol/l at 2 h after glucose intake. GDM diagnosis was set if one or more glucose concentrations exceeded the cut-off levels. Data include cord blood DNA sample; clinical data from hospital and maternal welfare records; register data from national registers; and self-reported lifestyle, medical and family history data from questionnaires. The cohort is located at the Finnish Institute for Health and Welfare (Oulu and Helsinki, Finland).

*Ethics statement*

All the participants (mothers) provided written informed consent. The study received ethical approval from Northern Ostrobothnia Hospital District Ethical Committee 2008/43 (16.6.2008).

*Maternal education*

Information on maternal education was self-reported by mother during delivery visit using questionnaire. Original question was: What is the highest education you have completed? Response categories included nine options: 1) primary secondary school (before the Finnish school reform in the 1970s), 2) lower secondary school (before the Finnish school reform in the 1970s), 3) lower secondary (compulsory) school, 4) matriculation (upper secondary theoretical), 5) upper secondary vocational, 6) short-cycle tertiary, 7) polytechnic, 8) university and 9) none of the previous. US years of schooling were calculated in accordance with the ISCED 1997 classification equivalent: original categories 1-3 = ISCED 2 or less = 10 years, original categories 4-6 = ISCED 3 =13 years, original category 7 = ISCED 4 = 15 years and 4 = original category 8 = ISCED 5 and 6 = 20 years. Differentiation between ISCED levels 5 and 6 was not possible in our original data, therefore, 20 years of schooling were imputed for every participant who reported university level education.

*Methylation measurements*

Sub sample for the methylation study included all non-smoking women with any abnormal glucose value in OGTT and DNA samples available from both mother and offspring. Controls were eligible if they were non-smokers and confirmed as not having GDM by OGTT after 24 weeks of gestation. Mothers were intended to be matched by age and pre-pregnancy BMI to select an equal number of GDM and control women in each stratum. However, a total of 238 women were selected as controls for 298 GDM mothers because there were not enough eligible controls in the older age and BMI categories.

Methylation of genomic DNA was measured using Infinium ‘850K’ MethylationEPIC BeadChip array (Illumina, Inc., San Diego, CA, USA). Offspring leukocyte DNA sample was drawn from cord blood immediately after the birth. Samples and probes with a call rate below 95 % were excluded. Call rates were computed using a 1 % threshold on the detection p-values. Probes which aligned to multiple locations,^27^ were non-CpG or with bead counts less than 3 were excluded. Type I and type II probes were normalised using the Regression on Correlated Probes (RCP) method^28^ from the ENmix package^29^ for R.^30^

*Covariates*

Maternal data was collected from medical records, Medical Birth Register (MBR) and by using questionnaire during delivery-visit. Data on maternal smoking status during pregnancy was compiled from MBR and questionnaire and dichotomised as ‘any smoking during pregnancy’ and ‘no smoking during pregnancy’. All the mothers with DNA methylation data were non-smokers. Maternal age was assessed at delivery from MBR. Gestational age was established by first-trimester ultrasound examination. Maternal pre-pregnancy BMI was calculated using information on self-reported height and self-reported pre-pregnancy weight through questionnaire and MBR. Offspring data was collected at birth until the age of seven days from MBR.

*Cell type correction*

Cell proportions were estimated using Salas reference panel.^30^ Cell estimates included in the models were nRBC, CD8T, CD4T, NK, Bcell, Mono and Gran.

*Batch correction*

Data were normalised for possible batch effect bias using the ComBat function from the sva^31^ package for R, with batch/slide as covariate.

*Exclusion criteria*

Exclusion criteria were multifetal birth, pre-pregnancy diabetes and smoking during pregnancy. For this analysis we excluded n=41 participants: 38 with missing maternal education and 4 with missing birth length from which one was missing both, maternal education, and birth length.

## Groningen Expert Center for Kids with Obesity (GECKO)

*Design and study population*

The Groningen Expert Center for Kids with Obesity (GECKO) Drenthe cohort is a population-based prospective birth cohort study in Drenthe, a northern province in the Netherlands. All mothers of infants born between April 2006 and April 2007 were invited to participate during the third trimester of pregnancy. Of all 4,778 infants born in this period, a total of 2,874 newborns (60%) participated in the study. The GECKO Drenthe study complies with the Declaration of Helsinki. It has been approved by the Medical Ethical Committee of the University Medical Center Groningen and parents of all participants gave written informed consent. Details about this cohort have been described elsewhere.^32^

*Methylation measurements*

Within the GECKO Drenthe birth cohort, we selected 258 infants for the methylation study: 129 exposed to maternal smoking during pregnancy and 129 unexposed to both maternal and paternal smoking during pregnancy.^33^ From these 258 infants, we used DNA which was extracted from cord blood for the epigenome-wide DNA methylation analyses. To limit batch effects, we randomized all samples over the 96-well plates, based on child sex and maternal smoking status. Samples (500 ng per sample) were placed on three 96-well plates. Bisulfite conversion was performed using the EZ-96 DNA methylation kit (Zymo research Corporation, Irvine, USA). Then we used the Infinium HumanMethylation450 BeadChip (Illumina Inc., San Diego, USA) to measure the methylation level as a beta value ranging from zero (no methylation) to one (complete methylation). During the quality control, we excluded two males that clustered in the female group, based on X chromosome betas, which was probably due to maternal blood contamination. We performed Illumina-suggested background normalization, colour correction and Subset-quantile Within Array Normalization (SWAN). We excluded one sample because it did not meet the criteria of ≥99% of the CpGs with detection p value <0.05. This resulted in 129 children exposed to maternal smoking and 126 children unexposed to maternal smoking. We excluded control probes, probes on X or Y chromosomes and probes that did not meet our criteria of a detection p value of <0.05 in ≥99% of the samples, resulting in 465,891 remaining CpGs.

*Maternal Education*

Maternal education was self-reported through questionnaire. Educational categories were mapped to ISCED 1997 categories and then converted to US years of schooling. Our data did not allow differentiation between ISCED categories 5 and 6, and thus as per analysis protocol, all participants with tertiary education were assigned 20 years of schooling.

| **Levels** | **Definition** | **US years of schooling** | **GECKO Drenthe categories** | **Years of schooling codes** |
| --- | --- | --- | --- | --- |
| 0 | Pre-primary education | 1 | Geen opleiding | 1 |
| 1 | Primary education or first stage of basic education | 7 | Lager onderwijs (Basisschool) | 7 |
| 2 | Lower secondary or second stage of basic education | 10 | Lager of voorbereidend beroepsonderwijs (VMBO)  Middelbaar algemeen voortgezet onderwijs (MAVO) | 10 |
| 3 | (Upper) secondary education | 13 | Middelbaar beroepsonderwijs of beroepsbegeleidend onderwijs (MBO)  Hoger algemeen en/of voorbereidend wetenschappelijk onderwijs (HAVO/VWO) | 13 |
| 4 | Post-secondary non-tertiary education | 15 | N/A | N/A |
| 5 | First stage of tertiary education (not leading directly to an advanced research qualification) | 19 | Hoger beroepsonderwijs (HBO)  Wetenschappelijk onderwijs (Universiteit) | 20 |
| 6 | Second stage of tertiary education (leading to an advanced research qualification, e.g. a Ph.D.) | 22 |  |  |

*Covariates*

We adjusted for child sex, maternal age (based on self-report), maternal BMI (based on self-reported pre-pregnancy height and weight), and gestational age (reported by midwives). Maternal smoking (based on self-report and cross-validated with midwife reports) was a selection factor, and thus was adjusted for in each model.

*Cell type correction*

Blood cell subtype were estimated by the Salas method and adjusted for in each model.

*Batch effects*

Batch effects were accounted for by including array and plate number of each Infinium HumanMethylation450 BeadChip (Illumina Inc., San Diego, USA) in each model.

*Exclusion criteria*

In the methylation subset of the GECKO Drenthe cohort we excluded infants born preterm (<37 weeks), and whose mothers experienced pre-eclampsia or diabetes during pregnancy, this information was obtained from questionnaires completed by the mothers shortly after delivery and combined with medical birth records.

## Generation R Study (GenR)

*Design and study population*

The Generation R Study is a population-based prospective cohort study from fetal life onwards. Pregnant women with an expected delivery date between April 2002 and January 2006 living in the city of Rotterdam were eligible to participate.^34^ In a subgroup of the 9,901 live-born participating children from European ancestry we measured genome-wide DNA methylation in cord blood (n=1396) and/or in venous blood at childhood age six years (n=493).^35^ For the current analyses, we included only one child for each of 15 sibling pairs, based on completeness of data and if equal, randomly. The Generation R Study was approved by the medical ethical committee of Erasmus MC, University Medical Center Rotterdam and written consent was obtained for all participants. This study included children with information on maternal education during pregnancy and DNA methylation at birth (cord blood) or in childhood (age 6 years) and complete data on the covariates.

*Maternal Education*

Information on the highest completed level of maternal education was obtained from questionnaires at intake, which was generally in early pregnancy. Categories of education were recoded for this analysis into: Primary education (coded as 7 years of schooling), lower secondary education (coded as 10 years of schooling), upper secondary education (coded as 13 years of schooling), higher education phase 1 (coded as 19 years of schooling) and higher education phase 2 (coded as 22 years of schooling). The categories of pre-primary education and post-secondary non-tertiary education were not present in the Generation R participants with DNA methylation data.

*Methylation measurements*

We used the salting-out method to extract DNA from cord or venous blood samples. Five hundred nanograms of DNA were bisulfite converted using the EZ-96 DNA Methylation kit (Shallow) (Zymo Research Corporation, Irvine, USA). Samples were plated onto 96-well plates in no specific order. Samples were processed with the Illumina Infinium HumanMethylation450 BeadChip (Illumina Inc., San Diego, USA). Quality control and normalization were performed using the CPACOR workflow.^21^ Probes with a detection *p* value ≥1E-16 were set to missing. Intensity values were stratified by autosomal and non-autosomal probes and quantile normalized for each of the six probe type categories separately: type II red/green, type I methylated red/green and type I unmethylated red/green. Arrays with observed technical problems such as failed bisulfite conversion, hybridization or extension, as well as arrays with a mismatch between sex of the proband and sex determined by the chr X and Y probe intensities were removed from subsequent analyses. Additionally, only arrays with a call rate > 95% per sample were processed further. Probes on the X and Y chromosomes were excluded from the dataset. The final dataset contained information on 458,563 CpGs.

*Covariates*

As described previously, information on maternal covariates was obtained from questionnaires sent out at enrollment.^1^ Maternal age was collected at enrollment. Pre-pregnancy body mass index was calculated from height at enrollment and self-reported pre-pregnancy weight. Smoking during pregnancy was dichotomized into no smoking or quit before second trimester versus sustained smoking. Information on gestational age, child sex and date of birth was obtained from midwife and hospital records. We calculated child age from date of birth. We measured height and weight without shoes or heavy clothing, from which we calculated body mass index (kg/m^2^) when children visited our research center at school-age. All participants were of European ancestry. Cell type proportions were estimated from the methylation data using a cord blood specific reference set in the “FlowSorted.CordBlood.Combined.450K” Bioconductor package and the reference-based Houseman method for cord and child blood, respectively.^12,36^ We adjusted for batch effects by adding plate number as a covariate.

## HAVEN

*Design and study population*

The HAVEN study, a Dutch acronym for the study of heart anomalies and the role of genetic and nutritional factors, is a case–control study designed to investigate determinants in the pathogenesis and prevention of CHD and described in detail before. DNA methylaton data was published before. In short, participants were children born in the western part of The Netherlands between January 2002 and June 2007. During a standardized hospital visit around 17 months of age, venous blood samples were drawn from the children and the questionnaires filled out by the mother at home were checked for completeness and consistency. DNA methylation was measured for children born with a congenital heart defect (N=84) and children born without congenital heart defects (N=196).

*Ethics statement*

The study was approved by the Central Committee for Human Research in The Hague, The Netherlands, and by the Institutional Review Boards (Medical Ethics Committees) of the Erasmus MC University Medical Center in Rotterdam, Leiden University Medical Center in Leiden, VU University Medical Center and Academic Medical Center in Amsterdam, The Netherlands. All parents gave written informed consent on behalf of their participating child.

*Maternal education*

Information on maternal education was self-reported by mothers using questionnaire at roughly 17 months post-partum. The question asked was ‘what is your highest education level’ and response categories included: primary education only, lower vocational eduction (LBO), lower high school (MAVO), higher high school (HAVO or VWO), higher vocational eduction (HBO), university eduction, other (which was then accompanied by written text and interpreted by staff). These categories were recoded in accordance with the ISCED 1997 classification equivalent to US years of schooling in the study.

*Methylation measurements*

Samples were randomly allocated to 96 well plates for bisulfite treatment and measured on an Illumina 450k array following the standard Illumina protocol at the the Erasmus MC University Medical Center array facility. Quality control of the 450K array measurements was performed using the *MethylAid* package and the absence of sample swaps and mixtures was checked using the omicsPrint package using the signature of sex differences and several hundreds of SNPs measured on the array. Pre-processing was performed using the DNAmArray package on github and normalization was performed with *Functional Normalization* as implemented in the *Minfi* package using 5 principal components. No arrays failed the array quality control measures, but both before and after normalization 5 samples clustered from the entire cohort and these were removed prior to final normalization.

*Covariates*

Maternal data was collected during postal questionnaire and clinical examinations. Data on maternal smoking status during pregnancy was self-reported as ‘smoking during pregnancy’. ‘stopped smoking during pregnancy’ (and week of cessation) and ‘no smoking’. Recoding was done as instructions. Maternal age at pregnancy was taken from clinical records. Gestational age was established by last menstrual period of the mother and cross-evaluated through ultrasound examination. Maternal pre-pregnancy BMI was taken from hospital records and was self-recorded through questionnaire. Offspring data was collected during follow-up at ~17 months years of age.

*Cell types*

Cell types were inputed by the Houseman method^3^.

*Batch correction*

Two different lots of extension dye were used during the measurement of the arrays, for which *Functional Normalization* proved inadequate to completely remove, and we adjusted for this in the model. In the model we corrected for the unique combination of bisulfite plate and scan batch (factorial covariate) and the height of the sample on the micro-array glass slide (continuous variable from 1-6) as they were the strongest technical effects identified on the DNA methylation data genome-wide.

*Exclusion criteria*

We excluded the children born with a congenital heart defect (N=84). In addition, only the control children from Dutch descent and without missing data for the covariates required of the meta-analysis were used (N=138, so N=58 excluded).

## The Healthy Start Study

*Design and study population*

The Healthy Start study is an ongoing longitudinal cohort study that enrolled pregnant women from outpatient obstetrics clinics at the University of Colorado Hospital (2009-2014). Eligible women had singleton pregnancies with no chronic medical conditions and no history of stillbirth or live birth prior to 25 weeks of gestation. Exclusion criteria are described in greater detail in Starling et al.^37^ The study protocol was reviewed and approved by the Colorado Multiple Institutional Review Board. All participants provided written informed consent.

*Maternal Education*

At the time of the first study visit (median 17 weeks of gestation) participants were asked to self-report their highest educational level attained by selecting one of the following categories: less than 12^th^ grade, high school degree or GED, some college or associate’s degree, four years of college (BA, BS), or graduate degree (Master’s, PhD). Each of these categories was converted into a numeric years of schooling variable as follows: less than 12^th^ grade, 9 years; high school degree or GED, 13 years; some college or associate’s degree, 15 years; four years of college, 19 years; graduate degree, 22 years.

*Methylation measurements*

DNA methylation was evaluated in cord blood using the Illumina Infinium HumanMethylation450 array in a convenience sample of 600 participants with available stored cord blood samples. We performed quality control checks and removed 587 probes with high detection p-value and 664 probes with low beadcount in >5% of samples. We used the preprocessQuantile function in Minfi for normalization. The final number of CpGs analyzed was 484,261.

*Covariates*

All models were stratified by category of maternal self-reported race/ethnicity: non-Hispanic white, Hispanic, or African-American. Models included adjustment for the following covariates: child sex (obtained from maternal report or the delivery medical record), maternal age (self-reported at study enrolment), maternal BMI prior to pregnancy (calculated based on measured height and weight prior to pregnancy obtained from the medical record or self-reported at the first study visit), maternal smoking (self-reported at three time points during pregnancy and classified as no smoking, quit early in pregnancy, or continued through pregnancy), and infant gestational age at birth (obtained from the medical record). We excluded 75 participants who were missing data on smoking during pregnancy.

*Cell type correction*

We estimated proportions of the following seven cell types in cord blood using a combined cord blood reference data set in the R package FlowSorted.CordBloodCombined.450k as described in Gervin et al: B cells, CD4+ T cells, CD8+ T cells, granulocytes, monocytes, NK cells, nucleated red blood cells.^12^

*Batch correction*

We used ComBat for batch correction.

*Exclusion criteria*

From 600 participants with DNA methylation data available, we excluded n=125 participants: 6 participants with a sibling enrolled in the study, 39 participants with a race/ethnicity other than non-Hispanic white, Hispanic, or African-American, 75 participants missing information on smoking during pregnancy, and 5 participants for whom the self-reported sex did not match the methylation-predicted sex. This led to a total of 475 participants in the analysis, of which 259 were non-Hispanic white, 132 were Hispanic, and 84 were African-American.

## INfancia y Medio Ambiente (INMA) - Sabadell

*Design and study population*

The present study used data from participants recruited between 2003 and 2008 in the de novo cohort sited in Sabadell of the INfancia y Medio Ambiente (INMA) Project,^38^ a population-based mother–child cohort study in Spain. The current project uses data from European ancestry children from the Sabadell subcohort. Study website: http://www.proyectoinma.org/.

This study included children with maternal education at pregnancy, blood DNA methylation measurements at any time point, and complete covariate data. The number of participants with both DNA methylation and phenotype data is 377 (for cord blood study) and 197 (for whole blood at 4 years of age study).

Ethics statement

The study has been approved by Ethical Committee of each participating center and written consent was obtained from participating parents.

*Maternal education*

Maternal education was used as continuous variable. We used equivalent school years and not categories.

This variable was coded as:

You can’t read nor write -> 0yrs;

Without education or unfinished primary school -> 7yrs;

Primary school (EGB, primary school, ESO) -> 10yrs;

Secondary school (BUP, COU, FP) -> 13yrs;

University studies -> 20yrs.

*DNA Methylation measurements* *(and batch correction)*

Cord blood and whole blood at age 4y was extracted using the Chemagen kit (Perkin Elmer). DNA concentration was determined by NanoDrop spectrophotometer (Thermo Scientific) and with the Quant-iT PicoGreen dsDNA Assay Kit (Life Technologies). Methylation data was produced in two different laboratories as part of two different projects: in the Genome Analysis Facility of the University Medical Center Groningen (UMCG) in Holland, and in the Bellvitge Biomedical Research Institute (IDIBELL, Barcelona). Both laboratories used the recommended Illumina protocol for the Infinium HumanMethylation450 beadchip. Briefly, 500 ng of DNA was bisulfite-converted using the EZ 96-DNA methylation kit following the manufacturer’s standard protocol, and DNA methylation measured using the Illumina Infinium HumanMethylation450 beadchip. DNA methylation data were preprocessed using the minfi package.^7^

A series of steps were completed for quality control and data analysis. The first step was low quality sample removal. First, 2 samples with bad overall quality or with low detection p-value according to the output of the MethylAid package were removed. Then, we removed 3 samples whose sex was wrongly predicted using shinyMethyl. Following guidelines of Lehne work, we increased the stringency of the detection p-value threshold to 10-16 and we filtered 18 samples with a call rate lower than 98%. The second step was normalizing data with functional normalization. Correlation between SNP in replicates samples was checked and probes not measuring SNPs were discarded. 7,136 probes with a call rate lower than 95% were also removed. Probes in sexual chromosomes, crosshibridizing or containing SNPs were flagged but not removed at this point. ComBat was applied to remove batch effect. Finally, duplicated samples were removed.^22,39^

*Covariates*

Information on maternal age was collected by questionnaire at enrolment (week 12 of pregnancy). Maternal height was measured, and maternal pre-pregnancy weight was reported by the mother during the first prenatal visit. From these variables maternal pre-pregnancy BMI was calculated. Pregnant women were asked about maternal smoking at week 12 and 32 of pregnancy. The variable was divided in two categories; no smoking or smoking at 1rst trimester only, and sustained smoking. Child sex was abstracted from clinical records. Gestational age at blood sampling was calculated based on last menstrual period (LMP) reported at recruitment and confirmed using estimates based on ultrasound examina­tion in the 12th week of gestation. When the difference between the LMP reported at recruitment and estimated from the ultra­sound was ≥ 7 days (n=91; 16%), we esti­mated LMP using a quadratic regression formula. Maternal age at birth (years) was also recorded.

Variables applicable only for whole blood analysis: child BMI (kg/m2), and child age at blood sampling (years).

Selection factor: non-applicable.

All individuals are classified as Europeans taking into account ethnic origin and country of origin of both parents, thus models were not adjusted for ancestry.

*Cell type correction*

Cell type proportions were estimated from the methylation data using:

-Cord blood reference panel with 7 cell types by Gervin et al.

-Whole blood reference panel with 6 cell types by Houseman et al.

*Batch correction*

No batch variable was used because ComBat method was applied to control for the effect of laboratory and other potential technical variables.

*Exclusion criteria*

Non-European ancestry children were excluded from the analyses as well as siblings and twins.

## Lifestyle and environmental factors and their Influence on Newborns Allergy risk (LINA)

*Design and study population:* The LINA study is a running prospective birth cohort study conducted by the Helmholtz Centre for Environmental Research-UFZ in Leipzig, Germany, with the aim of entangling the impact of environmental factors on the maturation of children’s immune system and the development of allergic diseases, obesity and behavioral disorders.^40^ For this study, 629 mother-child pairs were recruited from 2006 until 2008 in the German city of Leipzig. Pregnant women were invited to participate, and after informed consent, maternal blood and urine samples as well as questionnaire data were collected around the 34^th^ week of gestation. Since birth, children as well as mothers are followed up annually by standardized questionnaires and clinical visits, including blood, urine, and feces collection. Several immune, metabolome and gene expression analyses have been performed using both maternal and child blood.^41,42^ All children with DNA methylation data were of European ancestry. For the current study, methylation analyses were performed in 472 cord blood samples.

*Ethics statement:* All participants gave written informed consent. The LINA study was approved by the Institutional Review Board of the University of Leipzig and the Saxonian Board of Physicians (046-2006, 160-2008, 160b/2008, 144-10-31052010, 113-11-18042011, 206-12-02072012, 169/13-ff, 150/14-ff, EK-allg-28/14-1).

*Maternal education:* Maternal education was based on self-reported level of education during pregnancy via questionnaire. The measure was based on German Educational system. Which is the highest school level you have: less than 9 years, 9 years (“Hauptschulabschluss”), 10 years (“Mittlere Reife”), 12 years or more (“(Fach-)Hochschulreife”). In addition we asked for the highest educational level: apprenticeship (2-3y), college (2-3y), master school (2-3 y), and university degree (4-5y). These categories were recoded in accordance with the ISCED 1997 classification equivalent to US years of schooling in the study. Our data did not allow differentiating between levels 5 and 6 of ISCED classification and thus the 6^th^ level was encoded manually from those participants with level 5 and a PhD degree in their names.

*DNA methylation measurements:* Genomic DNA was isolated from cord blood samples using the QIAmp DNA Blood Mini Kit (Qiagen, Hilden, Germany) followed by bisulfite conversion using the EZ-96 DNA Methylation Kit (Zymo Research Corporation, Orange, USA) according to manufacturer recommendations. All samples subsequently subjected to DNA methylation analyses (*n* = 472) passed initial quality control checks. A genome-wide DNA methylation screen was performed based on the Infinium HumanMethylation450 BeadChip (Illumina, San Diego, USA) array. Data were normalized using the SWAN (subset-quantile within array normalization) method of the minfi R package. DNA methylation values, described as beta (β) values, were recorded for each locus in each sample. The β values represents the ratio of methylated signal relative to the sum of methylated and unmethylated signal measured per CpG.

*Covariates*

Maternal data was collected during pregnancy (34^th^-36^th^ week of gestation) and annually thereafter by using questionnaires and clinical examinations. Mothers reported whether they had smoked during pregnancy, classified as ‘never’, ‘occasionally’, and ‘daily’. In the current analyses, we used a dichotomous variable that distinguished ‘sustained smokers during pregnancy’ vs ‘non-smokers/quit when pregnancy was known’. Maternal age was assessed at antenatal visit (34^th^ week of gestation). Gestational age was established by last menstrual period of the mother and cross-evaluated through ultrasound examination. Offspring data was collected during annually follow-ups via questionnaires self-reported by the mothers. Height and weight were measured during clinical examination and converted to BMI as kg/m^2^.

*Cell type correction:* Cell counts estimated using the Salas cord blood reference panel^12^ were included as covariates.

*Batch correction:* Adjustment for batch effects was done by including sample plate number as a covariate.

## The Norwegian Mother, Father and Child Cohort Study (MoBa)

*Design and study population*

The Norwegian Mother, Father and Child Cohort Study (MoBa) recruited pregnant women from July 1999 to December 2008.^43–45^ At weeks 17 and 30, women completed questionnaires about general demographic information and previous and present health problems and exposures. MoBa1 is a substudy of MoBa. The substudy was a cohort random sample and asthma cases at age three years.^46,47^ Offsprings in this substudy were born in 2002 to 2004. MoBa2 is a non-overlapping subset selected from MoBa.^46,48^ MoBa2 included a cohort random sample and asthma cases at age seven years and non-asthmatic controls, as well as an additional ~200 subjects who had plasma folate measured. Offspring in this subset were born in 2000 to 2005. MoBa3 was designed to evaluate the association between differential cord blood DNA methylation and later childhood cancer status. MoBa mothers provided written informed consent. Methylation measurements for MoBa3 were made at IARC. Years of birth for children in MoBa3 were 2000-2008.

*Ethics statement*

All women who participated in MoBa provided written informed consent. MoBa1 and MoBa2 were approved by the Regional Committee for Ethics in Medical Research in Norway and the Institutional Review Board of the National Institute of Environmental Health Sciences in the USA. Previous MoBa1 and MoBa2 publications were based on data release version 5. These analyses were analyzed using data release version 12. The establishment and data collection in MoBa has obtained a license from the Norwegian Data Inspectorate and approval from The Regional Committee for Medical Research Ethics, Norway. Ethical approval was also obtained by the IARC Ethics Committee (IEC11-23).

*Maternal education (same for MOBA1,2 and 3)*

Women reported the highest level of education completed and if applicable, current studies. We converted their highest level of completed education to the ISCED classification 1997 and respective U.S. years of schooling. For women missing highest level of education completed, we “rounded down” from their current studies. For example, if women reported currently in 3-year high school general studies or junior college, then they completed 1-2 year of high school. The table below details how the responses in MoBa were classified.

| MoBa response | ISCED classification | U.S years of schooling |
| --- | --- | --- |
| 9-year secondary school | Lower secondary or second stage of basic education | 10 |
| 1-2 year high school | (Upper) secondary education | 13 |
| Technical high school | Post-secondary non-tertiary education | 15 |
| 3-year high school general studies or junior college | Post-secondary non-tertiary education | 15 |
| Regional technical college, 4-year university degree | First stage of tertiary education (not leading directly to an advanced research qualification) | 19 |
| University, technical college, more than 4 years | Unable to differentiate between first and second stage tertiary education | 20 |

*Methylation measurements*

Methylation in cord blood was measured in MoBa1 first.^49^ The same laboratory measured DNA methylation in cord blood in MoBa2 later.^48^

Details of how DNA methylation were measured and the quality control procedure for MoBa1 and MoBa2 have been previously described.^48^ The same procedures were applied to both studies. Briefly, samples of umbilical cord blood were collected at birth and stored at -80°C. DNA was bisulfite converted using the EZ-96 DNA Methylation kit (Zymo Research Corporation, Irvine, CA). DNA methylation was assessed at 485,577 CpGs using Illumina’s Infinium HumanMethylation450 BeadChip.^50^ We used the *minfi* package in R to read the .idat files and calculate the beta methylation values at each CpG:

$$\beta=\frac{intensity of the methylated allele (M)}{intensity of the unmethylated allele (U) + intensity of the methylated allele (M) + 100}$$

Quality control procedures were applied on the beta methylation values. We excluded 65 control probes, probes on the X chromosome (# CpGs =11,230) and probes on the Y chromosome (# CpGs =416). CpGs missing >10% of methylation values were removed (20 CpGs in MoBa1, 0 CpGs in MoBa2). We excluded samples identified by Illumina to have failed or those with an average detection p-value <0.05 across all probes (49 in MoBa1, 35 in MoBa2), as well as samples with sex mismatches (13 in MoBa1, 8 in MoBa2). We used the beta mixture quantile (BMIQ) to normalize the data^51^ and used ComBat from the *sva* package in R for batch correction.^22^ Extreme beta methylation values (defined as greater or less than three times the interquartile range) were set to missing.

1,068 samples passed quality control in MoBa1. However, in this data release, 11 participants dropped out of the MoBa1, leaving 1,057 with QC methylation data. In MoBa2, 685 samples passed quality control. 473,844 CpGs were analyzed in MoBa1 and 473,748 CpGs were analyzed in MoBa2.

For MoBa3 samples, the bisulfite conversion and methylation measurements (Illumina’s Infinium HumanMethylation450 BeadChip) were performed by the Epigenetics Group at IARC (Lyon, France). Methylation features were filtered from (i) cross-reactive probes, (ii) probes mapping to sex chromosomes and (iii) probes overlapping with a known single nucleotide polymorphism (SNP) with an allele frequency of at least 5% in the overall population (all ethnic groups), resulting in the exclusion of 36 231 probes. Data quality was further assessed using box plots for the distribution of methylated and unmethylated signals, and multidimensional scaling plots and unsupervised clustering were used to check for sample outliers. After background correction and color-bias adjustment, type I and type II probe distributions were aligned using the intra-sample BMIQ normalization18 from the watermelon package. After quality control, the sample size for MoBa3 was 253.

*Covariates*

MoBa participants were linked to the Medical Birth Registry of Norway to collect information on maternal age at delivery, infant sex, and gestational age. Maternal pre-pregnancy body mass index was calculated from self-reported weight and height collected at the 17^th^ week of gestation. Maternal smoking status was determined based on measured cotinine levels at the 17^th^ week of gestation and self-reported smoking information at 17^th^ week of gestation, 30^rd^ week of gestation, and 6^th^ month after birth. All models were also adjusted for the selection factor. In MoBa1, the selection factor was asthma status at 3 years old (yes/no). In MoBa2, participants were selected into the study because of one of the following three groups: asthma status at 7 years old (yes/no) or had folate measured regardless of asthma status. In MOBA 3, the selection factor was later childhood cancer, with age-matched controls in the ratio 1:2 (case:control).

*Cell type correction (same for MOBA1,2 and 3)*

Potential confounding effects of blood cell subtypes were estimated by the Houseman method^52^ with the Bakulski reference panel.^53^

*Batch correction*

We used ComBat from the *sva* package in R for batch correction.^22^

*Exclusion criteria*

In MoBa1, 29 were missing information on maternal education and 38 were missing covariate information. In MoBa2, 22 were missing information on maternal education and 29 were missing covariate information. Genotype data were measured using Illumina HumanCore. We ran principal components to identify and exclude samples that were ancestry outliers (6 in MoBa1, 2 in MoBa2). This study included newborns with cord blood DNA methylation measurements, maternal education data and covariate data (N=984 from MoBa1; N=632 from MoBa2). Each dataset was analysed separately.

## Newborn Epigenetics STudy (NEST)

*Design and study population*

NEST (Newborn Epigenetics STudy) is a multiethnic prospective study of women and their children designed to identify the association between early exposures and changes in infantile epigenetic profile that may influence chronic disease susceptibility later in their lives.^54,55^ Pregnant women who were 18 years or older, English speaking and who intended to use obstetrics facilities within the Duke Obstetrics or Durham Regional Hospitals as their prenatal clinics were recruited to the study between April 2005 to July 2009. Gestational age at enrolment ranged from 6 to 42 weeks (median 30 weeks). Women who were current smokers during their pregnancy were specially targeted for a portion of the study. Of the 1101 women who met eligibility criteria, 895 (81%) were enrolled. Umbilical cord blood was collected from 741 infants born to the recruited mothers. Written informed consent was obtained from all participants. The Duke Institutional Review Board approved the study. The current analysis was limited to 327 infants with covariate and 450k methylation data.

*Maternal Education*

Maternal educational attainment was reported by the mom on the baseline questionnaire with a response to the following question: “What is the highest grade or year of school you have completed?” (Less than high school, high school graduate/GED, some college, college graduate, or graduate education). These categories were recoded to 10, 13, 15, 17, and 20 respectively in accordance with the ISCED 1997 classification equivalent to US years of schooling in the study.

*Methylation measurements*

Genomic DNA from buffy coat specimens was extracted from umbilical cord blood using Puregene Reagents (Qiagen, Valencia, CA). Extracted DNA was subjected to bisulfite conversion using the EZ-96 DNA Methylation Kit (Zymo Research Corporation) and DNA methylation was measured at 485,577 CpGs using Illumina Infinium HumanMethylation450 BeadChip (Illumina Inc., San Diego, USA. Methylation levels at each CpG (beta values) were calculated using Illumina’s GenomeStudio Methylation module version 1.0 (Illumina Inc.). Probe and sample-specific quality control was performed in the NEST cohort using a similar approach to MoBa1 and MoBa2 cohorts. Specifically, control probes (N=65) and probes on X (N=11 230) and Y (N=416) chromosomes were excluded as well as CpGs missing > 10% of methylation data. Samples were removed if i) indicated to have failed by Illumina pre-processing, ii) the average detection *P*-value across all probes were < 0.05 or iii) there was discordance between the reported sex and predicted sex. Bias arising from the two different probe designs were corrected by applying the intra-array normalization strategy Beta Mixture Quantile dilation (BMIQ).^51^

*Covariates*

Data on gestational age at delivery (continuous in weeks) and child sex were collected from the medical records at delivery. Maternal age at delivery was calculated from the maternal date of birth and the date of delivery. Maternal pre-pregnancy weight and height were self-reported by the mother as part of a standardized questionnaire completed at enrolment. BMI was calculated as pre-pregnancy weight in kilograms divided by height in square meters (kg/m2). Information on maternal smoking during pregnancy was based on self-reported questionnaire data. Women were first asked if they ever smoked and whether they were current smokers by responding to questions “Have you ever smoked 100 cigarettes or more in your lifetime?” (Yes/ No), followed by, “Do you smoke now?” (Yes/No). To determine the timing of cigarette smoking exposure to the offspring, women who reported being smokers were then asked to respond to the question, “Did you smoke anytime in the year before you found out you were pregnant?” (Yes/ No). Women also responded to the question “After you found out you were pregnant, which of the following best describes your behavior?” The four possible responses were, “I continue to smoke,” or “I stopped during the first/second/third trimester.” From these responses, three categories of maternal cigarette smoking were created as follows: (a) “smokers during pregnancy” were women who reported having ever smoked 100 cigarettes or more and smoking past early pregnancy (i.e. sustained smoking); (b) “quitters during pregnancy” were women who reported having ever smoked 100 cigarettes or more, smoking during the year of pregnancy, and stopping smoking during early pregnancy; (c) “non-smokers during pregnancy” were women who reported never smoking during the pregnancy.

*Cell type correction*

Cell type correction was completed using the Salas reference set (4).

*Batch correction*

The Empirical Bayes method via *ComBat* was applied for batch correction using the *sva* package in *R.*^22^

*Exclusion criteria*

For the main analysis we excluded n=100 participants: twins (n=27), sibling pair (n=1), race other than African-American or Caucasian (n = 26), missing maternal education (n=3) and missing

## The Northern Finland Birth Cohort (NFBC1986)

*Design and study population*

The Northern Finland Birth Cohort 1986 consists of 99% of all children, who were born in the provinces of Oulu and Lapland in Northern Finland between 1 July 1985 and 30 June 1986. 9,203 live-born individuals entered the study(University of Oulu, 1986).^56^ At the age of 16, the subjects living in the original target area or in the capital area (n=9,215) were invited to participate in a follow-up study including a clinical examination. 7344 participants attend the study in year 2001/2002, of which 5654 completed the postal questionnaire, the clinical examination and provided a blood sample^57^. DNA was extracted from all 5654 blood samples. An informed consent for the use of the data including DNA was obtained from all subjects. DNA methylation was recoded on Illumina HumanMethlation450K array for 566 randomly selected subjects. 24 technical replicates were excluded. 18 samples did not reach a call rate of >95% applying a detection P-value filter of 10^-16^. We excluded 7 samples with gender inconsistency, no sample was outlying from the overall data structure (1st PC score of the DNA methylation values outside mean +/- 4SD). DNA methylation data of 517 samples with 466290 autosomal probes (call rate filter 95%) each were used for this analysis.

*Ethics statement*

All the participants included in this study provided written informed consent. The study received ethical approval from Northern Ostrobothnia Hospital District Ethical Committee 108/2017 (15.1.2018).

*Maternal education*

Information on maternal education was self-reported by mother during pregnancy using questionnaire. The measure was based on Finnish Educational system. The question asked was ‘what is your education level’ and response categories included following: 1) less than 6 years primary school, 2) 7-8 years primary school, 3) 9-10 years primary school, 4) Vocational school or college 6-12 months, 5) Vocational school or college >1 year, 6) Matriculation, no vocational schooling, 7) Matriculation + college, 8) matriculation, university studies not finished, 9) university degree. These categories were recoded in accordance with the ISCED 1997 classification equivalent to US years of schooling in the study. Our data did not allow differentiate between levels 5 and 6 of ISCED classification and thus as per the analytical plan guidance, everyone with tertiary level education was imputed at level 5 and indicated as 20 years of schooling.

*Methylation measurements*

Methylation of genomic DNA was quantified using the Illumina HumanMethylation450 array according to manufacturer’s instructions. Bisulfite conversion of genomic DNA was performed using the EZ DNA methylation kit according to manufacturer's instructions (Zymo Research, Orange, CA).

*Covariates*

Maternal data was collected during antenatal visits using postal questionnaire and clinical examinations. Data on maternal smoking status during pregnancy was self-reported and dichotomised as ‘any smoking during pregnancy’ and ‘no smoking’. Maternal age was assessed at first antenatal visit. Gestational age was established by last menstrual period of the mother and cross-evaluated through ultrasound examination. Maternal pre-pregnancy BMI was calculated using information on height recorded at the first antenatal visit weight before pregnancy was self-recorded through questionnaire. Height and weight were measured to an accuracy of 0.1 cm and 0.1 kg and were converted into pre-pregnancy BMI. Offspring data was collected during follow-up at 16 years of age and their smoking status, age and gender were self-reported in questionnaire. Height and weight were measured during clinical examination and converted to BMI as kg/m^2^.

*Cell type correction*

Potential confounding effects of blood cell subtypes were estimated by the Houseman method.^52^

*Batch correction*

To account for batch effects in the data, principal component analysis was carried out for array control probes, and the first 30 principal components were included in the regression model as technical covariates.

*Exclusion criteria*

NFBC1986: For the main analysis we excluded n=51 participants: 0 twins, and participants with missing information on methylation (n=51).

Postpartum Outcomes in mothers with Gestational diabetes and their Offspring (POGO)

The POGO study is a cross-sectional study of mother-child pairs in Germany.^58^ The primary objective is to investigate potential mechanisms and pathways leading to the development of type 2 diabetes in women with gestational diabetes (GDM), and to the development of obesity and metabolic disorders in offspring exposed to GDM during fetal life. Mothers with or without GDM and their offspring were invited to participate in the POGO study within 1-10 years after child's delivery (delivery between 1998-2009). GDM was diagnosed according to the German Diabetes Association guidelines from 2001, defining GDM if 2 values of a 75g oral glucose tolerance test (oGTT) exceeded the following thresholds: fasting plasma glucose value > 95mg/dL (5.3mmol/L), 1-hour glucose value > 180mg/dL (10mmol/L) and/or 2-hour glucose value > 155mg/dL (5.3mmol/L). Women with GDM received dietary counseling and treatment of GDM (diet or insulin) with repeated follow-up visits until delivery according to the guidelines of the German Diabetes Association. In total, n = 155 families (n=382 mothers and their offspring) attended one clinical visit at the clinical study center of the institute of diabetes research at which a variety of demographic/socioeconomic, anthropometric, metabolic, clinical data and blood samples have been collected.

*Ethics statement*

The study protocol was approved by the Ethical Committee of the Technical University, Munich (No. 2937). All participants gave written informed consent; for the children informed consent was obtained from both parents.

*Maternal Education*

Information on maternal education was self-reported by the mother during the postpartum clinical study visit using a standardized socio-demographic questionnaire. The measure was based on German Educational system. The question asked the highest educational level of both parents including the highest school graduation and the type of completed/ongoing vocational training (if any). First, subjects were asked “what is your highest school graduation” with corresponding potential answers: Secondary school, middle/business school, college, A level, other graduation, left school without graduation, not yet finished graduation. Second, subjects were asked “what vocational training do you have” with the following response options: apprenticeship, college, vocational school, university, no vocational training, still in vocational training. Based on these information, the highest education level was allocated to the ISCED 1997 categories using Germany-specific references and categorized in US school years equivalents according to the analysis plan. In the POGO study, no discrimination was possible between education levels 5 and 6, therefore 20 US school years were used for level 5 as instructed in the analysis plan.

*Methylation measurements*

For the present analysis, complete data were available from n = 49 mother-child pairs. In accordance with the analysis plan, only one child per mother was used, which was selected based on completeness of data or randomly, if siblings had similar available data. During the clinical visit at the study center, whole blood was collected from the children according to standardized procedures. DNA was isolated subsequently from whole blood using the salting out method and frozen at -80°C. Purification of DNA was done using the Genomic DNA Clean & Concentrator kit (Zymo Research) followed by DNA quantification using the Quant-iT PicoGreen dsDNA assay kit (ThermoFisher). DNA quality was assessed using a NanoDrop (ThermoFisher). Methylome analyses and data pre-processing were performed at Imperial College London, Department of Genomics of Common Disease, as part of the DynaHealth project. 800 ng of DNA was bisulfite-converted using the EZ-96 DNA Methylation Kit (Zymo Research) according to manufacturer’s protocol with alternative incubation conditions as recommended by Illumina. After conversion, genome-wide DNA methylation of 854,307 CpG sites were measured using the Illumina MethylationEPIC Beadchip (850K) according to manufacturer’s instructions. The beadchips were imaged by an Illumina iScan instrument using the scanning software v3.3.29. Initial quality assessment was performed using GenomeStudio (v2011.1, using the methylation module v1.9.0). All samples underwent Illumina normalization as well as background correction. Probes were excluded from analyses if more than 95% of values were above the detection P-value threshold (p>1xE-16). Furthermore, samples with more than 95% of probes above the detection P-value threshold (p>1xE-16) were excluded. We used the functional normalization approach described by Fortin et al.^3^, using the first 10 PCs of the Illumina EPIC array control probes, to normalize the data. In summary, 838,415 CpGs were used for the analysis.

*Covariates*

In line with the analysis plan, the following covariates were included in the models in addition to maternal education: maternal age, smoking during pregnancy, gestational age, pre-pregnancy maternal BMI, child BMI, sex and age. None of the children smoke, therefore child smoking was not included into the analyses. The variables maternal age, smoking during pregnancy, and child age were collected by self-report. Maternal pre-pregnancy BMI, gestational age and sex of the child were retrieved from birth medical records. Child BMI was calculated using the height and weight measured at study visit by trained personal.

*Cell type correction*

Cell-type adjustment was applied using the estimateCellCounts2 function in the FlowSorted.Blood.EPIC (v1.2.0) minfi package in R (v3.6.0) with the reference platform IlluminaHumanMethylationEPIC and included six available cell types: CD4+ T-lymphocytes, CD8+ T-lymphocytes, natural killer cells, B-lymphocytes, monocytes and neutrophils, in the regression models.

*Batch correction*

To account for potential technical effects in the data we used the functional normalization approach described by Fortin et al.,^59^ using the first 10 PCs of the Illumina EPIC array control probes. Potential batch effects (scan date, plate, beadchip, sentrix position) were checked after normalization using hierarchical clustering supported by significance testing and PCA considering the first six PCs for each batch effect variable. As no major batch effect was observed, we did not include technical variables in the regression models.

*Exclusion criteria*

For the present analysis we excluded n=66 mother-child pairs: 4 Non-European, 1 twin offspring, 10 children born preterm, 48 without DNA methylation data and 3 with missing covariates.

## Pre-, Peri-, and Postnatal Stress: Epigenetic impact on Depression (POSEIDON)

*Design and study population*

POSEIDON is a longitudinal birth cohort, that has recruited mothers (n=410) and their newborn children (n=405) in the Rhine-Neckar Region in Germany from 2010 to 2017. The recruitment waves included the third trimester of pregnancy (T1), at childbirth (T2), six months after birth (T3), and 45 months after birth (T4). In 2020, a reassessment with online questionnaires was conducted (T5).

In order to be included in the study mothers had to be at least 16 and at most 45 years of age, german speaking, and the main caregiver of the child. Newborn children born at 30 weeks of pregnancy or earlier, weighing under 1500 grams, and multiple births were excluded from the study. The cohort is described in detail elsewhere.^60,61^

*Ethics statement*

All participants of the POSEIDON study provided written informed consent. The ethical approval was received by the Ethics Committee of the Medical Faculty of Mannheim, Heidelberg University. The study was conducted in accordance with the Declaration of Helsinki.

*Maternal education*

Maternal education was self-reported during the third pregnancy trimester, mothers were asked for their highest educational level, highest training qualification, and highest degree (college, academy, or university). These levels were transformed to the ISCED 1997 educational levels equivalent to US years of schooling.

*Methylation measurements*

From n=313 newborns cord blood was collected immediately after birth, DNA extraction was performed using the chemagic Magnetic Separation Module I (Chemagen Biopolymer-Technologie AG; Baesweiler; Germany). Quantification of DNA methylation was achieved using Illumina HumanMethylation450 Beadchip.

*Covariates*

Maternal smoking was assessed during substance anamnesis at T1 and recoded in a binary variable (yes/no). Gestational age was included as age in weeks at childbirth. The sex of the child is binary (female/ male). Maternal age was self-reported in years at T1. Maternal BMI was calculated using the information on height and weight reported by the mother at T1.

*Cell type correction*

Cell type compositions for cord blood were estimated as recommended in Salas LA et al. (2019) and afterward included as covariates in the regression models.

*Batch correction*

Accounting for batch effects was done by conducting a principal component analysis (PCA) for the array control probes, the first 10 principal components (PCAs) were included in the regression models.

*Exclusion criteria*

For the final analysis samples with no phenotypic data or no methylation data were excluded. Furthermore, genetic outliers and participants having missings on one of the covariates were excluded resulting in a final sample of n=295 for model 1 and n=294 for model 2 and model 3.

## The Prediction and Prevention of Preeclampsia and Intrauterine Growth Restriction (PREDO)

The Prediction and Prevention of Preeclampsia and Intrauterine Growth Restriction (PREDO) study is a prospective, multicenter study of Finnish women who were pregnant between 2005 and 2009 and their children. PREDO recruited 1079 women with a singleton, intrauterine pregnancy, who visited antenatal clinics at any of the 10 study hospitals for their first routine ultrasound screening at 12 to 13 weeks of gestation, of whom 969 had one or more and 110 had none of the known risk factors for preeclampsia and intrauterine growth restriction.^62^

All participating mothers provided written informed consent. The study protocol was approved by the Ethics Committee of Obstetrics and Gynaecology and Women, Children and Psychiatry of the Helsinki and Uusimaa Hospital District and by the participating hospitals. The study has been registered as ClinicalTrials.gov identifier ISRCTN14030412.

*Maternal Education*

Maternal socioeconomic class based on self-reported level of education during pregnancy, classified into primary, secondary, lower tertiary, or upper tertiary which were converted the values 10, 13, 15 and 19, respectively, in order to correspond to years of schooling in US.

*Methylation measurements*

We used the *Illumina Infinium® HumanMethylation450 BeadChip* to measure DNA methylation in cord blood. To limit batch effects, we randomized all samples over the 96-well plates, based on gender and maternal risk factors for pre-eclampsia. Samples were placed on 96-well plates. Bisulfite conversion was performed using the EZ-96 DNA methylation kit (Zymo research Corporation, Irvine, USA). Then we used the *Infinium HumanMethylation450 BeadChip* (Illumina Inc., San Diego, USA) to measure the methylation level as a beta value ranging from 0 (no methylation) to 1 (complete methylation). The quality control pipeline was set up using the R-package *minfi*.^7^ Three IDs were excluded as they were outliers in the median intensities. Furthermore, 20 IDs showed disconcordance between phenotypic sex and estimated sex and were excluded. Nine IDs were contaminated with maternal DNA and were also removed.^63^ Methylation beta-values were normalized using the *funnorm* function. We excluded any probes on chromosome X or Y, probes containing SNPs and cross-hybridizing probes according to Chen et al.^17^ and Price et al.^64^ Furthermore, any CpGs with a detection p-value > 0.01 in at least 25% of the samples were excluded. After normalization two batches, i.e. slide and well, were significantly associated and were removed iteratively using the *Combat* method. Final number of CpGs in the data was 399953.

*Covariates*

Child sex: Derived based on identitycode.

Data on gestational age at birth, and maternal smoking during pregnancy (yes/no), pre-pregnancy BMI, and age were derived from the Finnish Medical Birth Register. 4.0% mothers reported smoking during pregnancy.

Ancestry/ethnicity: Four first principal components from genotyped data were used as ancestry covariates.

*Cell type correction*

The Salas cord blood reference set ([Gervin et al. 2019](https://pubmed.ncbi.nlm.nih.gov/31455416/)) was used to estimate the cell types in the cord blood.

*Batch correction*

The *ComBat* method in the *sva* package in R was used for the batch correction.

*Exclusion criteria*

We excluded participants with missing information on methylation, maternal education and covariates. The final sample (N=780) did not include any twins or sibling pairs.

## The Raine Study

*Design and study population*

Between 1989 to 1991, the Raine Study recruited 2,900 pregnant women (Gen 1) at 16 weeks gestation to participate in a study at the King Edward Hospital in Perth, Australia. 2,868 live-born individuals (Gen 2) entered the study. At the age of 17 the Gen 2 participants (n=1,192) took part in a follow-up including a clinical examination and provided a blood sample. Using whole-blood samples collected at age 17 years, epigenome-wide DNA methylation profiles for 1,192 (58 technical replicates). Quality control was performed using the statistical software R and Bioconductor packages shinyMethyl (Fortin et al., 2014), MethylAid (Van Iterson et al., 2014), and RnBeads (Assenov et al., 2014).

Four participants with inconsistent results and identified as outliers (n = 3) or sex misclassification (n = 1) were removed. Sixty-five CpGs for which a common SNP disrupted the site leading to genotypic specific methylation levels, 11,648 sex chromosome CpGs and 10,777 CpGs with a detection p > 0.05 in any sample were removed. A further 160 probes with bead counts <3 in more than 5% of samples were removed. Batch effects persisted after beta-mixture quantile normalization (BMIQ) was applied (Teschendorff et al., 2013). Therefore, plate, slide, and well number were included in all statistical models. Mapping of the CpG to the nearest gene was performed using the Illumina Infinium annotation genomic coordinates.

*Ethics statement*

Ethics approval was obtained from the Human Ethics Committees at King Edward Memorial Hospital, Princess Margaret Hospital, The University of Western Australia and Curtin University.

*Maternal education*

Information on maternal education was self-reported by mother during pregnancy using the 16 week gestation questionnaire. Two variables were used to harmonise this variable. The first question asked was ‘Highest level of education’ and response categories included following: 0) none, 1) Trade certificate or apprenticeship, 2) Professional registration (non-degree), 3) College diploma or degree, 4) University degree, and 5) Other. The second question asked was ‘Highest school year completed’ and the response ranged from 2 to 12. These categorisations and school year range were used to harmonise a variable that was in accordance with the ISCED 1997 classification equivalent to US years of schooling in the study. Our data did not allow differentiate between levels 5 and 6 of ISCED classification and thus as per the analytical plan guidance, everyone with tertiary level education was imputed at level 5 and indicated as 20 years of schooling.

*Methylation measurements*

Methylation of genomic DNA was quantified using the Illumina HumanMethylation450 and generated at the Centre for Molecular Medicine and Therapeutics, University of British Columbia using the Illumina Infinium HumanMethylation450 BeadChip array (Illumina San Diego, CA).

*Covariates*

Maternal data was collected during antenatal visits using in-person interviews and clinical examinations. Data on maternal smoking status during pregnancy at 16 and 34 weeks was self-reported and dichotomised as ‘any smoking during pregnancy’ and ‘no smoking’. Maternal age was assessed at first antenatal visit. Gestational age was established by last menstrual period of the mother and cross-evaluated through ultrasound examination. Maternal pre-pregnancy BMI was calculated using information on height recorded at the first 16 week gestation visit and weight was self-reported in the questionnaire. Height and weight were measured converted into pre-pregnancy BMI. Offspring data was collected during follow-up at 17 years of age and their smoking status, age and gender were self-reported in questionnaire. Height and weight were measured during clinical examination and converted to BMI as kg/m^2^.

*Cell type correction*

As cellular heterogeneity can influence methylation profiles and drive some of the methylation differences detectable across individual blood samples, we adjusted for estimated cell counts using the Houseman^3^ estimating method as implemented in the R statistical package, minfi ([Aryee et al., 2014](https://www.frontiersin.org/articles/10.3389/fgene.2019.00770/full#B2)) for six cell types (CD8T, CD4T, NK, B cell, Monocytes, and Granulocytes).

*Batch correction*

To account for batch effects in the data, plate, well number, and slide position were included as technical covariates in all statistical models. All models were corrected for batch by including the **Plate_no** variable, indicating the batch. In total, the Raine Study epigenetic data was measured on 19 batches.

*Exclusion criteria*

Raine Study: For the main analysis we excluded n=197 participants: 197 individuals of non-European descent.

## The Swedish Twin study On Prediction and Prevention of Asthma (STOPPA)

*Design and study population*

The STOPPA is a twin cohort study including 752 individuals.^65^ Twins 9-14 years of age were selected from an on-going data collection within the Child and Adolescent Twin study in Sweden (CATSS) based on the pair’s asthma status.^66^ Asthma concordant (ACC), asthma discordant (ADC) and healthy concordant (HCC) pairs were included and invited to take part in test centre visits including clinical examination, questionnaires, lung function testing and collection of biosamples. Further details regarding STOPPA have been provided in a separate publication.^65^ The study population has been then linked to the Swedish population-based Medical Birth Register (MBR) to retrieve information on several covariates.^67^ Whole blood samples were available on 708 twins.

*Ethics statement*

The study was approved by the regional ethical review board in Stockholm, Sweden. Written informed consent was collected from the study participants and their parents.

*Maternal Education*

We used maternal education information based on the parent’s answer from the STOPPA questionnaire. The question asked was ”What is the mother’s highest education?” and response categories included following: 1) less than 9 years of compulsory education, 2) 9 years of compulsory education, 3) High school education less than two years, 4) Completed high school education, 5) University education less than three years, 6) University education three years or longer, 7) Research education. Maternal education were then categorized according to the ISCED 1997 classification.

*Methylation measurements*

DNA was extracted from whole blood (collected in a 4ml EDTA tube) using the Chemagic Star 400 kit (PerkinElmer chemagen, Baesweiler, Aachen, Germany) according to a standardized protocol. Samples were allocated between analysis plates and chips by complete randomization, with the exception that samples from twin pairs were kept within the same chip to allow for within-pair comparisons free of batch effects. Analyses were performed at the Mutation Analysis Facility (MAF) at Karolinska Institutet using the Infinium HumanMethylation450 Beadchip Kit (Illumina, Inc., San Diego, California, USA). Probe filtering and normalization was carried out using RnBeads package in R and the dasen method.^18^ Probes overlapping with single nucleotide polymorphisms or specific nucleotide contexts (10,131 probes), due to unreliable measurements (defined as detection p-values > 5*10^-8^, removed 2982 probes with low quality) were filtered out, leaving 468,209 CpG probes. After additional filtering using the 3IQR method, a final number of 451,728 probes remained for analysis.

*Covariates*

Covariates considered in the analysis were: ancestry information measured as mother’s birth country (Sweden/Other Nordic countries/Rest of the world), maternal age at first antenatal visit (in years), mid-wife reported height and weight at first antenatal visit and then converted to BMI as kg/m^2^ (continuous), maternal smoking during pregnancy (yes, no), gestational age (in weeks), all retrieved from the MBR. Furthermore, we also included child BMI as kg/m^2^ (continuous), sex (male, female), age at the clinical examination (in years), and selection factor for the STOPPA cohort (control, asthma/wheezing) retrieved from the STOPPA questionnaire and the clinical examination data.

Child smoking data was not available in STOPPA.

*Cell type correction*

We used the minfi package in R to correct cell type heterogeneity followed the Houseman algorithm.

*Batch correction*

We added the batch variables in all statistical models to adjust for the batch effects.

*Exclusion criteria*

To simplify the methylation analysis, we excluded participants whose mothers were not born in Sweden or with missing data on mother’s birth country (n=74).

After exclusions of children with known congenital anomalies at birth (n=9), and children with missing data on covariates or outcomes (n=213), 412 twins were with complete phenotype and covariate data. In order to avoid relatedness of sample in the analyses, we kept one twin-per-pair and 219 twins remained in the analysis.

## Project Viva

Project Viva is an ongoing pre-birth cohort recruited from a multi-specialty group practice in eastern Massachusetts (Atrius Harvard Vanguard Medical Associates). Details on study design and recruitment are reported elsewhere.^68^ In brief, we recruited pregnant women at their initial prenatal visit between 1999 and 2002. Exclusion criteria included multiple gestation, inability to answer questions in English, gestational age ≥22 weeks and plans to move away before delivery. We completed in-person visits with mothers during pregnancy in the first (median 9.9 weeks of gestation) and second (median 27.9 weeks) trimesters. We saw mothers and children in the hospital during the delivery admission, and for in-person research visits during infancy (median age 6.3 months), early childhood (median 3.2 years), mid-childhood (median 7.7 years), and in early adolescence (median 13.1 years). At these visits, we implemented questionnaires, collected blood, and measured anthropometry, body composition, and blood pressure. For this study, we used DNA methylation data from blood cells collected at birth (cord blood) and at the mid-childhood visit (median 7.7 years).

The Institutional Review Board of Harvard Pilgrim Health Care approved all study protocols. All mothers provided written informed consent and children provided verbal assent.

*Inclusion / Exclusion criteria for the current analyses*

Project Viva is a mixed ancestry cohort. As requested in the analysis plan, we included only white participants with complete observations for the exposure of interest (maternal education – collected at the early pregnancy visit) and all covariates. We included N=344 for the cord blood DNA methylation analysis and N=289 for the mid-childhood blood DNA methylation analysis.

*Maternal Education*

During the early pregnancy interview, we asked the woman to indicate her educational level as “less than 12^th^ grade,” “High school degree or a GED,” “Some college or an associate’s degree,” “4 years of college (BA/BS),” or Graduate degree (Master’s, Ph.D.).” We coded the level of education as instructed by the common analytic plan for all PACE cohorts contributing to this meta-analysis: “less than 12^th^ grade” = 10 years of schooling, “High school degree or a GED” = 13 years of schooling, “Some college or an associate’s degree” = 15 years of schooling, “4 years of college (BA/BS)” = 19 years of schooling, or “Graduate degree (Master’s, PhD)” = 20 years of schooling.

*Methylation measurements*

Methods for genome-wide DNA methylation analysis have been published.^69^ In brief, we bisulfite-converted buffy coat DNA (EZ DNA Methylation-Gold Kit, Zymo Research, Irvine, CA) in cord blood, and in peripheral leukocytes from mid-childhood.^70^ We shipped samples to Illumina Inc., where they were analyzed using the Infinium HumanMethylation450 BeadChip (Illumina, San Diego, CA). This array is widely-used in epidemiologic studies and has been previously validated. Upon receiving the data, we removed technical replicates, samples with low quality, genotype mismatch, and sex mismatch. Next, we removed non-CpG probes, and probes on the X and Y chromosome. We also flagged and removed CpG probes with low detection P-values >0.05. We used the normal-exponential out-of-band (NOOB) method for background correction and dye bias adjustment and normalized our sample with Beta Mixture Quantile Dilation (BMIQ). To control for technical variability across sample plates we applied the R package ComBat.

*Covariates*

Maternal age: We derived maternal age at enrolment (years) from the mother’s date of birth and date of enrolment in early pregnancy.

Maternal BMI: We calculated maternal BMI (kg/m^2^) from self-reported height and pre-pregnancy weight, which were collected by questionnaire during the first trimester of pregnancy.

Maternal smoking was self-reported by questionnaires during pregnancy (0 = never smoked during pregnancy or 1 = ever smoked during pregnancy). We did not use sustained smoking during pregnancy due to extremely small sample size.

Newborn sex was obtained from delivery interviews and hospital records (0 = male; 1 = female).

Gestational age at delivery: calculated from the date of the last menstrual period, or from a 2^nd^ trimester ultrasound if the estimated delivery date differed for >10 days. Gestational age in weeks was used in the analyses.

*Cell type correction*

Cell type composition at each research visit was estimated from genome-wide DNA methylation arrays (HumanMethylation450 BeadChip; Illumina) using the *minfi* package in R (version 3.3.0, R Core Team).^7^ We used an adult reference DNA methylation panel to estimate leukocyte composition in mid-childhood and a cord blood DNA methylation reference panel, which includes nucleated red blood cells, to estimate nucleated cell types at birth.

*Batch correction*

To control for technical variability across sample plates, we applied the R sva package ComBat. Combat was used to adjust for sample plate while protecting the maternal education variable, as well as some of the covariates.

# **Cohort-specific acknowledgements**

**ALSPAC:** We are extremely grateful to all the families who took part in this study, the midwives for their help in recruiting them, and the whole ALSPAC team, which includes interviewers, computer and laboratory technicians, clerical workers, research scientists, volunteers, managers, receptionists, and nurses. We would like to acknowledge Tom Gaunt, Oliver Lyttleton, Sue Ring, Nabila Kazmi, and Geoff Woodward for their earlier contribution to the generation of ARIES data (ALSPAC methylation data).

**CHS:** We would like to express our sincere gratitude to Martin Kharrazi, Steve Graham, and Robin Cooley at the California Biobank Program and Genetic Disease Screening Program within the California Department of Public Health for their assistance and advice regarding newborn bloodspots. We are indebted to the school principals, teachers, students and parents in each of the study communities for their cooperation and especially to the members of the health testing field team for their efforts.

**EARLI:** We thank the participants of the EARLI study.

**MARBLES:** We are grateful to the participants of the MARBLES Study, without whom this work would not have been possible.

**EDEN:** We are extremely grateful to all the families who took part in this study, the midwives and psychologists for recruiting and following them, and the whole EDEN team, including research scientists, engineers, technicians and managers

**ENVIRONAGE:** The authors are extremely grateful to the participating women and neonates, as well as the staff of the maternity ward, midwives, and the staff of the clinical laboratory of East-Limburg Hospital in Genk.

**Exposomic ENVIRONAGE:** We are extremely grateful to the participating women and neonates, as well as the staff of the maternity ward, midwives, and the staff of the clinical laboratory of East-Limburg Hospital in Genk. We thank Dr. Paolo Vineis for coordinating the EXPOsOMICS project and Mr. Cyrille Cuenin and Mr. Vincent Cahais for their help in this project.

**Exposomic Piccolipiù:** Our thanks go to all the families who took part in this study, to the midwives for their help in recruiting them, and to the whole Piccolipiù team, which includes doctors, nurses, research scientists and computer/laboratory technicians. We thank Dr. Paolo Vineis for coordinating the EXPOsOMICS project and Mr. Cyrille Cuenin and Mr. Vincent Cahais for their help in this project.

**Exposomic Rhea:** We are extremely grateful to all the families who took part in the Rhea study, the midwives, research assistants and psychologists for recruiting and following them, and the whole Rhea team, including research scientists, biologists and technicians for their commitment and their role in the success of the study. We thank Mariona Bustamante (CREAL, Barcelona, Spain) for maintaining the Rhea DNA biobank. We thank Dr. Paolo Vineis for coordinating the EXPOsOMICS project and Mr. Cyrille Cuenin and Mr. Vincent Cahais for their help in this project.

**FinnGeDi:** We thank all families for participating and research doctors, midwifes and other personnel in the study hospitals and in National Institute for Health and Welfare for their contribution to FinnGeDi study.

**GECKO:** The authors are grateful to the families who took part in the GECKO Drenthe study, the midwives, gyneacologists, nurses, and the general practitioners and all health professionals at the Preventive Child Healthcare Drenthe for their help in the recruitment and the measurements, and the GECKO Drenthe study team.

**Generation R:** The Generation R Study is conducted by Erasmus MC, University Medical Center Rotterdam in close collaboration with the School of Law and Faculty of Social Sciences of the Erasmus University Rotterdam, the Municipal Health Service Rotterdam area, Rotterdam, the Rotterdam Homecare Foundation, Rotterdam and the Stichting Trombosedienst & Artsenlaboratorium Rijnmond (STAR-MDC), Rotterdam. We gratefully acknowledge the contribution of children and parents, general practitioners, hospitals, midwives and pharmacies in Rotterdam. The study protocol was approved by the Medical Ethical Committee of Erasmus MC. Written informed consent was obtained for all participants. The generation and management of the Illumina 450K methylation array data (EWAS data) for the Generation R Study was executed by the Human Genotyping Facility of the Genetic Laboratory of the Department of Internal Medicine, Erasmus MC, and the Netherlands. We thank Mr. Michael Verbiest, Ms. Mila Jhamai, Ms. Sarah Higgins, Mr. Marijn Verkerk and Dr. Lisette Stolk for their help in creating the EWAS database. We thank Dr. A.Teumer for his work on the quality control and normalization scripts.

**HAVEN:** We thank the parents of the children and the Erasmus MC University Medical center array facility for DNA isolation and measurements.

**INMA:** The authors would particularly like to thank all the participants for their generous collaboration. A full roster of the INMA Project Investigators can be found at <http://www.proyectoinma.org/presentacion-inma/listado-investigadores/en_listado-investigadores.html>.

**LINA:** We thank Melanie Bänsch, Anne Hain, Beate Fink, and Michaela Loschinski for their excellent technical assistance and field work. Furthermore we cordially thank the LINA children and their families for their ongoing participation, and our clinical cooperation partners.

**MOBA:** We are grateful to all the participating families in Norway who take part in this on-going cohort study. We thank Dr. Bonnie Joubert of NIEHS for her extensive work on the quality control and initial analysis of the MoBa1 and MoBa2 datasets and Elin Alsaker of the National Institute of Public Health (Bergen, Norway), Dr. Frank Day of NIEHS and Dr. Jianping Jin of Westat (Durham, NC) for expert data management and computational assistance.

**NEST:** We thank the parents and other caregivers of the Newborn Epigenetics STudy. We also thank the field and laboratory staff for their effort.

**NFBC1986:** We thank all cohort members and researchers who have participated in the study. We also acknowledge the work of the NFBC project center.

**POGO:** We thank Melanie Bunk, Susanne Hivner and Daniela Much (Institute for Diabetes Research, Helmholtz Zentrum München, Germany) for data and sample collection at the study site visits, and the families for participating in the study.

**PREDO:** The PREDO study would not have been possible without the dedicated contribution of the PREDO study group members: E Hamäläinen, E Kajantie, H Laivuori, PM Villa, A-K Pesonen, A Aitokallio-Tallberg, A-M Henry, VK Hiilesmaa, T Karipohja, R Meri, S Sainio, T Saisto, S Suomalainen-Konig, V-M Ulander, T Vaitilo (Department of Obstetrics and Gynaecology, University of Helsinki and Helsinki University Central Hospital, Helsinki, Finland), L Keski-Nisula, Maija-Riitta Orden (Kuopio University Hospital, Kuopio Finland), E Koistinen, T Walle, R Solja (Northern Karelia Central Hospital, Joensuu, Finland), M Kurkinen (Päijät-Häme Central Hospital, Lahti, Finland), P.Taipale. P Staven (Iisalmi Hospital, Iisalmi, Finland), J Uotila (Tampere University Hospital, Tampere, Finland). We thank all the PREDO children and their parents for their enthusiastic participation. We also thank all the research nurses, research assistants, and laboratory personnel involved in the Predo study.

**RAINE:** We would like to acknowledge the Raine Study participants and their families for their ongoing participation in the study and the Raine Study team for study co-ordination and data collection. We also thank the NHMRC for their long term contribution to funding the study over the last 30 years. The core management of the Raine Study is funded by The University of Western Australia, Curtin University, Telethon Kids Institute, Women and Infants Research Foundation, Edith Cowan University, Murdoch University, The University of Notre Dame Australia and the Raine Medical Research Foundation.

**STOPPA:** First, we direct our greatest appreciation to the twins and parents of the STOPPA cohort, without whose participation this study could not have been performed. We are also indebted to the STOPPA research nurses and database managers for their excellent data collection and data managing. We also want to direct our thanks to the eight paediatric allergy clinics around Sweden for their great collaboration during our visits for clinical examinations. We acknowledge the Swedish Twin Registry for access to data. The Swedish Twin Registry is managed by Karolinska Institutet and receives funding through the Swedish Research Council under the grant no 2017-00641. We also acknowledge the Biobank at Karolinska Institutet for professional biobank service.

**Project Viva:** We thank the participants and staff of Project Viva.

# **Cohort-specific funding statements**

**ALSPAC:** GM and GCS are members of the MRC Integrative Epidemiology Unit, which receives funds from the University of Bristol and United Kingdom Medical Research Council [MC_UU_00011/1, MC_UU_00011/5]. GCS’s contribution to this work is supported by the Medical Research Council [New Investigator Research Grant, MR/S009310/1]. GM and GCS’s contributions are supported by the European Joint Programming Initiative “A Healthy Diet for a Healthy Life” (JPI HDHL, NutriPROGRAM project, UK MRC MR/S036520/1]. The UK Medical Research Council and the Wellcome Trust (Grant ref: 217065/Z/19/Z) and the University of Bristol provide core support for ALSPAC. A comprehensive list of grants funding is available on the ALSPAC website (http://www.bristol.ac.uk/alspac/external/documents/grant-acknowledgements.pdf). The Accessible Resource for Integrated Epigenomics Studies (ARIES) which generated large scale methylation data was funded by the UK Biotechnology and Biological Sciences Research Council (BB/I025751/1 and BB/I025263/1). Additional epigenetic profiling on the ALSPAC cohort was supported by the UK Medical Research Council Integrative Epidemiology Unit and the University of Bristol (MC_UU_12013_1, MC_UU_12013_2, MC_UU_12013_5 and MC_UU_12013_8), the United States National Institute of Health (5RO1AI121226-02) and National Institute of Child and Human Development grant (R01HD068437). The funders had no role in study design, data collection and analysis, decision to publish, or preparation of the manuscript. This publication is the work of the authors and Gemma Sharp will serve as guarantors for the contents of this paper. The views expressed in this paper are those of the authors and not necessarily any funders. The funders had no influence on the content of the paper.

**CHS:** The CHS was supported by the following NIH grants: K01ES017801, R01ES022216, P30ES007048, R01ES014447, P01ES009581, R826708-01 and RD831861-01.

**EAGeR:** The EAGeR trial was supported by the Intramural Research Program of the *Eunice Kennedy Shriver* National Institute of Child Health and Human Development (National Institutes of Health, Bethesda, MD, USA) under contract numbers HHSN267200603423, HHSN267200603424, HHSN267200603426, and HHSN275201300023I-HHSN2750008.

**EARLI:** Funding for the EARLI study was provided by the National Institutes of Health (R01 ES016443, PI: Newschaffer; R24ES030893, PI: Fallin; R01ES025531, PI: Fallin) and Autism Speaks (003953 PI: Newschaffer). Mr. Dou and Dr. Bakulski were supported by grants from the National Institutes of Health (R01 ES025531, PI: Fallin; R01 MD013299). The content is solely the responsibility of the authors and does not necessarily represent the official views of the National Institutes of Health.

**MARBLES:** The MARBLES study and this work has been supported by a grant from the Allen Foundation, pilot funding from the MIND Institute, EPA STAR grant #RD-83329201, and NIH grants: R01ES025574, R01ES029213, R24ES028533, R01ES028089, R01ES020392, P01ES011269, and K12HD051958. These supporting organizations had no role in the design and conduct of the work; collection, management, analysis, and interpretation of the data; preparation, review, or approval of the manuscript; and decision to submit the manuscript for publication. The findings and conclusions in this report are those of the authors and do not necessarily represent the official position of the National Institutes of Health or EPA.

**EDEN:** The EDEN cohort has been funded by Foundation for Medical Research (FRM), National Agency for Research (ANR), National Institute for Research in Public Health (IRESP: TGIR cohorte santé 2008 program), French Ministry of Health (DGS), French Ministry of Research, Inserm Bone and Joint Diseases National Research (PRO-A) and Human Nutrition National Research Programs, Paris–Sud University, Nestlé, French National Institute for Population Health Surveillance (InVS), French National Institute for Health Education (INPES), the European Union FP7 programmes (FP7/2007-2013, HELIX, ESCAPE, ENRIECO projects, FP7-ENV HEALS), Diabetes National Research Program (through a collaboration with the French Association of Diabetic Patients (AFD)), French Agency for Environmental Health Safety (now ANSES), Mutuelle Générale de l’Education Nationale (MGEN), French National Agency for Food Security, and the French-speaking association for the study of diabetes and metabolism (ALFEDIAM). The cord and whole blood methylome has been assessed in the frame of the MeDALL project, which was supported by the European Union under the Health Cooperation Work Programme of the 7th Framework programme (grant no. 261357).

**ENVIRONAGE:** The ENVIRONAGE birth cohort is supported by the European Research Council [ERC-2012-StG.310898], and by funds of the Flemish Scientific Research council [FWO, G.0.733.15.N].

**Exposomic ENVIRONAGE:** The ENVIR*ON*AGE birth cohort is supported by the European Research Council [ERC-2012-StG.310898], and by funds of the Flemish Scientific Research council [FWO, G.0.733.15.N]. The DNA methylation assays were funded by the European Community's Seventh Framework Programme FP7/2007–2013 project EXPOsOMICS (grant no. 308610).

**Exposomic Piccolipiù:** Piccolipiù cohort was approved and initially funded by the Italian National Centre for Disease Prevention and Control (CCM grant 2010) and by the Italian Ministry of Health (art 12 and 12bis Dl.gs.vo 502/92). The DNA methylation assays were funded by the European Community's Seventh Framework Programme FP7/2007–2013 project EXPOsOMICS (grant no. 308610).

**Exposomic Rhea:** Rhea acknowledge all funding sources for the Rhea study: the European Union H2020 (LIFECYCLE), FP7 (HELIX, CHICOS, EnviroGenomarkers, ENRIECO, ESCAPE) and FP6 (HiWate, NewGeneris) programmes. The National Strategic Reference Framework (ESPA) 2007–13, the General Secretariat for Research and Technology in Greece and the Research Committee of the University of Crete, Greece. Funders had no influence of any kind on analyses or results interpretation. The DNA methylation assays were funded by the European Community's Seventh Framework Programme FP7/2007–2013 project EXPOsOMICS (grant no. 308610).

**FinnGeDi:** Academy of Finland; Diabetes Research Foundation; Foundation for Pediatric Research; Juho Vainio Foundation; Novo Nordisk Foundation; Signe and Ane Gyllenberg Foundation; Sigrid Jusélius Foundation; Yrjö Jahnsson Foundation; Finnish Medical Foundation; Research Funds of Oulu University Hospital and Helsinki University Hospital (state grants), Medical Research Center Oulu and Finnish Institute for Health and Welfare.

**GECKO:** The GECKO Drenthe birth cohort was funded by an unrestricted grant of Hutchison Whampoa Ltd, Hong Kong and supported by the University of Groningen, Well Baby Clinic Foundation Icare, Noordlease, Paediatric Association Of The Netherlands, Youth Preventive Health Care Drenthe and European Union’s Horizon 2020 research and innovation programme (LifeCycle, Grant Agreement No. 733206 LifeCycle).

**Generation R:** The general design of the Generation R Study is made possible by financial support from Erasmus MC, University Medical Centre Rotterdam, Erasmus University Rotterdam, the Netherlands Organization for Health Research and Development (ZonMw), the Netherlands Organization for Scientific Research (NWO), the Ministry of Health, Welfare and Sport and the Ministry of Youth and Families. The EWAS data was funded by a grant to VWVJ from the Netherlands Genomics Initiative (NGI)/Netherlands Organization for Scientific Research (NWO) Netherlands Consortium for Healthy Aging (NCHA; project number 050-060-810), by funds from the Genetic Laboratory of the Department of Internal Medicine, Erasmus MC, and by a grant from the National Institute of Child and Human Development (R01HD068437). The project was supported by funding from the European Union's Horizon 2020 research and innovation program under grant agreements No 733206 (LifeCycle), 874739 (LongITools) and 824989 (EUCAN-Connect), 848158 (EarlyCause) and from the European Joint Programming Initiative “A Healthy Diet for a Healthy Life” (JPI HDHL, NutriPROGRAM project, ZonMw the Netherlands no.529051022 and PREcisE project ZonMw the Netherlands no.529051023).

**HAVEN:** E.W. Tobi was supported by a VENI grant from the Netherlands Organization for Scientific Research (91617128). This work was funded by the Joint Programming Initiative – A Healthy Diet for a Healthy Life (JPI HDHL) (proposal number 655). In the UK it is jointly funded by the Medical Research Council (MRC) and the Biotechnology and Biological Sciences Research Council (BBSRC) [grant reference: MR/S03658X/1]; in Spain by Instituto de Salud Carlos III [PCI2018-093147], in Germany by the German Federal Ministry of Education and Research [FKZ 01EA1905]; ZonMw in the Netherlands [529051023]; and in France by French National Research Agency [ANR18-HDHL-0003-05].

**Healthy Start study:** The Healthy Start study was supported by grants from the National Institute of Environmental Health Sciences (R01ES022934), the National Institute of Diabetes and Digestive and Kidney Diseases (R01DK076648), and the National Institutes of Health Office of the Director (UH3OD023248).

**INMA:** This study was funded by grants from Instituto de Salud Carlos III (Red INMA G03/176; CB06/02/0041; PI041436), PI081151 incl. FEDER funds), Generalitat de Catalunya-CIRIT 1999SGR 00241, Fundació La marató de TV3 (090430), and 261357 (MEDALL project), the European Community’s Seventh Framework Programme - European Research Council (ERC) grant agreement no 268479 (BREATHE Project), the European Union’s Horizon 2020 research and innovation programme under grant agreement no 733206 (LIFECYCLE). Jose Urquiza is supported by the program PERIS (Ref.: SLT017/20/000119), granted by Departament de Salut de la Generalitat de Catalunya (Spain).

**LINA:** The core funding for the LINA study is provided by the Helmholtz Center for Environmental Research –UFZ / Department of Environmental Immunology. The methylation analysis component of the LINA study was supported by the German Cancer Research Centre – DKFZ.

**MOBA:** The Norwegian Mother and Child Cohort Study are supported by the Norwegian Ministry of Health and Care Services and the Ministry of Education and Research, NIH/NIEHS (contract no N01-ES-75558), NIH/NINDS (grant no.1 UO1 NS 047537-01 and grant no.2 UO1 NS 047537-06A1). For this work, MoBa 1 and 2 were supported by the Intramural Research Program of the NIH, National Institute of Environmental Health Sciences (Z01-ES-49019) and the Norwegian Research Council/BIOBANK (grant no 221097). This work was partly supported by the Research Council of Norway through its Centres of Excellence funding scheme, project number 262700. MoBa3 DNA methylation assays were funded by the grant from Institut National du Cancer (INCa) / INSERM-Plan Cancer (France, 2015), the International Childhood Cancer Cohort Consortium (I4C), and the IARC Postdoctoral Fellowship, partially supported by the EC FP7 Marie Curie Actions-People-Co-funding of regional, national and international programmes (COFUND).

**NEST:** The NEST study was funded by NIEHS grants R21ES014947 and R01ES016772 and NIDDK grant R01DK085173. CH and DDJ have received funding from the National Institute of Environmental Health Science (P30 ES025128). CH, SKM, and RLM have received funding from the National Institute of Environmental Health Science (R24ES028531).

**NFBC1986:** NFBC1986 received financial support from EU QLG1-CT-2000-01643 (EUROBLCS) Grant no. E51560, NorFA Grant no. 731, 20056, 30167, USA / NIH 2000 G DF682 Grant no. 50945. The researchers received funding from European Union's Horizon 2020 research and innovation program under grant agreement No. 633595 (DynaHEALTH), grant agreement no. 733206 (LifeCycle), grant agreement no. 873749 (LongITools), grant agreement no. 848158 (EarlyCause), and the Academy of Finland Project 312123 and Profi6 AF 336449.

**POGO:** The POGO study is funded by the Institute of Diabetes Research, Helmholtz Zentrum München, Neuherberg, Germany, and the German Center for Diabetes Research (DZD e.V.). This study was supported by grants from the H2020 Joint Programming Initiative a Healthy Diet for a Healthy Life (JPI HDHL) under proposal number 655 (PREcisE Project), the German Federal Ministry of Education and Research [FKZ 01EA1905] and the European Union’s Horizon 2020 Research and Innovation programme (no. 633595) for the DynaHEALTH action.

**POSEIDON:** The POSEIDON work was supported by the German Research Foundation [DFG; grant FOR2107; RI908/11-2 and WI3429/3-2], the German Federal Ministry of Education and Research (BMBF) through the Integrated Network IntegraMent, under the auspices of the e:Med Programme [01ZX1314G; 01ZX1614G] through grants 01EE1406C, 01EE1409C and through ERA-NET NEURON, “SynSchiz - Linking synaptic dysfunction to disease mechanisms in schizophrenia - a multilevel investigation“ [01EW1810], through ERA-NET NEURON “Impact of Early life MetaBolic and psychosocial strEss on susceptibility to mental Disorders; from converging epigenetic signatures to novel targets for therapeutic intervention” [01EW1904] and by a grant of the Dietmar-Hopp Foundation.

**PREDO:** The PREDO Study has been funded by the Academy of Finland (JL: 311617 and  269925, KR: 1312670 ja 128789 1287891), EraNet Neuron, EVO (a special state subsidy for health science research), University of Helsinki Research Funds, the Signe and Ane Gyllenberg foundation, the Emil Aaltonen Foundation, the Finnish Medical Foundation, the Jane and Aatos Erkko Foundation, the Novo Nordisk Foundation, the Päivikki and Sakari Sohlberg Foundation, Juho Vainio foundation, Yrjö Jahnsson foundation, The Finnish Society of Sciences and Letters, Jalmari and Rauha Ahokas foundation, Sigrid Juselius Foundation granted to members of the Predo study board. Methylation assays were funded by the Academy of Finland (269925).

**RAINE:** The Raine Study has been funded by program and project grants from the Australian National Health and Medical Research Council, the Commonwealth Scientific and Industrial Research Organisation, Healthway and the Lions Eye Institute in Western Australia. The Raine Study Gen2-17 year follow-up was funded by the NHMRC Program Grant (Stanley et al, ID 353514) and the GWAS data from the Gen2-17 year follow-up was funded by the NHMRC (Huang et al, ID 1059711) grant. The University of Western Australia (UWA), Curtin University, the Raine Medical Research Foundation, the Telethon Kids Institute, the Women’s and Infant’s Research Foundation (KEMH), Murdoch University, The University of Notre Dame Australia and Edith Cowan University provide funding for the Core Management of the Raine Study. REF is a recipient of a National Health and Medical Research Council Early Career Fellowship.RCH is supported by NHMRC ID1053384 and ID733206. AL is supported by an NHMRC Emerging Leader Fellowship ID 2010063. JC is supported by the National Health and Medical Research Council, Australia grant ID GNT114285.

**STOPPA:** Financial support was provided by the Swedish Research Council (grant no 2018-02640) and through the Swedish Initiative for research on Microdata in the Social And Medical Sciences (SIMSAM) framework grant number 340-2013-5867, grants provided by the Stockholm County Council (ALF projects), the Swedish Heart Lung Foundation, the Swedish Asthma and Allergy Association's Research Foundation, FORTE and Stiftelsen Frimurare Barnahuset Stockholm.

**Project Viva:** We received grants from the US National Institutes of Health (R01 HD034568, UH3 OD023286, R01 HL111108, R01 NR013945).

# **References**

1 Boyd A, Golding J, Macleod J, *et al.* Cohort Profile: the ’children of the 90s’--the index offspring of the Avon Longitudinal Study of Parents and Children. *Int J Epidemiol* 2013; **42**: 111–27.

2 Fraser A, Macdonald-Wallis C, Tilling K, *et al.* Cohort Profile: the Avon Longitudinal Study of Parents and Children: ALSPAC mothers cohort. *Int J Epidemiol* 2013; **42**: 97–110.

3 Relton CL, Gaunt T, McArdle W, *et al.* Data Resource Profile: Accessible Resource for Integrated Epigenomic Studies (ARIES). *Int J Epidemiol* 2015; **44**: 1181–90.

4 Min JL, Hemani G, Davey Smith G, Relton C, Suderman M. Meffil: efficient normalization and analysis of very large DNA methylation datasets. *Bioinformatics* 2018; **34**: 3983–9.

5 McConnell R, Berhane K, Yao L, *et al.* Traffic, Susceptibility, and Childhood Asthma. *Environ Health Perspect* 2006; **114**: 766–72.

6 Noushmehr H, Weisenberger DJ, Diefes K, *et al.* Identification of a CpG Island Methylator Phenotype that Defines a Distinct Subgroup of Glioma. *Cancer Cell* 2010; **17**: 510–22.

7 Aryee MJ, Jaffe AE, Corrada-Bravo H, *et al.* Minfi: a flexible and comprehensive Bioconductor package for the analysis of Infinium DNA methylation microarrays. *Bioinformatics* 2014; **30**: 1363–9.

8 Triche TJ, Weisenberger DJ, Van Den Berg D, Laird PW, Siegmund KD. Low-level processing of Illumina Infinium DNA Methylation BeadArrays. *Nucleic Acids Res* 2013; **41**: e90–e90.

9 Touleimat N, Tost J. Complete pipeline for Infinium ® Human Methylation 450K BeadChip data processing using subset quantile normalization for accurate DNA methylation estimation. *Epigenomics* 2012; **4**: 325–41.

10 Schisterman EF, Silver RM, Lesher LL, *et al.* Preconception low-dose aspirin and pregnancy outcomes: results from the EAGeR randomised trial. *Lancet* 2014; **384**: 29–36.

11 Yeung EH, Guan W, Mumford SL, *et al.* Measured maternal prepregnancy anthropometry and newborn DNA methylation. *Epigenomics* 2019; **11**: 187–98.

12 Gervin K, Salas LA, Bakulski KM, *et al.* Systematic evaluation and validation of reference and library selection methods for deconvolution of cord blood DNA methylation data. *Clin Epigenetics* 2019; **11**: 125.

13 Joyce EE, Chavarro JE, Rando J, *et al.* Prenatal exposure to pesticide residues in the diet in association with child autism‐related traits: Results from the <scp>EARLI</scp> study. *Autism Res* 2022; **15**: 957–70.

14 Hertz-Picciotto I, Schmidt RJ, Walker CK, *et al.* A Prospective Study of Environmental Exposures and Early Biomarkers in Autism Spectrum Disorder: Design, Protocols, and Preliminary Data from the MARBLES Study. *Environ Health Perspect* 2018; **126**: 117004.

15 Pidsley R, Zotenko E, Peters TJ, *et al.* Critical evaluation of the Illumina MethylationEPIC BeadChip microarray for whole-genome DNA methylation profiling. *Genome Biol* 2016; **17**: 208.

16 Heude B, Forhan A, Slama R, *et al.* Cohort Profile: The EDEN mother-child cohort on the prenatal and early postnatal determinants of child health and development. *Int J Epidemiol* 2016; **45**: 353–63.

17 Chen Y, Lemire M, Choufani S, *et al.* Discovery of cross-reactive probes and polymorphic CpGs in the Illumina Infinium HumanMethylation450 microarray. *Epigenetics* 2013; **8**: 203–9.

18 Pidsley R, Y Wong CC, Volta M, Lunnon K, Mill J, Schalkwyk LC. A data-driven approach to preprocessing Illumina 450K methylation array data. *BMC Genomics* 2013; **14**: 293.

19 Jaffe AE, Irizarry RA. Accounting for cellular heterogeneity is critical in epigenome-wide association studies. *Genome Biol* 2014; **15**: R31.

20 Janssen BG, Madlhoum N, Gyselaers W, *et al.* Cohort Profile: The ENVIRonmental influence ON early AGEing (ENVIR ON AGE): a birth cohort study. *Int J Epidemiol* 2017; : dyw269.

21 Lehne B, Drong AW, Loh M, *et al.* A coherent approach for analysis of the Illumina HumanMethylation450 BeadChip improves data quality and performance in epigenome-wide association studies. *Genome Biol* 2015; **16**: 37.

22 Johnson WE, Li C, Rabinovic A. Adjusting batch effects in microarray expression data using empirical Bayes methods. *Biostatistics* 2007; **8**: 118–27.

23 Vineis P, Chadeau-Hyam M, Gmuender H, *et al.* The exposome in practice: Design of the EXPOsOMICS project. *Int J Hyg Environ Health* 2017; **220**: 142–51.

24 Farchi S, Forastiere F, Vecchi Brumatti L, *et al.* Piccolipiù, a multicenter birth cohort in Italy: protocol of the study. *BMC Pediatr* 2014; **14**: 36.

25 Chatzi L, Leventakou V, Vafeiadi M, *et al.* Cohort Profile: The Mother-Child Cohort in Crete, Greece (Rhea Study). *Int J Epidemiol* 2017; **46**: 1392-1393k.

26 Keikkala E, Mustaniemi S, Koivunen S, *et al.* Cohort Profile: The Finnish Gestational Diabetes (FinnGeDi) Study. *Int J Epidemiol* 2020; **49**: 762-763g.

27 Nordlund J, Bäcklin CL, Wahlberg P, *et al.* Genome-wide signatures of differential DNA methylation in pediatric acute lymphoblastic leukemia. *Genome Biol* 2013; **14**: r105.

28 Niu L, Xu Z, Taylor JA. RCP: a novel probe design bias correction method for Illumina Methylation BeadChip. *Bioinformatics* 2016; **32**: 2659–63.

29 Xu Z, Niu L, Li L, Taylor JA. ENmix: a novel background correction method for Illumina HumanMethylation450 BeadChip. *Nucleic Acids Res* 2016; **44**: e20–e20.

30 Salas LA, Koestler DC, Butler RA, *et al.* An optimized library for reference-based deconvolution of whole-blood biospecimens assayed using the Illumina HumanMethylationEPIC BeadArray. *Genome Biol* 2018; **19**: 64.

31 Leek JT, Johnson WE, Parker HS, Jaffe AE, Storey JD. The sva package for removing batch effects and other unwanted variation in high-throughput experiments. *Bioinformatics* 2012; **28**: 882–3.

32 L’Abee C, Sauer PJ, Damen M, Rake J-P, Cats H, Stolk RP. Cohort Profile: The GECKO Drenthe study, overweight programming during early childhood. *Int J Epidemiol* 2008; **37**: 486–9.

33 Küpers LK, Xu X, Jankipersadsing SA, *et al.* DNA methylation mediates the effect of maternal smoking during pregnancy on birthweight of the offspring. *Int J Epidemiol* 2015; **44**: 1224–37.

34 Kooijman MN, Kruithof CJ, van Duijn CM, *et al.* The Generation R Study: design and cohort update 2017. *Eur J Epidemiol* 2016; **31**: 1243–64.

35 Kruithof CJ, Kooijman MN, van Duijn CM, *et al.* The Generation R Study: Biobank update 2015. *Eur J Epidemiol* 2014; **29**: 911–27.

36 Reinius LE, Acevedo N, Joerink M, *et al.* Differential DNA Methylation in Purified Human Blood Cells: Implications for Cell Lineage and Studies on Disease Susceptibility. *PLoS One* 2012; **7**: e41361.

37 Starling AP, Brinton JT, Glueck DH, *et al.* Associations of maternal BMI and gestational weight gain with neonatal adiposity in the Healthy Start study. *Am J Clin Nutr* 2015; **101**: 302–9.

38 Guxens M, Ballester F, Espada M, *et al.* Cohort Profile: The INMA—INfancia y Medio Ambiente—(Environment and Childhood) Project. *Int J Epidemiol* 2012; **41**: 930–40.

39 van Iterson M, Tobi EW, Slieker RC, *et al.* MethylAid: visual and interactive quality control of large Illumina 450k datasets. *Bioinformatics* 2014; **30**: 3435–7.

40 Herberth G, Hinz D, Röder S, *et al.* Maternal immune status in pregnancy is related to offspring’s immune responses and atopy risk. *Allergy* 2011; **66**: 1065–74.

41 Hinz D, Bauer M, Röder S, *et al.* Cord blood Tregs with stable FOXP3 expression are influenced by prenatal environment and associated with atopic dermatitis at the age of one year. *Allergy* 2012; **67**: 380–9.

42 Bauer T, Trump S, Ishaque N, *et al.* Environment‐induced epigenetic reprogramming in genomic regulatory elements in smoking mothers and their children. *Mol Syst Biol* 2016; **12**: 861.

43 Magnus P, Birke C, Vejrup K, *et al.* Cohort Profile Update: The Norwegian Mother and Child Cohort Study (MoBa). *Int J Epidemiol* 2016; **45**: 382–8.

44 Magnus P, Irgens LM, Haug K, Nystad W, Skjærven R, Stoltenberg C. Cohort profile: The Norwegian Mother and Child Cohort Study (MoBa). *Int J Epidemiol* 2006; **35**: 1146–50.

45 Rønningen KS, Paltiel L, Meltzer HM, *et al.* The biobank of the Norwegian mother and child cohort Study: A resource for the next 100 years. *Eur J Epidemiol* 2006; **21**: 619–25.

46 Reese SE, Xu C-J, den Dekker HT, *et al.* Epigenome-wide meta-analysis of DNA methylation and childhood asthma. *J Allergy Clin Immunol* 2019; **143**: 2062–74.

47 Håberg SE, London SJ, Nafstad P, *et al.* Maternal folate levels in pregnancy and asthma in children at age 3 years. *J Allergy Clin Immunol* 2011; **127**: 262-264.e1.

48 Joubert BR, Felix JF, Yousefi P, *et al.* DNA Methylation in Newborns and Maternal Smoking in Pregnancy: Genome-wide Consortium Meta-analysis. *Am J Hum Genet* 2016; **98**: 680–96.

49 Joubert BR, Håberg SE, Nilsen RM, *et al.* 450K Epigenome-Wide Scan Identifies Differential DNA Methylation in Newborns Related to Maternal Smoking during Pregnancy. *Environ Health Perspect* 2012; **120**: 1425–31.

50 Bibikova M, Barnes B, Tsan C, *et al.* High density DNA methylation array with single CpG site resolution. *Genomics* 2011; **98**: 288–95.

51 Teschendorff AE, Marabita F, Lechner M, *et al.* A beta-mixture quantile normalization method for correcting probe design bias in Illumina Infinium 450 k DNA methylation data. *Bioinformatics* 2013; **29**: 189–96.

52 Houseman EA, Accomando WP, Koestler DC, *et al.* DNA methylation arrays as surrogate measures of cell mixture distribution. *BMC Bioinformatics* 2012; **13**: 86.

53 Bakulski KM, Feinberg JI, Andrews S V., *et al.* DNA methylation of cord blood cell types: Applications for mixed cell birth studies. *Epigenetics* 2016; **11**: 354–62.

54 Hoyo C, Murtha AP, Schildkraut JM, *et al.* Folic acid supplementation before and during pregnancy in the Newborn Epigenetics STudy (NEST). *BMC Public Health* 2011; **11**: 46.

55 Hoyo C, Murtha AP, Schildkraut JM, *et al.* Methylation variation at IGF2 differentially methylated regions and maternal folic acid use before and during pregnancy. *Epigenetics* 2011; **6**: 928–36.

56 University of Oulu. University of Oulu: Northern Finland Birth Cohort 1986. http://urn.fi/urn:nbn:fi:att:f5c10eef-3d25-4bd0-beb8-f2d59df95b8e.

57 Jaaskelainen A, Schwab U, Kolehmainen M, *et al.* Meal frequencies modify the effect of common genetic variants on body mass index in adolescents of the northern Finland birth cohort 1986. *World Rev Nutr Diet* 2014; **109**: 10–1.

58 Hummel S, Much D, Rossbauer M, Ziegler A-G, Beyerlein A. Postpartum Outcomes in Women with Gestational Diabetes and their Offspring: POGO Study Design and First-Year Results. *Rev Diabet Stud* 2013; **10**: 49–57.

59 Fortin J-P, Fertig E, Hansen K. shinyMethyl: interactive quality control of Illumina 450k DNA methylation arrays in R. *F1000Research* 2014; **3**: 175.

60 Send TS, Gilles M, Codd V, *et al.* Telomere Length in Newborns is Related to Maternal Stress During Pregnancy. *Neuropsychopharmacology* 2017; **42**: 2407–13.

61 Witt SH, Frank J, Gilles M, *et al.* Impact on birth weight of maternal smoking throughout pregnancy mediated by DNA methylation. *BMC Genomics* 2018; **19**: 290.

62 Girchenko P, Hämäläinen E, Kajantie E, *et al.* Prediction and Prevention of Preeclampsia and Intrauterine Growth Restriction (PREDO) study. *Int J Epidemiol* 2016; : dyw154.

63 Morin AM, Gatev E, McEwen LM, *et al.* Maternal blood contamination of collected cord blood can be identified using DNA methylation at three CpGs. *Clin Epigenetics* 2017; **9**: 75.

64 Price EM, Cotton AM, Lam LL, *et al.* Additional annotation enhances potential for biologically-relevant analysis of the Illumina Infinium HumanMethylation450 BeadChip array. *Epigenetics Chromatin* 2013; **6**: 4.

65 Almqvist C, Örtqvist AK, Ullemar V, Lundholm C, Lichtenstein P, Magnusson PKE. Cohort Profile: Swedish Twin Study on Prediction and Prevention of Asthma (STOPPA). *Twin Res Hum Genet* 2015; **18**: 273–80.

66 Anckarsäter H, Lundström S, Kollberg L, *et al.* The Child and Adolescent Twin Study in Sweden (CATSS). *Twin Res Hum Genet* 2011; **14**: 495–508.

67 Magnusson PKE, Almqvist C, Rahman I, *et al.* The Swedish Twin Registry: Establishment of a Biobank and Other Recent Developments. *Twin Res Hum Genet* 2013; **16**: 317–29.

68 Oken E, Baccarelli AA, Gold DR, *et al.* Cohort Profile: Project Viva. *Int J Epidemiol* 2015; **44**: 37–48.

69 Peng C, Cardenas A, Rifas-Shiman SL, *et al.* Epigenome-wide association study of total serum immunoglobulin E in children: a life course approach. *Clin Epigenetics* 2018; **10**: 55.

70 Sandoval J, Heyn H, Moran S, *et al.* Validation of a DNA methylation microarray for 450,000 CpG sites in the human genome. *Epigenetics* 2011; **6**: 692–702.

# **Analytical plan**

Maternal educational attainment is an important indicator of socio-economic status. It has also been observed to be an important predictor of offspring health outcomes across the life course, such as obesity and type 2 diabetes, and cognitive function. However, the underlying biological mechanisms remain poorly understood. We aim to explore associations of maternal educational attainment with offspring methylation markers in cord blood, childhood and adolescence whole blood.

*Maternal education attainment measures:*

Maternal education (‘Matedu’) will be harmonised across the cohorts. The variable is defined in accordance with the ISCED 1997 classification (1997), leading to seven categories of educational attainment that are internationally comparable. For details about how your country’s educational system and the information in your survey relates to these categories, please refer to http://www.uis.unesco.org/Education/ISCEDMappings/Pages/default.aspx.

| **Levels** | **Definition** | **Years of schooling** |
| --- | --- | --- |
| 0 | Pre-primary education | 1 |
| 1 | Primary education or first stage of basic education | 7 |
| 2 | Lower secondary or second stage of basic education | 10 |
| 3 | (Upper) secondary education | 13 |
| 4 | Post-secondary non-tertiary education | 15 |
| 5 | First stage of tertiary education (not leading directly to an advanced research qualification) | 19 |
| 6 | Second stage of tertiary education (leading to an advanced research qualification, e.g. a Ph.D.) | 22 |

This measure harmonizes observations across cohorts and countries and maintains a high level of variance in the phenotype. First, transform the highest educational attainment of your subjects into the appropriate ISCED 1997 level. Then, impute the appropriate years-of-schooling equivalent, as seen in the above table. All further steps need to be conducted using the years-of-schooling equivalents (not the numeric values of the ISCED levels). As an example for this transformation: if an individual has education level 4 in any country or school system, then the years of schooling to be used in analyses is equal to 15. If your data does not allow you to differentiate between levels 5 and 6, code everyone with a tertiary education as level 5 and impute 20 years of schooling instead of 19 for everyone at this level.

The analysis will be conducted on the standardized US years-of-schooling equivalents in your sample.

*Methylation markers:*

The analysis will be divided into two different types of methylation markers measurements:

- Cord blood and,
- Whole blood in childhood and adolescence
- Untransformed beta values will be used as the outcome variable. Please normalize the untransformed beta values using your preferred normalization package, and use your preferred study QC settings for probe filtering. Trimmed betas should be used. Please use the 3IQR trimming method and include a log file that contains the number of CpG sites for each probe that were excluded after trimming.

*Covariates:*

- Batch: Adjustment for batch effects should be done by including the most important covariate(s) (for example, plate) in the models. Alternatively, a batch correction method such as ComBat can be used. Please indicate in the README file how you adjusted for batch.
- Cell type:
- For cord blood DNA: Estimated cell types: Use the “Salas” reference set for cell type estimation in the ‘’FlowSorted.CordBlood.Combined.450K’’ or “FlowSorted.CordBlood.Combined.EPIC’’ Bioconductor package for cell type correction. These packages include the following cell types: CD8T, CD4T, NK, Bcell, Mono, Gran, nRBC^[[1]](#footnote-1)^. If you need a script for estimating these, please contact j.felix@erasmusmc.nl
  - For Peripheral blood leukocyte (PBL) DNA at older ages: please use “Blood” in the same packages and include the 6 estimated cell types (CD8T, CD4T, NK, Bcell, Mono, Gran)
- Selection factors: optional covariate. Please include if relevant for your study, for example if your sample was selected based on case or control status of some condition, please include the case/control variable.
- Ancestry: If your study has more than one major ethnic group (for example, European ancestry, Latino, African Ancestry, Asian), analyse them separately.
  - If sample sizes are too small for certain ethnicities, please drop. If in doubt, please contact.
- Maternal age (‘Matage’): Continuous (in years)
- Maternal smoking: Sustained smoking during pregnancy vs no
- Gestational age: continuous (in weeks)
- Pre-pregnancy maternal BMI** (‘Maternal BMI’): BMI should be measured in kg/m^2^
- Child BMI*: BMI should be measured in kg/m^2^
- Child sex: 1=males, 2=female
- Child age*: in years
- Child smoking*: categorised as 1=No, 2=Yes,
- Please contact us if you feel there are other covariates that need to be included.
- *For whole blood analysis
- ** if only BMI during pregnancy is available in your cohort, be in touch.

*Exclusions:*

- Exclude multiple births (i.e. singleton only analysis) and children with known congenital anomalies at birth.
- Exclude siblings (1 child per mother included) or use appropriate family-based methods.

*Epigenome-wide association studies:*

Robust linear regression modelling (rlm() option in R) for each CpG site individually.

Example R script is provided below

Following models will be used:

1. CpG = Matedu_years + batch + cell types (+selection factors) + child sex
2. CpG = Matedu_years + batch + cell types (+selection factors) + child sex + Matage + Maternal BMI + Maternal smoking + Gestational age
3. CpG** = Matedu_years + batch + cell types (+selection factors) + child sex + Matage + Maternal BMI + Maternal smoking + Gestational age + Child BMI + Child smoking + Child age (if applicable)

**Model 3 will be used in whole blood analysis at childhood and adolescence

Use complete case analysis

- Please do not adjust your P-values for multiple testing

*Data File Format*

- Please name files as specified as: MODEL_AGE_STUDY_DATE.txt
  - MODEL = MODEL1, MODEL2, as above
  - AGE = CORD or for example 6y
  - STUDY = short study identifier
  - DATE=YYYYMMDD
  - Examples: MODEL1_CORD_GENR_20190712 or MODEL1_16y_NFBC1986_20190712
- Missing data should be denoted by NA. Please do not leave any cells blank.
- Do not use quotes around data cells or headers.
- Files should be whitespace delimited, one row per probe. First row is a header with labels as defined in the table below:

| **Column Header** | **Description** | **Format** | **Examples** |
| --- | --- | --- | --- |
| probeID | Probe name | String | cg23094576 |
| BETA | Effect size | Numeric, at least 4 meaningful digits | 0.2036 |
| SE | Standard error of the beta | Numeric, at least 4 meaningful digits | 0.5611 |
| P_VAL | P value for the probe | Scientific notation, at least 4 meaningful digits | 3.244E-10 |
| N | Sample size for analysis at each probe | Numeric | 661 |

- README file: With your data, please upload a readme file with a short description of:
  - the normalization and QC steps taken in your study
  - information on the exposure and covariates
  - Any additional information about the uploaded files
  - The total sample size and lambda for each of your analyses
- Please provide cohort descriptives in the attached excel sheet with the name: CohortName_Info_DDMMYYYY_InitialsAnalyst and rename the file accordingly

*Abbreviations*

| BETA | Estimate |
| --- | --- |
| Matage | Maternal age |
| Matedu_years | Maternal education in US years |
| Maternal BMI | Maternal pre-pregnancy BMI |
| N | Sample size |
| P_Val | P value |
| PBL | Peripheral Blood Leukocyte |
| QC | Quality Control |
| SE | Standard Error |

**Example R code**

## Maternal education

## Example R code

# Please check first:

# your methylation data has the following format: rows=samples,

# columns=methylation sites

# your phenotype data and methylation data have the same number of rows

# (samples), and are in the exact same order

# in this example the methylation data is called 'beta' and the

# phenotype data is called 'PHENO'

library(data.table)# to process results

library(MASS) # rlm function for robust linear regression

library(sandwich) #HuberÌs estimation of the standard error

library(lmtest) # to use coeftest

library(parallel) # to use multicore approach - part of base R

# Function for running the model (in this example, there are 6

# covariates in addition to batch

# - any amendments to this must be made in the function and also when it

# is called below)

robreg <- function(meth_matrix,methcol,exposure, X1, X2, X3, X4, X5, X6, X7, batch){

cf = tryCatch({

mod = rlm(meth_matrix[, methcol]~exposure+X1+X2+X3+X4+X5+X6+X7+batch,maxit=200)

cf = coeftest(mod, vcov=vcovHC(mod, type="HC0"))

N <- length(mod$fitted.values)

cf <- cf[2, c("Estimate", "Std. Error", "Pr(>|z|)")]

cf <- append(cf, N); names(cf)[4] <- "N"

cf

}, error=function(err){

cf <- c(NaN, NaN, NaN, NaN)

names(cf) <- c("Estimate", "Std. Error", "Pr(>|z|)", "N")

cf

});

cf

}

# Run adjusted EWAS

# Adapt this part to specify the variable labels in your phenotype data

ind.res <- mclapply(setNames(seq_len(ncol(beta)), dimnames(beta)[[2]]), robreg,

meth_matrix = beta,

exposure = PHENO$ME,

X1 = PHENO$gender,

X2 = PHENO$CD8T,

X3 = PHENO$CD4T,

X4 = PHENO$NK,

X5 = PHENO$Bcell,

X6 = PHENO$Mono,

X7 = PHENO$Gran,

batch = PHENO$batch)

# Process results

setattr(ind.res, 'class', 'data.frame')

setattr(ind.res, "row.names", c(NA_integer_,4))

setattr(ind.res, "names", make.names(names(ind.res), unique=TRUE))

probelistnames <- names(ind.res)

all.results <- t(data.table(ind.res))

all.results <-data.table(all.results)

all.results[, probeID := probelistnames]

# rename columns

setnames(all.results, c("BETA","SE", "P_VAL", "N", "probeID"))

setcolorder(all.results, c("probeID","BETA","SE", "P_VAL", "N"))

all.results <- all.results[which(!is.na(all.results$BETA)), ]

rm(probelistnames, ind.res)

# Export table of results

write.table(all.results, "MODEL_AGE_STUDY_DATE.txt",na="NA", row.names = F, quote=F)

##################################################################################

# Calculate lambda

Lambda <- qchisq(median(all.results$P_VAL,na.rm=T), df = 1, lower.tail = F)/

qchisq(0.5, 1)

## Save model number, sample size & lambda to text file:

Model <- 1

TotalSampleSize <- dim(PHENO[complete.cases(PHENO),])[1]

tab <- cbind(Model, TotalSampleSize, Lambda)

write.table(tab, file="MODEL1_SAMPLESIZELAMBDA.txt", row.names=F, quote=F, sep="\t")

###################################################################################

# Summarize probes

descriptives <- function(x){

tmp <- c(min(x,na.rm=T),quantile(x,probs=c(.1,.25,.5),na.rm=T), mean(x,na.rm=T),sd(x,na.rm=T),quantile(x,probs=c(.75,.90),na.rm=T), max(x,na.rm=T),sum(is.na(x)))

names(tmp)[c(1,4:6,9:10)] <-c("Min.","Median","Mean","SD","Max.","NA")

return(tmp)

}

desc <- t(apply(beta,2,descriptives))

write.table(desc, file = "STUDY_PROBEDESCRIPTIVES.txt", sep = "\t", col.names = T, row.names = T, append = F, quote=FALSE)

#################### Code for trimming###############################

# Code for trimming methylation beta values to remove potential outliers

# Trimming scheme is as follows – trim values beyond the lower and upper # outer fences. These are defined by:

# Values < 25th percentile minus 3*IQR AND Values > 75th percentile plus # 3*IQR

#(IQR = interquartile range)

# The trimming should be done on the file that you use for PACE

# analyses. For most cohorts this is normalized beta values.

# The following code was provided by Gemma Sharp on June 12, 2015 –

# modification (to run faster) of code provided earlier by Janine Felix. # Input also from Allan Just.

#Function

removeOutliers<-function(probes){

require(matrixStats)

if(nrow(probes) < ncol(probes)) warning("expecting probes are rows (long dataset)")

rowIQR <- rowIQRs(probes, na.rm = T)

row2575 <- rowQuantiles(probes, probs = c(0.25, 0.75), na.rm = T)

maskL <- probes < row2575[,1] - 3 * rowIQR

maskU <- probes > row2575[,2] + 3 * rowIQR

initial_NAs<-rowSums(is.na(probes))

probes[maskL] <- NA

removed_lower <- rowSums(is.na(probes))-initial_NAs

probes[maskU] <- NA

removed_upper <- rowSums(is.na(probes))-removed_lower-initial_NAs

N_for_probe<-rowSums(!is.na(probes))

Log<-data.frame(initial_NAs,removed_lower,removed_upper,N_for_probe)

return(list(probes, Log))

1. [↑](#footnote-ref-1)
